# Privileged nitrogen heterocycles in anticancer drug discovery: recent advances on imidazole, indole, and pyrimidine scaffolds

**DOI:** 10.1039/d6ra02591a

**Published:** 2026-07-02

**Authors:** Maithreyi Govindarajan, Lalmohan Maji, Subramaniyan Mannathan, Kathiravan Muthu Kumaradoss, Arpan Chowdhury, Seetharama D. Jois, Baburaj Baskar

**Affiliations:** a Laboratory of Sustainable Chemistry, Department of Chemistry, College of Engineering and Technology, SRM Institute of Science and Technology Kattankulathur 603203 Tamil Nadu India baskarb@srmist.edu.in; b Department of Pharmaceutical Chemistry, SRM College of Pharmacy, Faculty of Medicine and Health Sciences, SRM Institute of Science and Technology Kattankulathur, Chengalpattu District Tamil Nadu 603203 India; c Department of Chemistry, SRM University-AP Amaravati Andhra Pradesh 522240 India; d Dr APJ Abdul Kalam Research Laboratory, SRM College of Pharmacy, Faculty of Medicine and Health Sciences, SRM Institute of Science and Technology Kattankulathur, Chengalpattu District Tamil Nadu 603203 India kathirak@srmist.edu.in; e Department of Pathobiological Sciences, School of Veterinary Medicine, Louisiana State University Baton Rouge LA 70803 USA

## Abstract

Imidazoles, indoles, and pyrimidines are promising heterocyclic scaffolds for anticancer drug discovery. Based on the literature from 2020 to 2025, this review summarises that structurally diverse imidazoles, particularly ether-linked and long-chain variants, exhibit robust anticancer activity through multi-target protein inhibition and minimal off-target effects. Indoles trigger apoptosis and inhibit angiogenesis, while pyrimidines and their hybrids exhibit nanomolar potency and often outperform conventional chemotherapy. These scaffolds collectively modulate important pathways in cancer and exhibit enhanced selectivity toward tumour cells; however, clinical translation is challenging mainly due to incomplete mechanism of action and pharmacokinetics. Further rational optimisation and green synthesis methods may accelerate the development of these heterocycles into next-generation anticancer agents.

## Abstract 2

Imidazoles, indoles, and pyrimidines are promising heterocyclic scaffolds for anticancer drug discovery.

## Graphical abstract

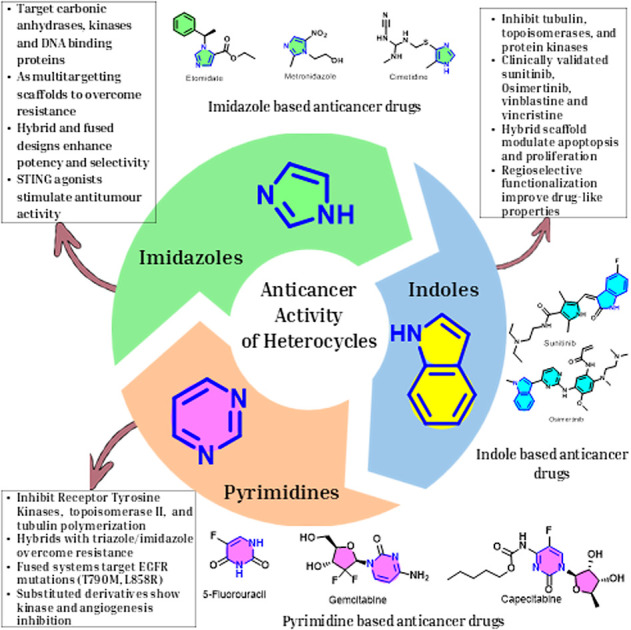

## 1. Introduction

Cancer is a multi-factorial disease that is defined by uncontrolled cell growth, loss of cell boundaries, and metastasis. It results from the steady growth of mutations in oncogenes, cancer suppressing genes, and DNA restore mechanisms.^1,2^ Today, cancer is still one of the main causes of morbidity and mortality worldwide, and this is only expected to worsen in the coming years as the population increases.^3,4^ Additionally, state-of-the-art chemotherapeutic drugs are linked with significant limitations, including severe drug toxicity and drug resistance.

Amidst these challenges, the need for rational design of mechanism-directed anticancer agents is growing. The goal is higher specificity for malignant cells with improved safety and tolerability. Within this context, heterocyclic core scaffolds are considered essential tools in medicinal chemistry as they provide the ability to optimise crucial pharmacokinetic and pharmacodynamic properties such as solubility, metabolic stability, affinity, and specificity.^5^ Privileged scaffolds are structural frameworks capable of providing high-affinity ligands for diverse biological targets through rational and minimal structural modifications. Nitrogen heterocycles are the best examples of privileged structures found in various bioactive natural products and approved anticancer agents.^6,7^

Among these, the imidazole, indole, and pyrimidine scaffolds have been of particular interest as privileged scaffolds in modern anticancer drug development. Five-membered heterocyclic imidazole ring contains two nitrogen atoms at the 1 and 3 positions with in the ring framework.^8^ Imidazole represents useful heterocyclic moiety that has been extensively found in many natural and synthetic molecules.^9^ The incorporation of an imidazole ring is a well-established strategy into bioactive compounds in medicinal chemistry for improving water solubility. This effect is primarily attributed to the presence of two nitrogen atoms within the imidazole moiety which promote to formation of hydrogen bond.^10^ Imidazole scaffold is a privileged structural motif in medical chemistry, and it is significantly involved in different biological molecules and biomolecules process. The imidazole nucleus is a key structural motif present in several important compounds of the human organism, including biotin, histidine, and histamine.^11^ Imidazole-containing compounds demonstrated broad range of pharmacological properties, including anticancer, antiviral, anti-bacterial, anti-fungal, anti-inflammatory, anti-tuberculosis, and antidiabetic effects. In recent year, imidazoles derivatives have been reported as targeted therapeutic agents particular as potential enzyme inhibitors and ion channel modulators.^12–15^ Imidazole derivatives, such as ether-linked and long-chain derivatives, function as efficacious multitarget inhibitors with significant tumour selectivity.^15,16^ Their design aligns closely with modern hybrid-drug concepts and targeted kinase inhibition strategies. Indole is a ubiquitous naturally found heterocyclic compound that consist of a six-membered benzene ring with five-membered pyrrole ring.^17,18^ Indole exists in three principal tautomeric forms, 1*H*, 2*H* and 3*H* indole.^19^ The conjugated π-electron system confers pronounced aromatic stabilization, while the existence of a mildly acidic N–H moiety has major influences on its chemical behaviour. These structural features govern the reactivity profile of indole, allowing it to be involved in both electrophilic and nucleophilic substitution reactions.^20^ The indole ring represents a privileged structural moiety in medicinal chemistry, exhibiting a wide range of pharmacological activities. Indole based derivatives demonstrated significant antifungal, antihistaminic, anti-inflammatory, antioxidant, antimicrobial, plant growth regulator, anticonvulsant, anti-HIV, and analgesic activity.^21–23^ Indoles exhibit robust apoptosis-inducing and anti-angiogenic properties, interfere in many different cancer-relevant pathways, and continue to serve as valuable scaffolds for target-based optimisation directed at diverse proteins, including kinases, tubulin, and other signal transducers.^24,25^ Pyrimidines are six-membered aryl heterocyclic compounds containing two nitrogen atoms located at the 1- and 3-positions of the ring, with a molecular formula of C_4_H_4_N_2_.^26^ Pyrimidine name was first given by Pinner by combining the term “pyridine” and “amidine”.^27^ In medicinal chemistry, pyrimidine and its derivatives are a privileged heterocyclic scaffold exhibiting wide range of pharmacological actions. Due to the structural diversity, pyrimidine derivatives were reported as an anti-inflammatory, analgesic, antioxidant, anticonvulsant, anti-Alzheimer, anti-HIV, anti-hepatitis, antidepressant, anti-hyperglycemic, antiviral, anti-Parkinson, antiplasmodial, antimicrobial, antitubercular, anticancer, antithelminthic, and antimalarial activity and anti-acetylcholinesterase properties.^28–33^ The pyrimidine nucleus is a strategically significant pharmacophore for the design and development of therapeutic agents with multi-target potential. Pyrimidine scaffolds and their molecular hybrids frequently achieve nanomolar antiproliferative potency *in vitro*.^34,35^ This high efficacy is largely attributed to their structural homology with natural nitrogenous bases, which facilitates high-affinity kinase inhibition *via* ATP-binding site competition, direct DNA intercalation, and the disruption of essential oncogenic signalling cascades.

Despite the encouraging leads, the clinical development of a number of candidates based on the imidazole, indole, and pyrimidine cores are often hindered by incomplete knowledge of the underlying mechanism, suboptimal pharmacokinetics, and issues like off-target effect, together with a lack of efficacy within the *in vivo* model.^10,33,36^ Addressing these shortcomings required the integration of the structure–activity relationship analysis, target-focused design, and computer-aided design methods like molecular docking, quantitative structure–activity relationship modelling, pharmacophore alignment, and ADMET (Absorption, Distribution, Metabolism, Excretion, and Toxicity) prediction. Simultaneously, green and efficient synthetic methods are becoming vital for accelerating lead optimisation, streamlining the transition from discovery to preclinical and clinical trials.

In this respect, this literature review focuses on the design, synthesis, structural diversification, and anticancer properties of imidazole, indole, and pyrimidine skeletons based on the literature published between 2020 and 2025. The purposes of this literature review are:

(i) Identifying the primary molecular targets modulated by these nitrogen-containing heterocycles,

(ii) Summarizing recent progress in the design of hybrid and multitarget ligands based on these skeletons, and.

(iii) Discussing future directions for optimizing these privileged scaffolds into next-generation anticancer drugs with enhanced selectivity and pharmacokinetic profiles.

## 2 Imidazoles

Imidazole-based compounds represent a significant class of small molecules in the fight against cancer, highlighting diverse mechanisms of action that make them attractive candidates for therapeutic development.^37,38^ Their distinct structural features allow them to interact with a wide range of biological targets, including enzymes such as kinases, carbonic anhydrases, and DNA-associated proteins, which are crucial for cancer cell proliferation, survival, and metastasis. Recent studies have highlighted the potential of imidazole derivatives not only as direct cytotoxic agents but also as multi-targeting scaffolds capable of addressing drug resistance through hybrid designs that combine imidazole with other pharmacophores.^39,40^ Moreover, novel imidazole hybrids and fused heterocycles have been synthesized to enhance anticancer activity and selectivity, with several compounds demonstrating promising *in vitro* and *in vivo* efficacy.^41,42^ In addition, imidazole frameworks have been explored as potent immune-modulating agents, including STING (Stimulator of Interferon Genes) agonists that stimulate antitumor immune responses, thus broadening their therapeutic applications.^43^ Collectively, these findings establish imidazoles as versatile molecular platforms for anticancer drug discovery, offering opportunities to design next-generation therapeutics with improved potency, selectivity, and resistance profiles.

### 2.1 Imidazole-based ethers: synthesis and dual-targeting potential as antioxidants, anticancer agents, and carbonic anhydrase I–II inhibitors

**Fig. 1:**
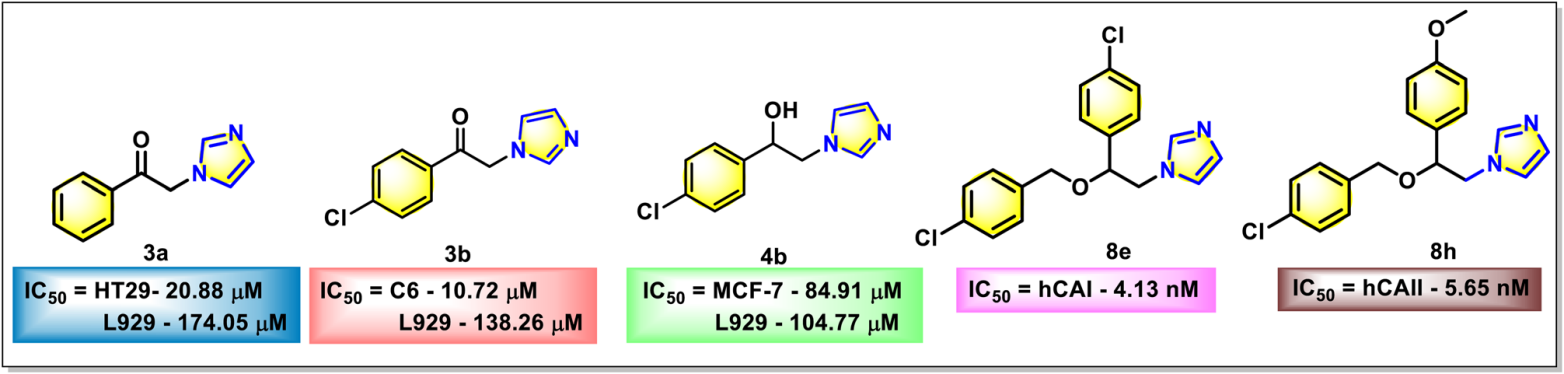
Chemical structures and biological profiles of the most potent imidazole-based derivatives synthesized by Faris *et al.*^37^

**Fig. 2:**
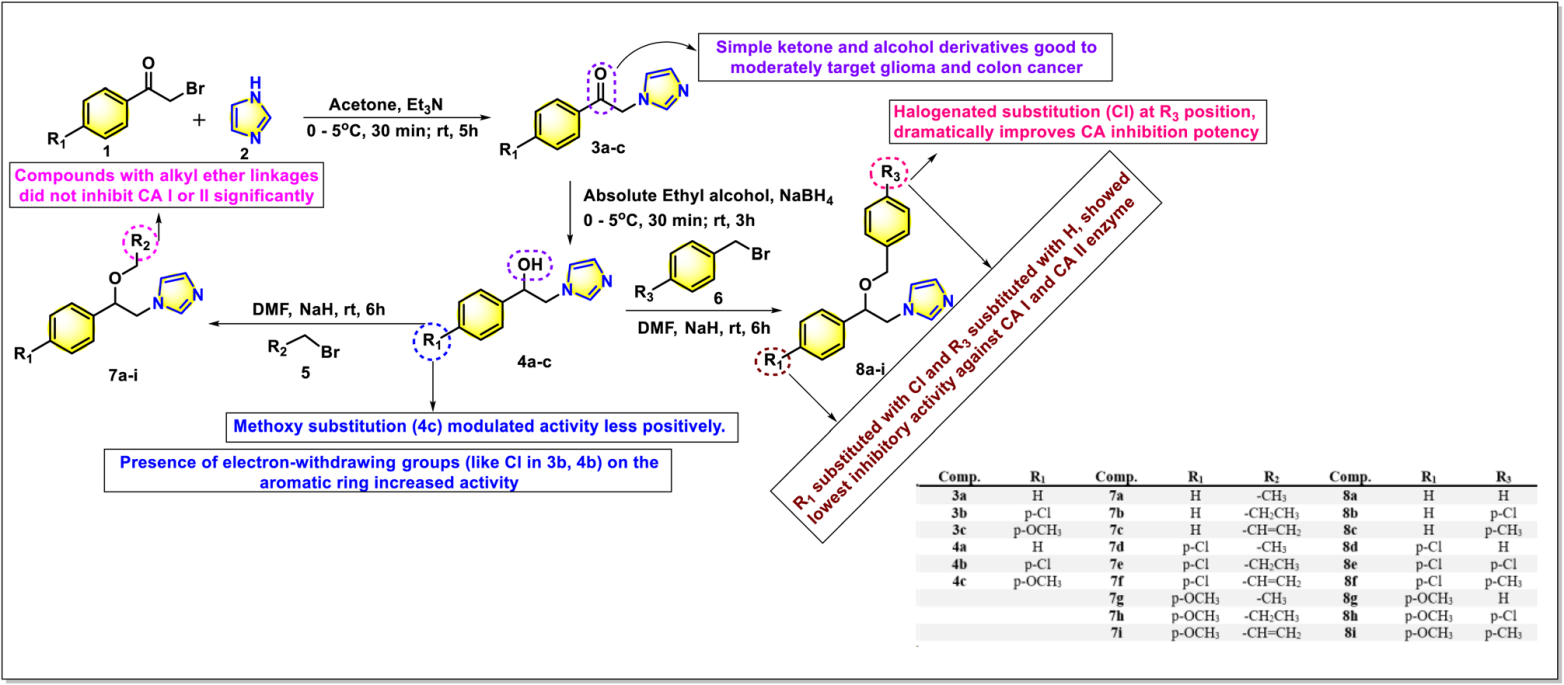
Synthesis of alkyl (7a–i) and aryl (8a–i) ether derivatives,^37^ reproduced from ref. 37 under the terms of the Creative Commons Attribution 4.0 license (CC BY 4.0), Copyright 2024, M. Faris, H. E. Bostancı, İ. Özcan, M. Öztürk, Ü. M. Koçyiğit, T. Erdoğan, H. Tahtaci.

Building on the extensive pharmacological applications of imidazole scaffolds, researchers have pursued various structural modification approaches to improve their efficacy as enzyme inhibitors and anticancer agents (Fig. 1). In this context, Faris *et al.* (2024) reported eighteen imidazole-derived compounds, of which nine are novel ether derivatives (7a–i) and (8a–i) featuring substituted phenyl and imidazole rings (Fig. 2). These compounds were synthesized by treating imidazole precursors either with alkyl, vinyl or benzyl halides using potassium carbonate in a polar aprotic solvent to achieve efficient *O*-alkylation. All synthesized compounds were evaluated for their inhibitory potential against human carbonic anhydrase (hCA) isozymes and antiproliferative activity against MCF-7 (breast cancer), C6 (rat glioblastoma), and HT-29 (colon cancer) cell lines. Compound 8e exhibited potent inhibition of cytosolic hCA I with an IC_50_ (Half-maximal Inhibitory Concentration) of 4.13 nM, while compound 8h effectively inhibited cytosolic hCA II with an IC_50_ of 5.65 nM.^37^

The C6 glioma cell line exhibited the highest sensitivity level to compound 3b (IC_50_ = 10.72 µM) while the HT29 colon cancer cell line showed a preference towards compound 3a (IC_50_ = 20.88 µM). However, these particular compounds did not have a substantial effect on the MCF-7 cell line. Among the synthesized compounds, compound 4b was found to have the highest sensitivity level on the MCF-7 cell line but with a significantly higher IC_50_ value of 84.91 µM. To understand the mechanistic roles, a flow cytometry analysis was carried out. The results demonstrated that treatment of C6 cells with the IC_50_ dose of compound 3b induced both early and late apoptosis. Furthermore, the compound elicited cell cycle arrest at the G0/G1 phase, preventing the progression of the C6 cells into the synthesis (S) phase.^37^

### 2.2 Evaluation of the anticancer potential of imidazole-based ionic liquids and lysosomotropic detergents: *in silico* and *in vitro* investigations

**Fig. 3:**
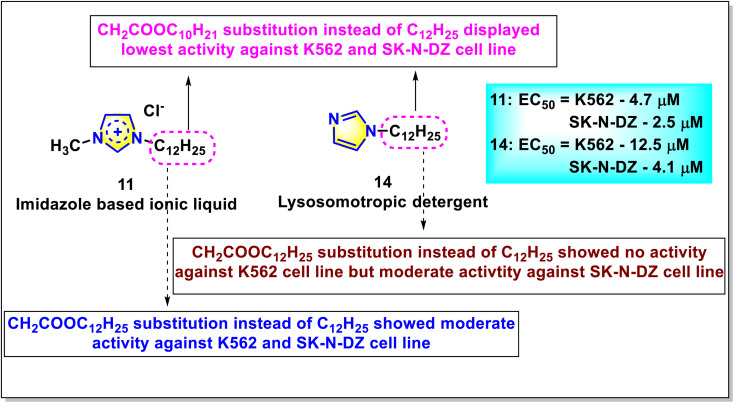
Structural motifs and anti-proliferative activities of the highly effective imidazole-containing ionic liquids and lysosomotropic detergents synthesized by Gryniukova *et al.*^44^

**Fig. 4:**
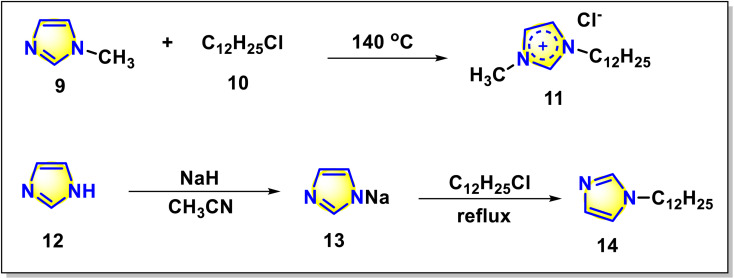
Synthesis of long-chain imidazole-based ionic liquids and lysosomotropic detergents,^44^ reproduced from ref. 44 with permission from Springer Nature, *Mol. Diversity*, 2024, **28**, 3817–3833, Copyright 2024.

Long-chain imidazolium salts, recognized for their antibacterial, antifungal, and antibiofilm properties, have demonstrated efficacy in inhibiting human tumour cell growth (Fig. 3). Gryniukova *et al.* (2024) synthesized these ionic liquids *via* quaternization of imidazole cores with alkyl halides bearing extended hydrophobic chains (Fig. 4). Their inhibitory activity against leukaemia and neuroblastoma was expected *via in silico* QSAR models. Molecular docking further elucidated their anticancer mechanisms through interactions with key targets, including Sirtuin-1 (SIRT1), bromodomain-containing protein 4 (BRD4), Aurora kinase A, and Janus kinase 2 (JAK2).^44^

Among the synthesized compounds, 11 and 14 exhibited the most potent cytotoxic effects against both cancer cell lines. These long-chain imidazole derivatives showed considerable cytotoxic activity against SK-N-DZ neuroblastoma and K562 leukaemia cells, through the inhibition of multiple molecular targets critical to the proliferation of cancer cells. These findings highlights the therapeutic potential of lysosomotropic detergents and long-chain imidazole-based ionic liquids for treating neuroblastoma and chronic myeloid leukaemia.^44^

### 2.3 Anticancer and antimicrobial activities of quinoline–imidazole derivatives as dual-/multi-target hybrid inhibitors

**Fig. 5:**
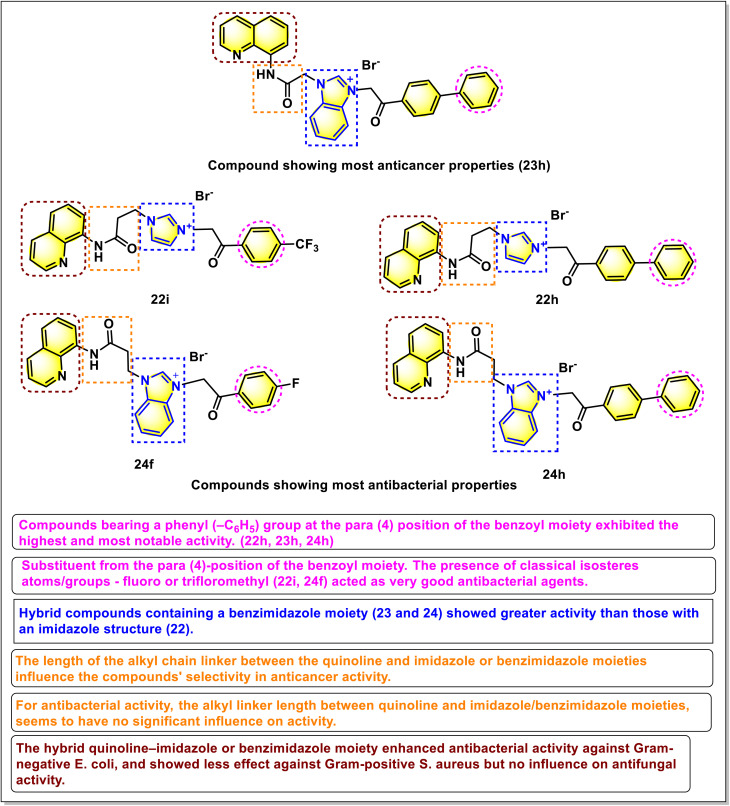
Structures and structure–activity relationship (SAR) analysis of most potent quinoline–imidazole/benzimidazole based hybrids synthesized by Diaconu D. *et al.*^45^

**Fig. 6:**
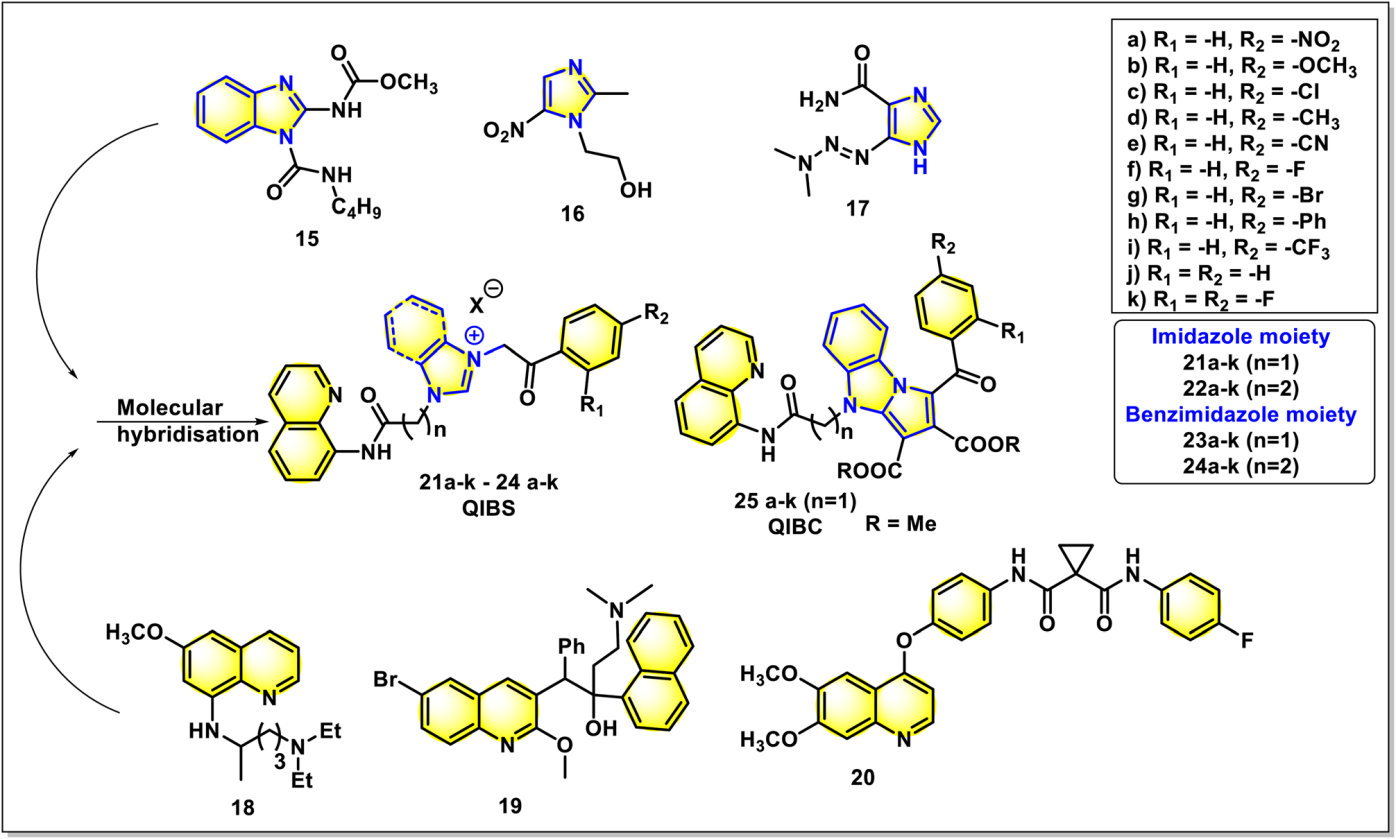
Design strategy and molecular hybridization of quinoline–imidazole/benzimidazole derivatives (QIBS and QIBC),^45^ reproduced from ref. 45 under the terms of the Creative Commons Attribution 4.0 license (CC BY 4.0), Copyright 2022, D. Diaconu, V. Antoci, V. Mangalagiu, D. Amariucai-Mantu and I. I. Mangalagiu.

The integration of quinoline with imidazole or benzimidazole pharmacophores into hybrid structures has attracted significant interest as a strategy to enhance and expand biological activity (Fig. 5). Exploiting this approach, Diaconu D. *et al.* (2022) reported two new hybrid quinoline–imidazole/benzimidazole derivatives (QIB Salt & QIB compounds) to investigate their antibacterial and anticancer properties. The targeted compounds were synthesized through a sequential *N*-acylation, *N*-alkylation, quaternisation, and Huisgen [3 + 2] cycloaddition reactions (Fig. 6). Among the library of 46 hybrid quinoline–benzimidazole derivatives evaluated in a single-dose *in vitro* anticancer screen, the compound QIBS 23h identified as the lead candidate.

This compound demonstrated significant potency as a Potential Genotoxic Impurity (PGI) (90–100%), while simultaneously exhibiting exceptional cytotoxicity across all tested cancer cell lines. Leukaemia HL-60 TB and breast cancer MDA-MB-468 are two cancer cell lines that respond well to three additional quinoline–imidazole/benzimidazole hybrids (22h, 24h, and 24f). Three hybrid QIBS (24f, 24c, and 24d) show antimicrobial properties against the Gram-negative bacteria *E. coli*, and one compound (22i (R_2_ = –CF_3_)), is effective against the Gram-positive bacteria *S. aureus*. QIBC with a benzoyl moiety bearing aryl group (R_2_ = –C_6_H_5_) at the para position are a suitable candidate for future drug development because of all these wonderful qualities that combat bacteria and cancer.^45^

### 2.4 Anticancer activities of celastrol–imidazole derivatives

**Fig. 7:**
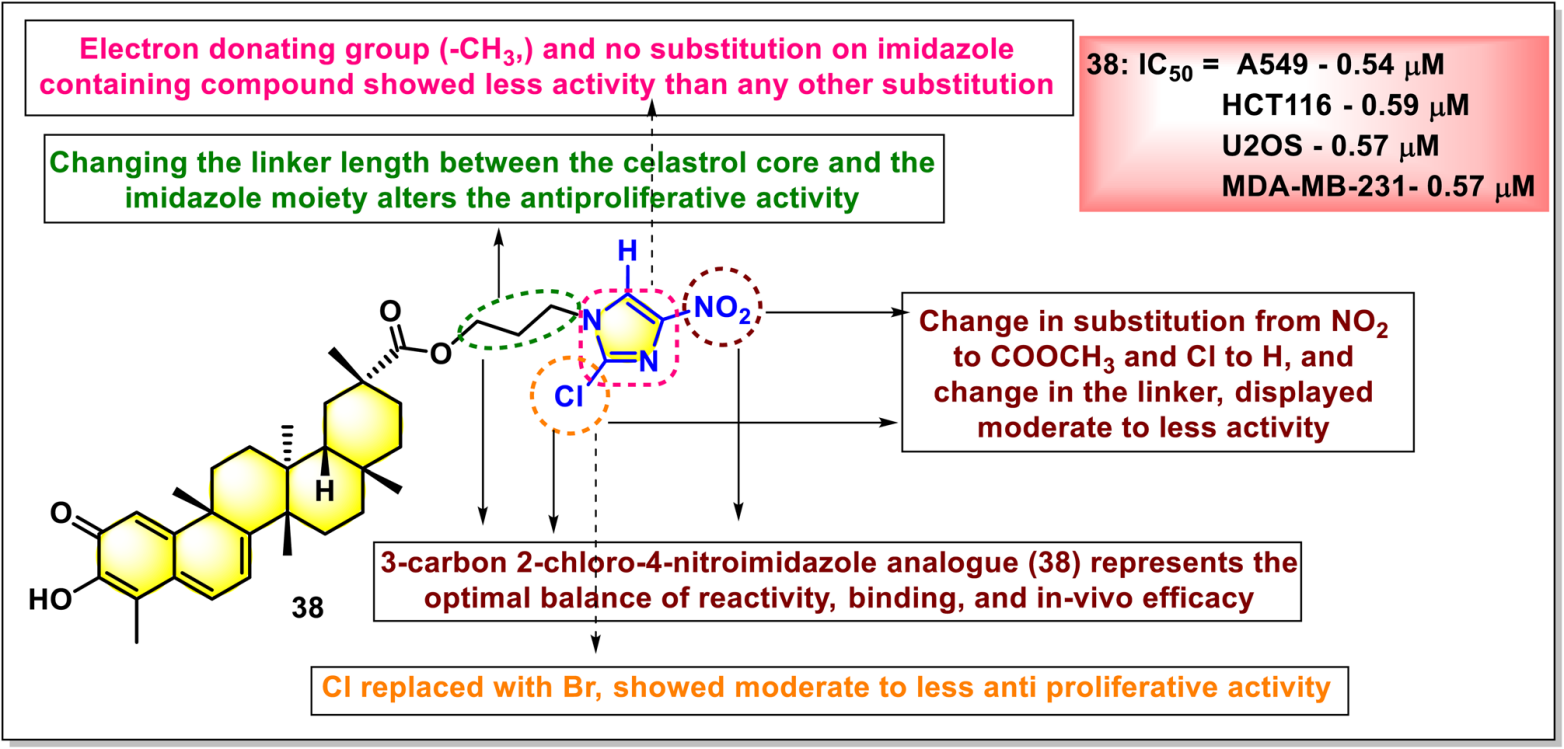
Structure activity relationship and bioactivity of the most potent derivative synthesised by Li *et al.*^39^

Combining bioactive natural compounds with imidazole frameworks has become an attractive approach to improve target specificity and increase anticancer effectiveness. In line with this approach, Li *et al.* (2022) explored the development of new celastrol–imidazole compounds as potential anticancer agents. Significant efforts were paid to improve the Hsp90-Cdc37 inhibitory characteristics of celastrol (CEL) by attaching various substituted imidazoles to the C-20-COOH position. A total of twenty-eight novel celastrol derivatives were synthesised and screened for their antiproliferative properties against various cancer cell lines (A549, HCT116, U2OS, and MDA-MB-231). Among them, compound 38 demonstrated superior antiproliferative effects, covalent-binding capacity, and inhibition of Hsp90-Cdc37 compared to celastrol.^39^ The research examined the binding locations and docking mechanism of compound 38 to Hsp90 and Cdc37. Subsequent tests revealed that the efficacy of compound 38 was associated with its interaction with Hsp90 and Cdc37, since its potency diminished in cells that overexpressed these proteins. The study examined the impact of compound 38 on apoptosis induction and tumour growth suppression, both *in vitro* and *in vivo*. Imidazoles connected to C-20 of celastrol can influence its Michael addition, potentially enhancing its anticancer efficacy. This discovery may have wider implications for the advancement of further Michael acceptors as antineoplastic medicines. Secondly, the results of the study emphasize the need to balance the role of covalent and noncovalent binding, and this is depicted through the activity of compound 38, which showed improved anticancer activity without any observed toxicity, thus indicating an improved pharmacological profile compared to the normal Michael acceptors (Fig. 7). The study emphasizes the potential of targeting the Hsp90-Cdc37 complex as a viable strategy for cancer treatment, thereby facilitating new opportunities for drug discovery in this domain.^39^

**Fig. 8:**
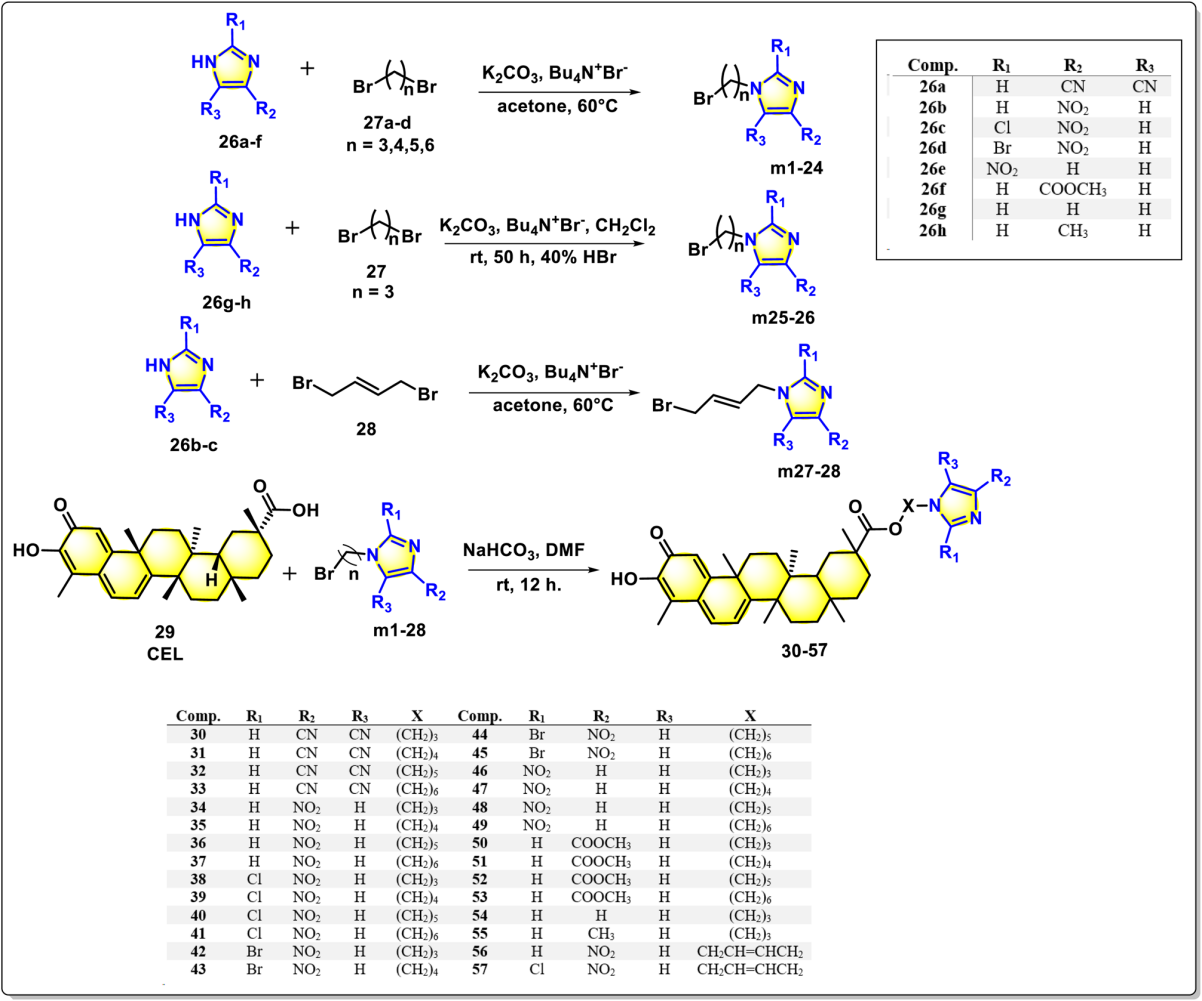
Synthetic route for celastrol–imidazole derivatives (30–57),^39^ reproduced from ref. 39 with permission from American Chemical Society, *J. Med. Chem.*, 2022, **65**, 4578–4589, Copyright 2022.

The celastrol-imidazole library (30–57) was synthesised through a multi-step sequence as outlined in Fig. 8. Initially, various imidazole derivatives (26a–f & 26b–c) were *N*-alkylated with dibromoalkanes using K_2_CO_3_ as a base and Bu_4_N^+^Br^−^ as a phase transfer catalyst in acetone 60 °C, yielding intermediates m1-24 and m27-28. For precursors 26g–h a modified alkylation with 1,3-dibromopropane was performed using Bu_4_N^+^Br^−^ in CH_2_Cl_2_ at ambient temperature for 50 hours. After completion of the reaction, the mixture was acidified with 40% HBr and washed with water to obtain intermediates m25 and m26 (Fig. 8). Finally, the target compounds 30–57 were prepared by coupling m1-m28 with the C-20 of CEL (29) in presence of sodium bicarbonate in DMF.^39^

### 2.5 Synthesis, design, and biological studies of uracil-azole derivatives as cytotoxic agents

**Fig. 9:**
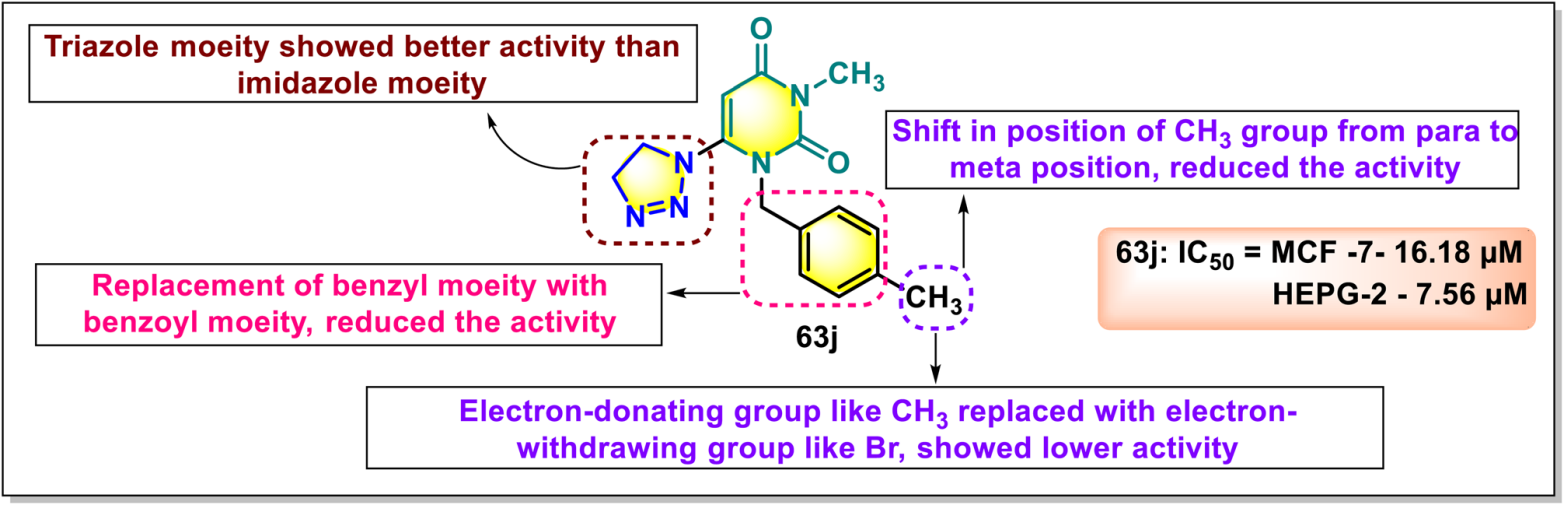
Structural features and SAR analysis of the most potent uracil–azole hybrid synthesised by Emami *et al.*^40^

The design and synthesis of innovative cytotoxic agents remain a compelling subject for medicinal chemistry researchers because of the adverse side effects associated with anticancer medications. The study by Emami *et al.* (2024) involved the design and synthesis of a unique family of uracil–azole hybrids. The cytotoxic activity and computational investigations including molecular docking, density functional theory, molecular dynamics simulation, and ADME features, was also examined. These compounds exhibited significant inhibitory efficacy against breast and hepatocellular cancer cell lines when compared to cisplatin as a positive control. Among these compounds, 63j exhibited the most favourable selectivity profile and demonstrated significant activity, with IC_50_ values of 16.18 µM and 7.56 µM against the MCF-7 and HepG-2 cell lines, respectively (Fig. 9). Furthermore, structure–activity relationships indicated that the differences in cytotoxic efficacy of the prepared compounds were influenced by different substitutions of the benzyl moiety while the docking results indicated that 63j binds effectively at the active site of Epidermal Growth Factor Receptor (EGFR), producing a stable complex by the EGFR protein. The results indicated that these uracil–azole hybrids may serve as potential cytotoxic agents.^40^

**Fig. 10:**
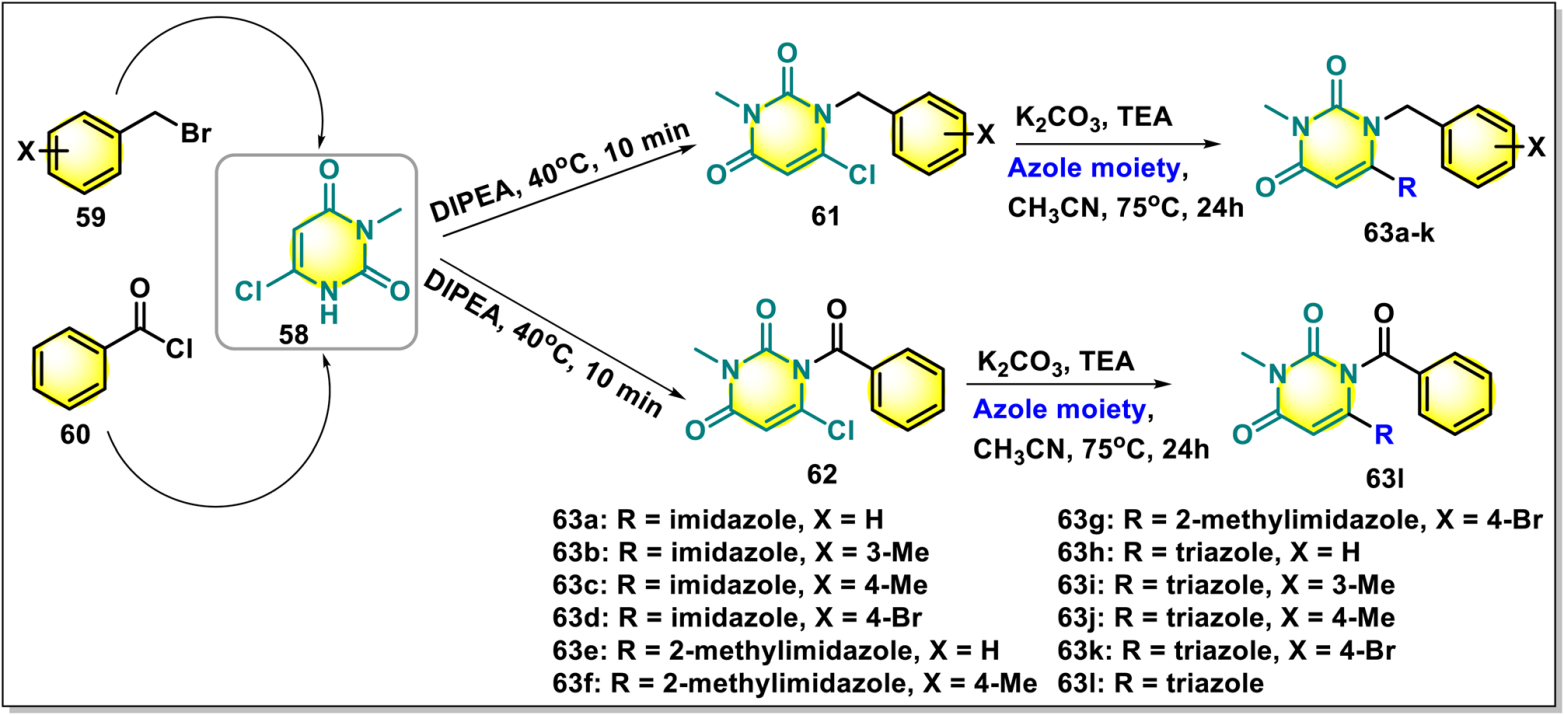
Synthetic pathway for the preparation of uracil–azole hybrids 63a–l,^40^ reproduced from ref. 40 under the terms of the Creative Commons Attribution 4.0 license (CC BY 4.0), Copyright 2024, L. Emami, F. Zare, S. Khabnadideh, Z. Rezaei, Z. Sabahi, S. Z. Gheshlaghi, M. Behrouz, M. Emami, Z. Ghobadi, S. M. Ardekani, F. Barzegar1, A. Ebrahimi, R. Sabet.

The uracil-azole hybrid compounds were synthesized using a two-step synthetic approach. In the first step, 3-methyl-6-chlorouracil (58) was treated with various benzyl bromides (59) or benzoyl chloride (60) to furnish the corresponding benzylated and benzoylated uracil intermediates 61 and 62, respectively. Subsequently, nucleophilic aromatic substitution of these intermediates with selected azole heterocycles, including imidazole, 2-methylimidazole, and 1,2,4-triazole, afforded the target uracil–azole derivatives 63a–l. The complete synthetic route is illustrated in Fig. 10.^40^

### 2.6 Design, synthesis and anticancer activity of sulphenylated imidazo-fused heterocycles

**Fig. 11:**
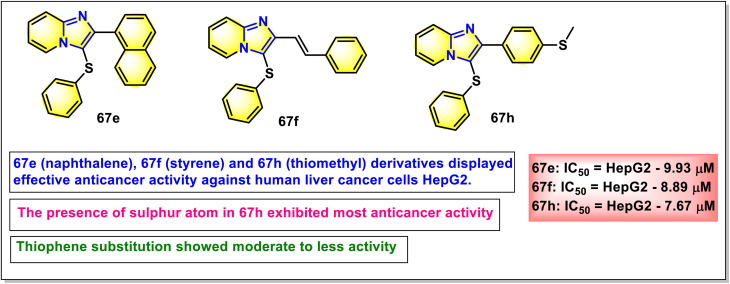
Structure–activity relationship and potency of lead imidazo[1,2-*a*]pyridine derivatives synthesized by Chitrakar *et al.*^41^

**Fig. 12:**
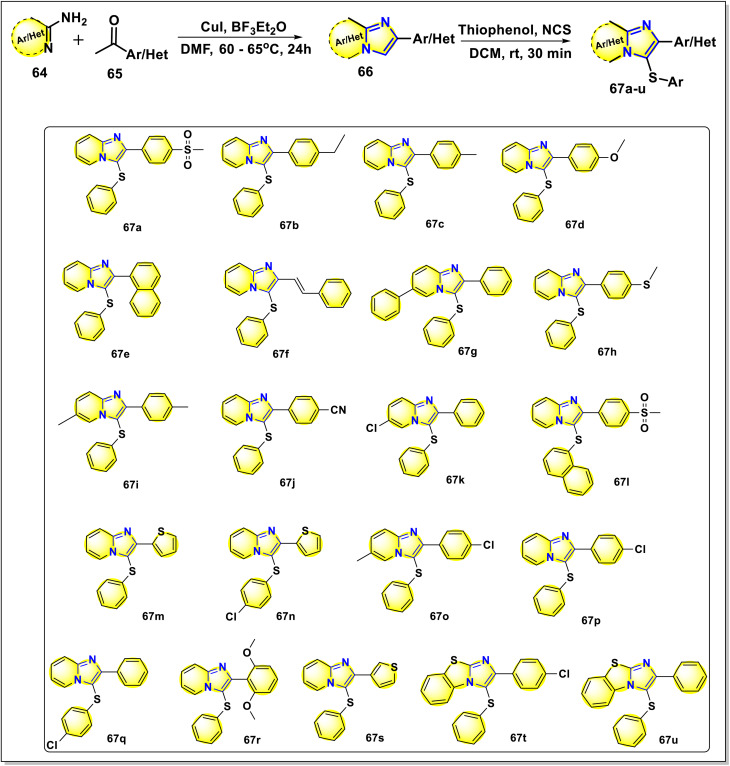
Synthesis of 3-sulphenylated imidazo[1,2-*a*]pyridine derivatives (67a–u),^41^ reproduced from ref. 41 with permission from Elsevier, *Bioorg. Med. Chem. Lett.*, 2021, **49**, 128307, Copyright 2021.

Incorporating thio-bridge linkages into heterocyclic frameworks has emerged as an effective medicinal chemistry approach to enhance anticancer efficacy and expand the selectivity across different cell lines. In 2021, Chitrakar *et al.*, devised a method for synthesizing and subsequently assessing the anticancer potential of sulphenylated 2-phenylimidazo[1,2-*a*]pyridines and their derivatives. Their research involved the preparation of twenty sulphenylated imidazo[1,2-*a*]pyridine derivatives through regioselective C–S bond formation, utilizing a diverse array of aromatic and styryl-based thiols, 67a–u (Fig. 12). The MTT assay demonstrated that minor structural alterations, such as adding a thio-bridge and varying the thio-substituent on the imidazo[1,2-*a*]pyridine framework, resulted in notable shifts in functional affinity. This led to a new mechanism for boosting anticancer activity across seven distinct human cancer cell lines: breast, liver, cervical, lung, glioblastoma, skin melanoma, and prostate. Compounds 67e, 67f, and 67h showed significant effects on HepG2 human liver cancer cells, with IC_50_ values of 9.93 µM, 8.89 µM, and 7.67 µM, respectively (Fig. 11). Cell cycle analysis indicated that these compounds induced apoptosis in HepG2 human liver cancer cells and caused cell cycle arrest at the G2/M phase.^41^

### 2.7 Synthesis and anticancer evaluation of novel sulphonamide-imidazole hybrid piperazines: *in vitro* and *in silico* studies

**Fig. 13:**
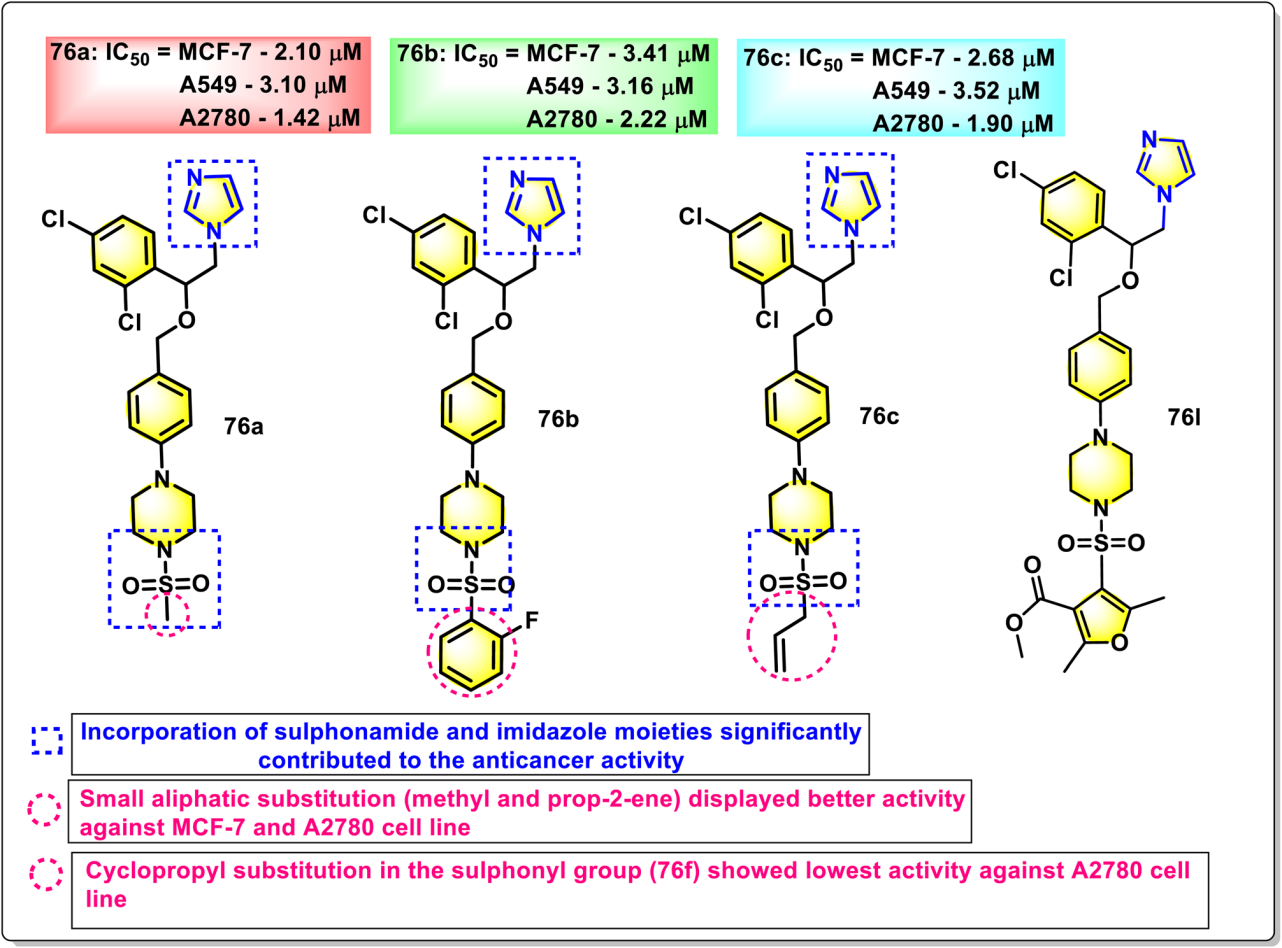
Chemical structures and antiproliferative activity of lead imidazole–sulphonamide derivatives (76a, 76b and 76c) and the toxicologically restricted derivative (76l) synthesised by S. R. Vootukoori *et al.*^42^

**Fig. 14:**
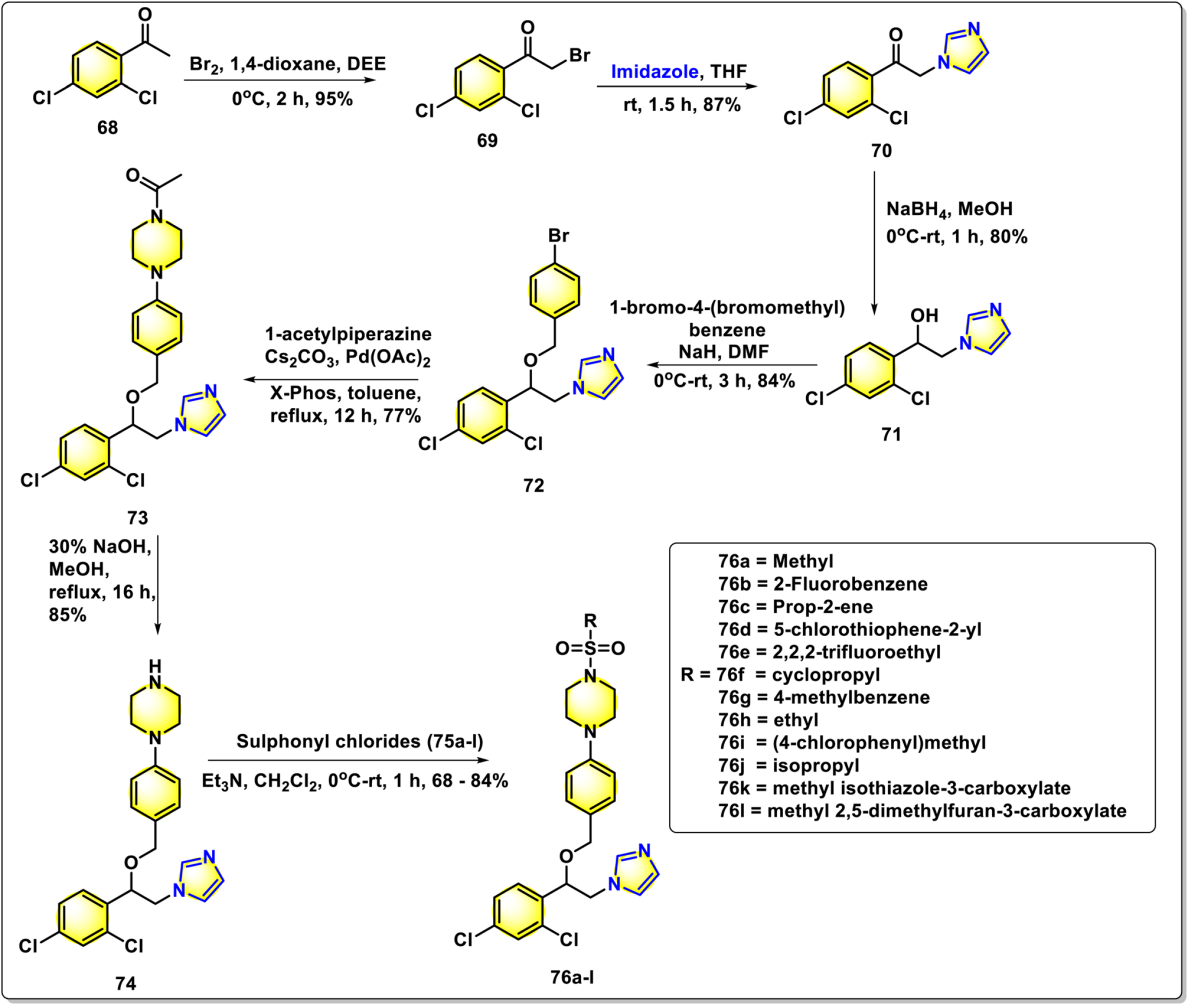
Synthetic scheme for sulphonamide-attached 1-(4-((1-(2,4-dichlorophenyl)-2-(1*H*-imidazol-1-yl)ethoxy)methyl)phenyl)piperazines derivatives,^42^ reproduced from ref. 42 with permission from John Wiley and Sons, *ChemistrySelect*, 2025, **10**, e202500011, Copyright 2025.

The sulphonamide and imidazole groups are each known for their significant anticancer effects, and their deliberate integration into a single piperazine-based structure offers a promising path for creating effective multitarget drugs. Given the significance, the synthesis and assessment of novel sulphonamide-imidazole hybrid piperazines as prospective anticancer drugs was reported by S. R. Vootukoori *et al.* (2025). A series of molecules (76a–l) was synthesised by integrating pharmacologically relevant imidazole and sulphonamide frameworks into a unified molecular scaffold. The target sulphonamide–imidazole hybrid piperazines (76a–l) were synthesized *via* a multistep sequence involving α-bromination, nucleophilic substitution with imidazole, reduction, and *O*-alkylation, followed by Pd-catalyzed C–N coupling to introduce the piperazine moiety (Fig. 14). Subsequent deprotection and final sulphonylation with various sulphonyl chlorides afforded the desired hybrid derivatives, enabling structural diversification at the terminal stage.^42^ The compounds were subsequently assessed for their anticancer efficacy against three human cancer cell lines: MCF-7 (breast), A549 (lung), and A2780 (ovarian). The *in vitro* cytotoxicity experiments demonstrated favourable outcomes, especially for compounds 76a, 76b, and 76c (Fig. 13). These compounds exhibited notable anticancer efficacy against MCF-7 cells, with IC_50_ values comparable to those of the reference compound, etoposide. Compounds 76a and 76b showed significant activity against A549 and A2780 cell lines, indicating broad-spectrum effectiveness. Molecular docking analysis were performed with human topoisomerase IIβ to elucidate the potential mechanism of action.

The docking studies demonstrated substantial hydrogen bonding and hydrophobic interactions, suggesting a viable mechanism for the compounds' anticancer efficacy. ADME and toxicity assessment of the produced compounds were performed and most derivatives exhibited advantageous drug-like properties; however, compound 76l was recognized as a potential reproductive toxicant. The structure–activity relationship analysis emphasized the influence of certain sulphonyl substitutions on cytotoxic efficacy. The integration of *in vitro* experiments with *in silico* investigations provides a thorough evaluation of the compound's viability as therapeutic candidates, reconciling efficacy with drug-like characteristics and potential toxicities.^42^

This study uniquely integrates two pharmacologically relevant scaffolds, imidazole and sulphonamide, into a single molecular framework.

### 2.8 Discovery of 3-(fluoro-imidazolyl)pyridazine derivatives as potent STING agonists with antitumor activity

**Fig. 15:**
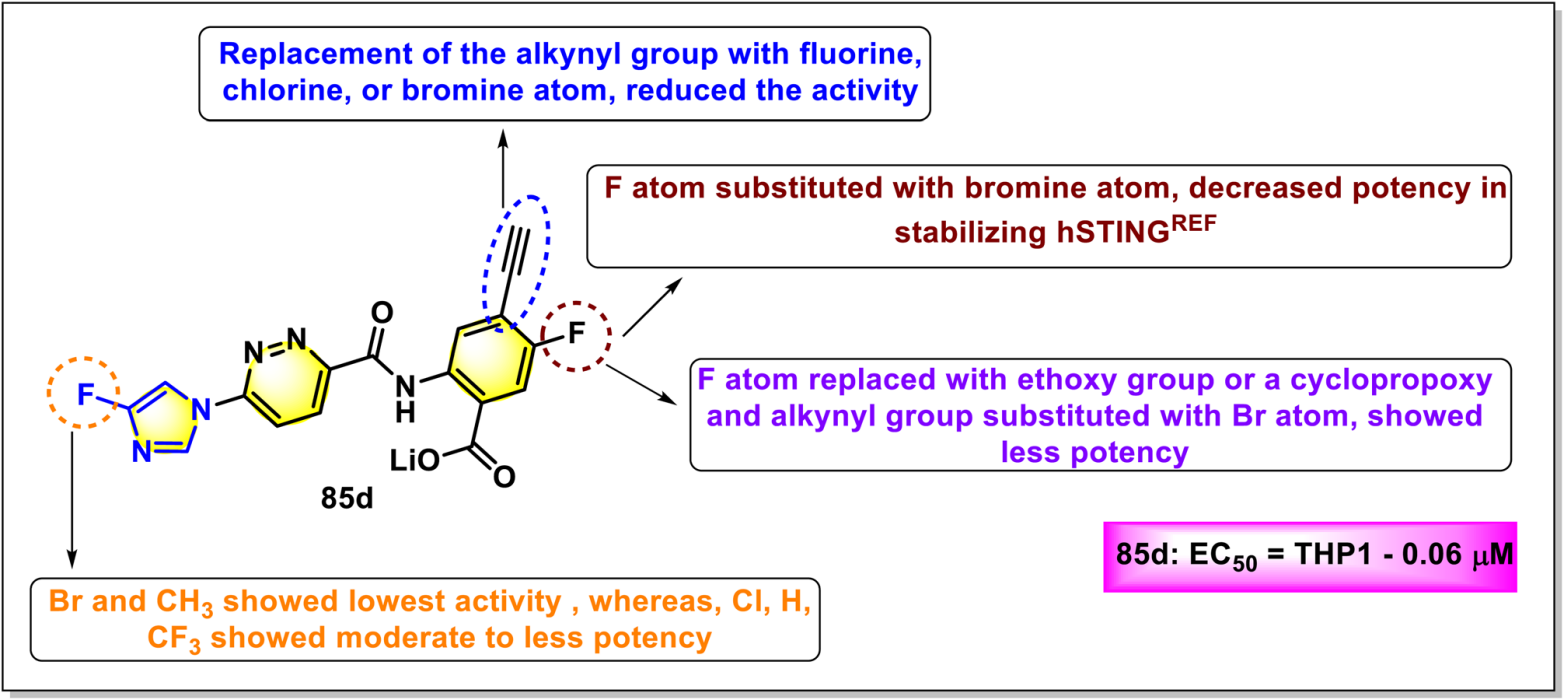
Structure and biological activity of the most potent compound 85d synthesised by Hou H. *et al.*^43^

Activating the STING pathway has become a promising approach in cancer immunotherapy. However, current agonists face challenges due to their limited effectiveness across various STING variants and inadequate antitumor activity in living organisms, highlighting the need for structurally innovative candidates. To overcome these challenges, Hou *et al.* (2025) developed and synthesized a series of 3-(fluoro-imidazolyl)pyridazine-based STING agonists using a convergent two-line synthetic method. This effort led to the creation of compound 85d, which exhibited remarkable binding affinity to both hSTING and mSTING variants, strong cellular activation, and superior tumour regression *in vivo* in the B16.F10 xenograft model when compared to the standard agonist SR-717. The efficacy of compound 85d in both *in vitro* and *in vivo* studies indicates its potential as a viable option for subsequent preclinical advancement in tumour immunotherapy (Fig. 15).^43^ This research advances the development of effective STING agonists for cancer treatment, addressing limitations of existing STING agonists and potentially providing new alternatives for patients with diverse tumour forms.

**Fig. 16:**
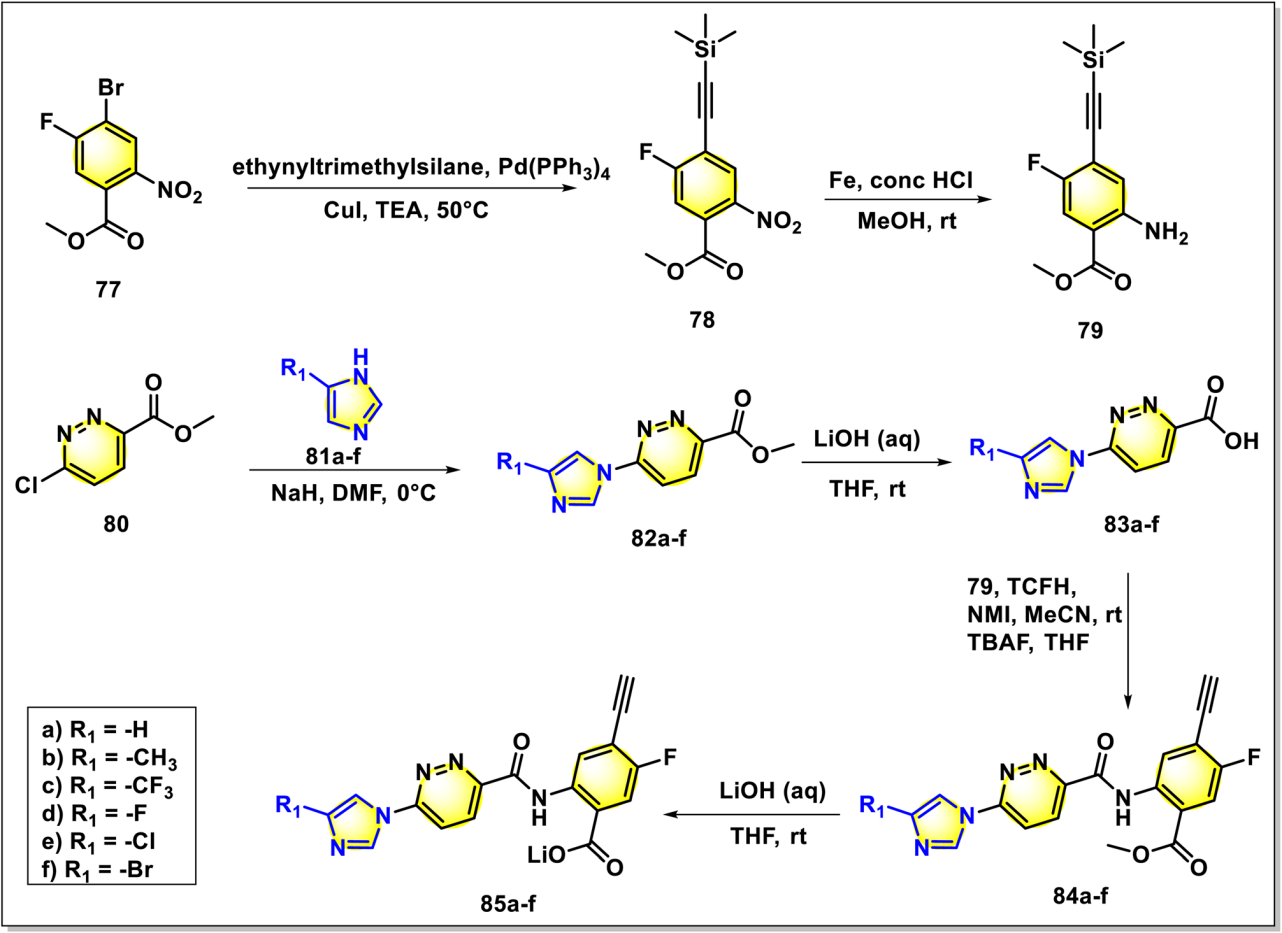
Synthetic scheme for the preparation of imidazole–pyridazine hybrids 85a–f,^43^ reproduced from ref. 43 with permission from American Chemical Society, *J. Med. Chem.* 2025, **68**, 9864−9885, Copyright 2025.

The production of compounds 85a–f is illustrated in Fig. 16. The Sonogashira reaction was employed between commercially available methyl 4-bromo-5-fluoro-2-nitrobenzoate (77) and ethynyltrimethylsilane for the formation of compound 78. The subsequent reduction of 78 using Fe and concentrated HCl yielded compound 79. Methyl 6-chloropyridazine-3-carboxylate (80) was reacted with various substituted imidazoles (81a–f) *via* nucleophilic aromatic substitution under alkaline conditions to yield compounds 82a–f, which were then converted to the corresponding carboxylic acids (83a–f) through ester hydrolysis using LiOH. The condensation of 83a–f with intermediate 79, followed by TBAF-mediated deprotection of the TMS group, yielded compounds 84a–f, which upon hydrolysis afforded the target compounds 85a–f.^43^

### 2.9 Structure and cytotoxic activity of novel 2-(coumarinyl-6-azo) imidazoles

Azo-heterocyclic hybrids have attracted considerable attention in the field of medicinal chemistry due to their structural adaptability and wide-ranging biological effects. Nonetheless, the strategic combination of coumarin pharmacophores with imidazole frameworks *via* an azo linkage is still not extensively explored as a method for synthesizing effective cytotoxic agents targeting macrophage-derived tumour cell lines. To address this gap, Datta *et al.* (2022) synthesized a series of new 2-(coumarinyl-6-azo)imidazole derivatives, 88–89 (Fig. 18). The synthesis of compounds involved *via* diazotization of 6-aminocoumarin followed by azo coupling with imidazole derivatives to form the key –N=N– linked framework. Subsequent *N*-alkylation of the imidazole nitrogen using alkyl iodides under basic conditions afforded the corresponding substituted derivatives. Two series of compounds were created: 1-methyl-2-(coumaryl-6-azo) and 2-(coumaryl-6-azo)-4-substituted imidazole (HCZ-R) and its methylated derivatives.^46^

**Fig. 17:**
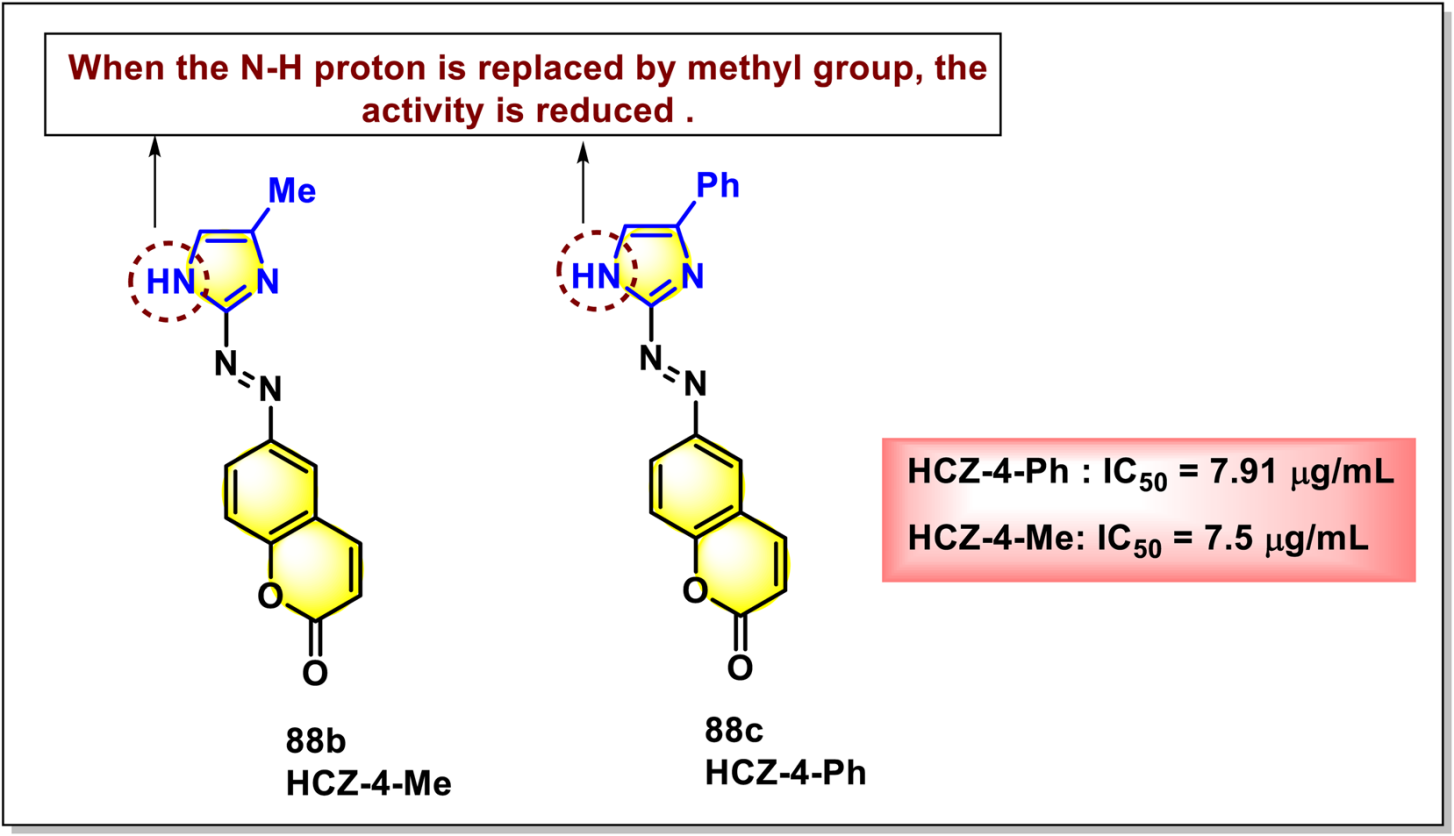
Structure and cytotoxicity data for compounds 88b and 88c designed by Datta *et al.*^46^

**Fig. 18:**
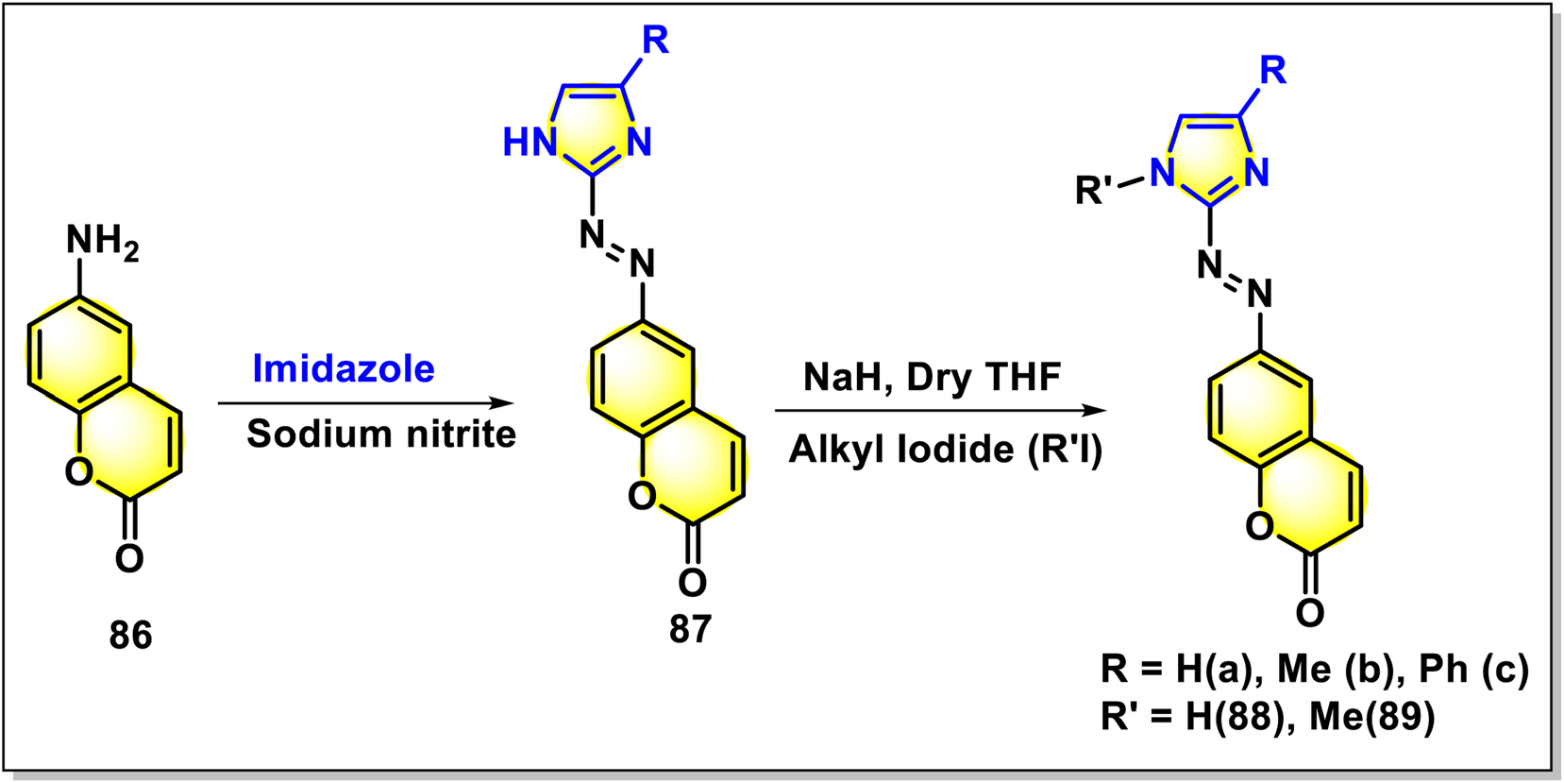
Synthetic pathway for the preparation of coumaryl-*azo*-imidazole derivatives 88a–c and 89a–c,^46^ reproduced from ref. 46 under the terms of the Creative Commons Attribution 4.0 license (CC BY 4.0), Copyright 2022, P. Datta, D. Sardar, C. Sinha, A. Manna and M. Chatterjee.

They confirmed through single-crystal X-ray diffraction that compound 88c adopts a *trans*-configuration around the –N=N– bond. Furthermore, they demonstrated that *N*-unsubstituted analogues (HCZ-R series), especially 88b and 88c, showed greater cytotoxicity compared to their *N*-methylated versions (R′CZ-R) in RAW 264.7 macrophage cells, highlighting the essential role of the free N–H group in facilitating biological activity (Fig. 17).^46^

### 2.10 Potential 2,4-dimethyl-1*H*-pyrrole-3-carboxamide bearing benzimidazole template: design, synthesis, *in vitro* anticancer and *in silico* ADME study

**Fig. 19:**
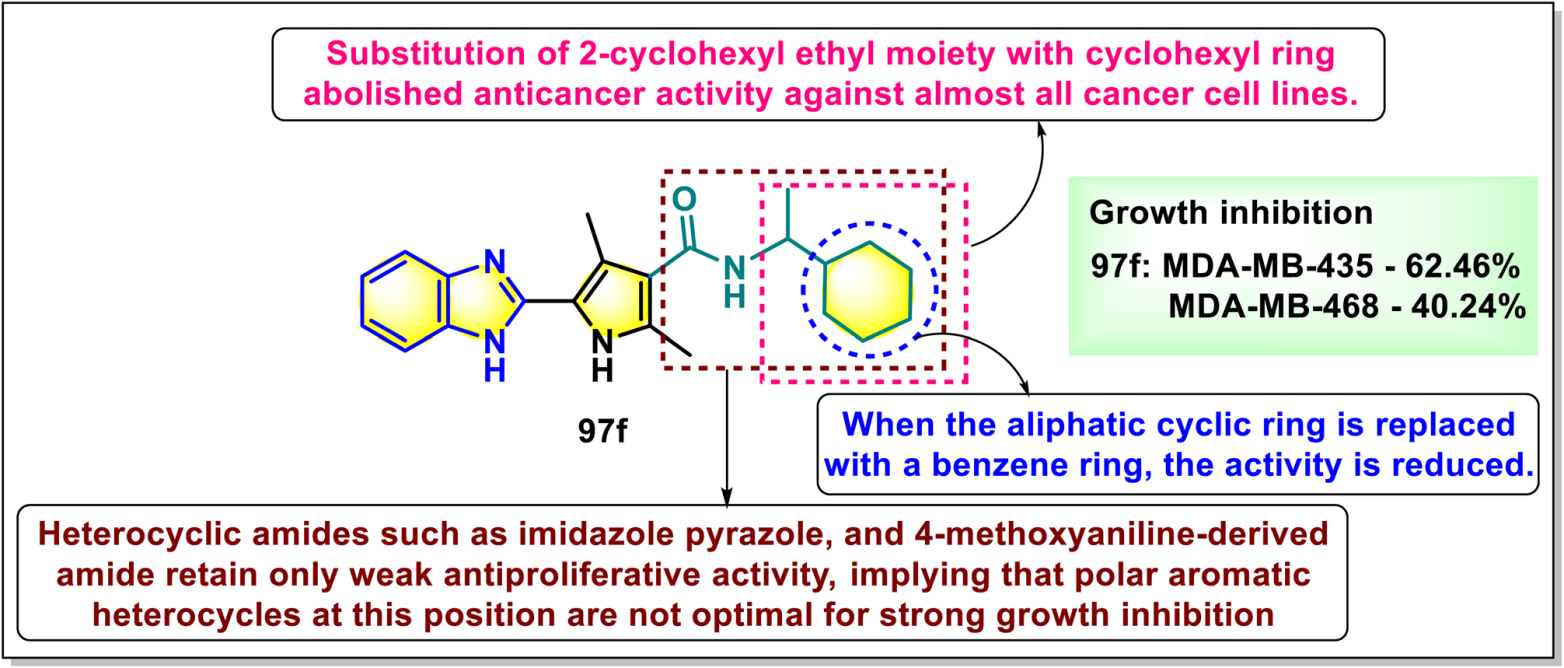
Structural features and growth inhibition profile of the most potent compound 97f synthesised by Rasal N. K. *et al.*^47^

Compounds containing benzimidazole have garnered considerable interest in the field of medicinal chemistry due to their varied pharmacological characteristics, including significant anticancer effects. Similarly, pyrrole-based carboxamides have shown promising cytotoxic effects against a range of tumour cell lines. By leveraging the complementary biological properties of these two frameworks, Rasal N. K. *et al.* (2020) prepared a series of innovative hybrid conjugates featuring a benzimidazole moiety designed to achieve improved and selective anticancer effectiveness. They then used the NCI-60 panel to evaluate the compounds' *in vitro* anticancer efficacy against leukaemia, melanoma, lung, colon, central nervous system, ovarian, renal, prostate, and breast cancer cell lines at a single concentration of 10 µM. Compound 5-(1*H*-benzo[*d*]imidazol-2-yl)-*N*-(1-cyclohexylethyl)-2,4-dimethyl-1*H*-pyrrole-3-carboxamide (97f) showed significant antiproliferative activity specifically against MDA-MB human cancer cell lines. Some derivatives of all the synthesized conjugates showed variable levels of efficacy, even at low dosages. Compound 97f significantly inhibited the growth of the MDA-MB-435 melanoma cell line and the MDA-MB-468 breast cell line (Fig. 19). Its noteworthy physicochemical, pharmacokinetic, and drug-likeness properties were confirmed by the computational ADME analysis, which also showed good expected oral bioavailability.^47^ As a result, the development of anticancer drugs would benefit from these new hybrid compounds.

**Fig. 20:**
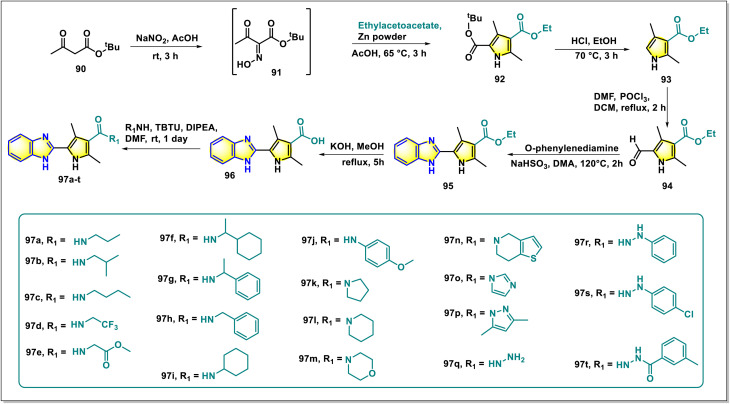
Synthetic scheme of pyrrole–benzimidazole derivatives 97a–t,^47^ reproduced from ref. 47 with permission from Elsevier, *Bioorg. Chem.*, 2020, **97**, 103660, Copyright 2020.

The target benzimidazole–pyrrole framework was constructed *via* a multistep sequence involving initial nitrosation and cyclization to form the heterocyclic core, followed by ester formation and functional group transformations (Fig. 20). The ester intermediate (95) was hydrolysed under basic conditions (KOH/MeOH) to afford the corresponding acid (96), and then finally series of amide derivatives 97a–t were prepared *via* coupling of acid intermediate (96) with various amines. This final step was achieved using TBTU-mediated amide coupling under mild conditions, enabling structural diversification through variation of amine substituents. NMR and mass spectrometry data validated the structures of the synthesized intermediates and compounds. The purity was assessed by HPLC prior to biological evaluation.^47^

## 3 Indoles

In medicinal chemistry, indole-based compounds are widely recognized as privileged scaffolds due to their broad pharmacological potential, particularly in the development of anticancer agents.^48,49^ Their inherent structural flexibility enables them to interact with diverse molecular targets central to cancer progression, including tubulin polymerization, DNA topoisomerases, and various protein kinases.^50^ Several clinically approved anticancer drugs underscore the importance of the indole nucleus: tyrosine kinase inhibitors such as sunitinib and osimertinib.^51^ Beyond these established drugs, recent efforts have focused on the rational design and synthesis of novel indole derivatives with enhanced bioactivity. For example, quinoline–indole hybrids have been reported to display significant cytotoxic activity by modulating apoptosis pathways, while Mo-catalysed cyclization strategies have yielded dihydroindolo–quinazolines with potent antiproliferative effects.^52^ Similarly, indole-linked pyrazoline and thiazolidinone scaffolds have demonstrated strong inhibition of topoisomerase II and promising antiproliferative activities, highlighting their potential as next-generation therapeutics.^53,54^ Recent advances in regioselective functionalization strategies, such as sulphenylation and triazole conjugation, have further expanded the chemical space of indole derivatives, offering improved selectivity and drug-like properties.^55,56^ Collectively, these developments illustrate the enduring role of indoles in anticancer drug discovery, where ongoing research continues to address challenges like multidrug resistance and therapeutic efficacy across diverse cancer models.

### 3.1 Design, synthesis, and anticancer activity studies of novel quinoline–indole derivatives

**Fig. 21:**
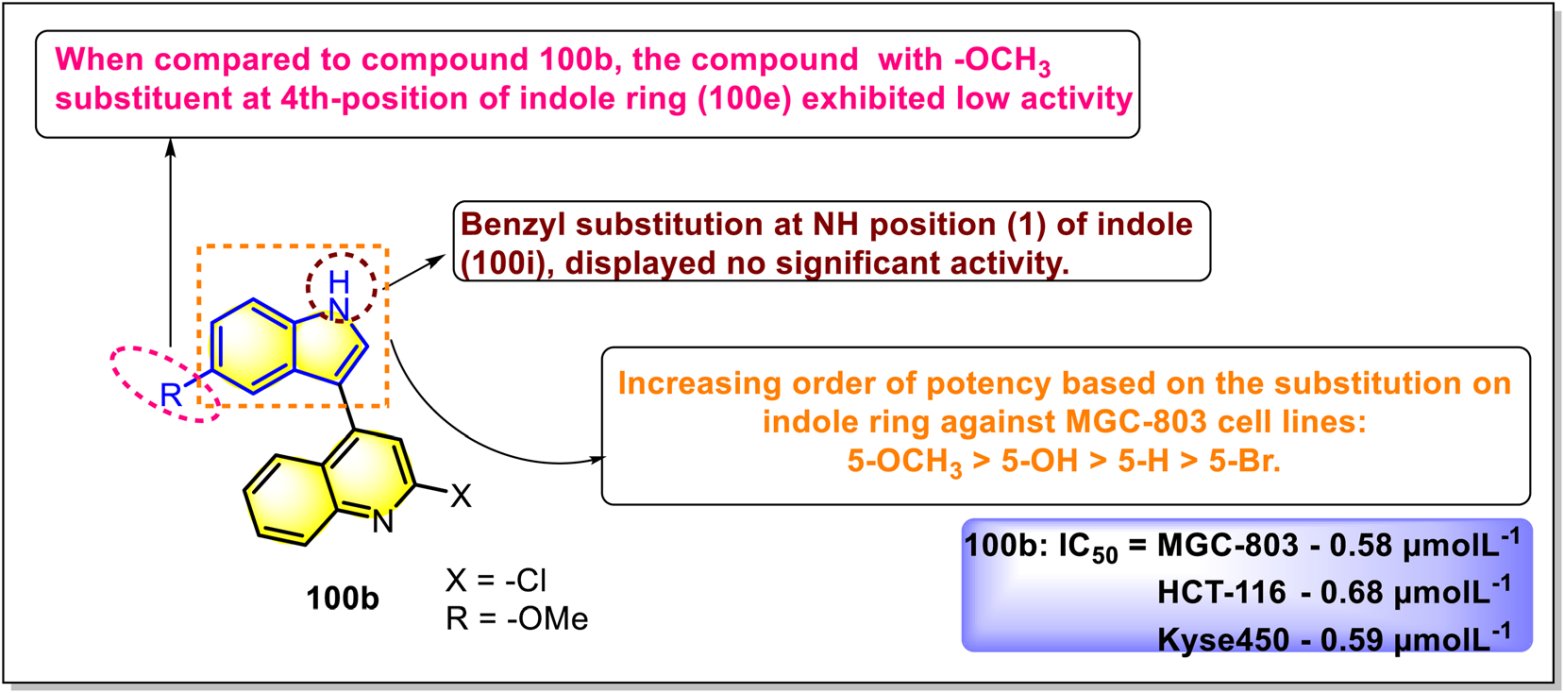
SAR analysis and potency of most potent compound 100b synthesised by Shenghui Wang *et al.*^57^

The exploration of molecular hybridization was advanced through the integration of quinoline and indole scaffolds to develop effective anticancer agents. Shenghui Wang *et al.* (2021) employed Lewis acid-catalysed coupling reactions to synthesize a series of novel quinoline–indole derivatives, which exhibited promising antiproliferative activity across various cancer cell lines. The MTT assay was used to evaluate the antiproliferative effectiveness of all targeted medications on the gastric cancer cell line MGC-803, the colon cancer cell line HCT-116, and the oesophageal cancer cell line Kyse450. 2-Chloro-4-(5-methoxy-1*H*-indol-3-yl)quinoline (100b) demonstrated strong inhibitory activity against MGC-803, Kyse450 and HCT-116 cell lines with IC_50_ values of 0.58 µmol L^−1^, 0.68 µmol L^−1^, and 0.59 µmol L^−1^, respectively (Fig. 21). Compound 100b inhibited the colony formation of MGC-803 and HGC-27 cells and also decreased the amounts of apoptosis-related proteins and induced intrinsic apoptosis. It simultaneously inhibited HGC-27 and MGC-803 cells at the G2/M phase. All of these results suggest that compound 100b may be a promising lead molecule for anticancer drugs.^57^

**Fig. 22:**
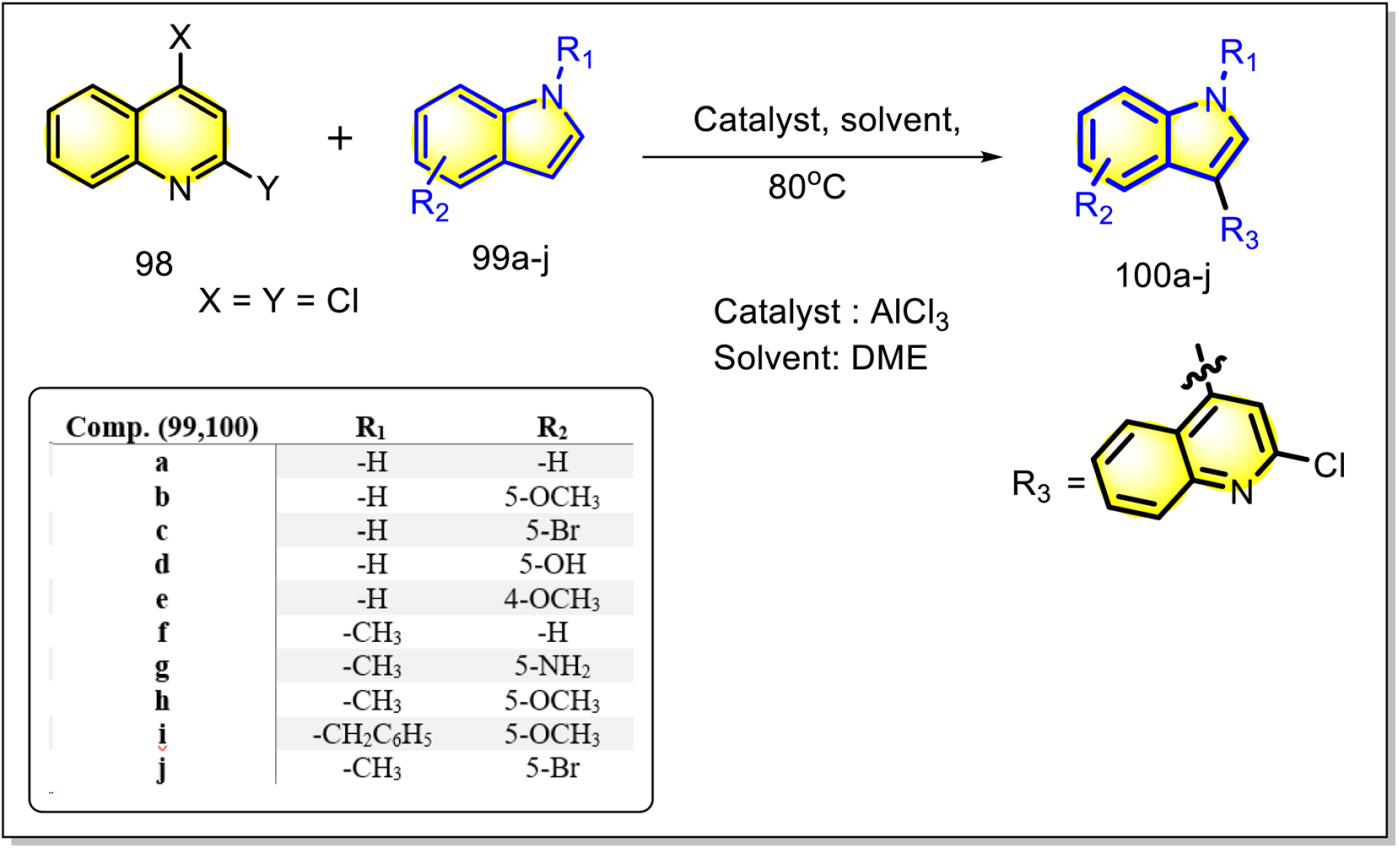
Synthesis of indole–quinoline derivatives 100a–j.^57^

The quinoline–indole hybrids were synthesised *via* a Lewis acid (AlCl_3_)-catalysed electrophilic substitution reaction between 2,4-dichloroquinoline (98) and substituted indoles (99a–j). This one-step coupling enables direct C–C bond formation at the indole C-3 position, facilitating efficient construction of the hybrid scaffold. Using commercially available 2,4-dichloroquinoline (98) and indoles (99a–j) as starting materials, all target compounds 100a–j were synthesized in a single step (Fig. 22).^57^

### 3.2 Mo-catalysed cyclization of *N*-vinylindoles and skatoles: synthesis of dihydroindolo[1,2-*c*]-quinazolines and dihydroindolo[3,2-*b*]-indoles, and evaluation of their anticancer activities

**Fig. 23:**
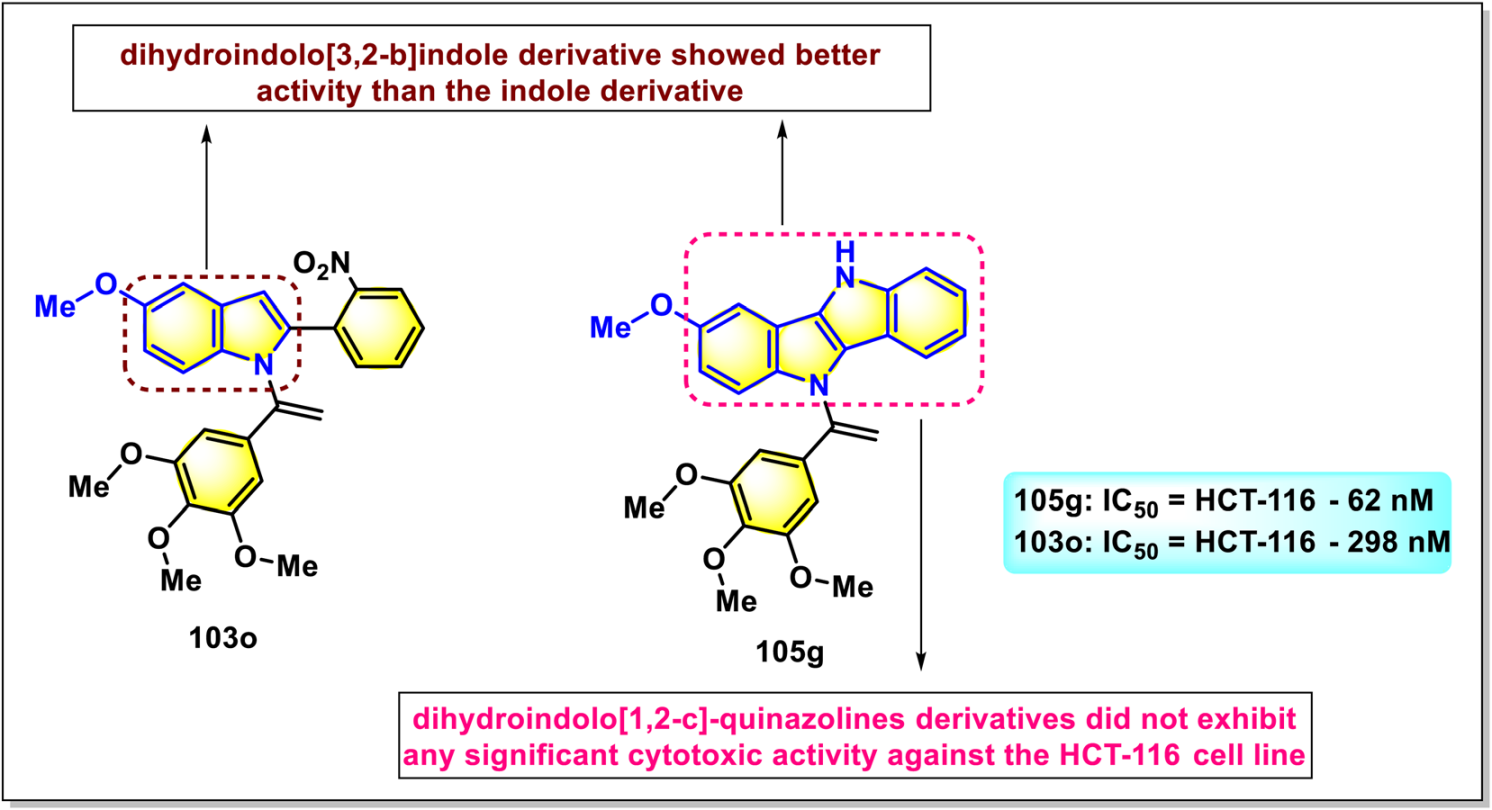
Structure and cytotoxic potency of most potent indole and dihydroindolo[3,2-*b*]indole derivatives synthesized by Pecnard S. *et al.*^52^

**Fig. 24:**
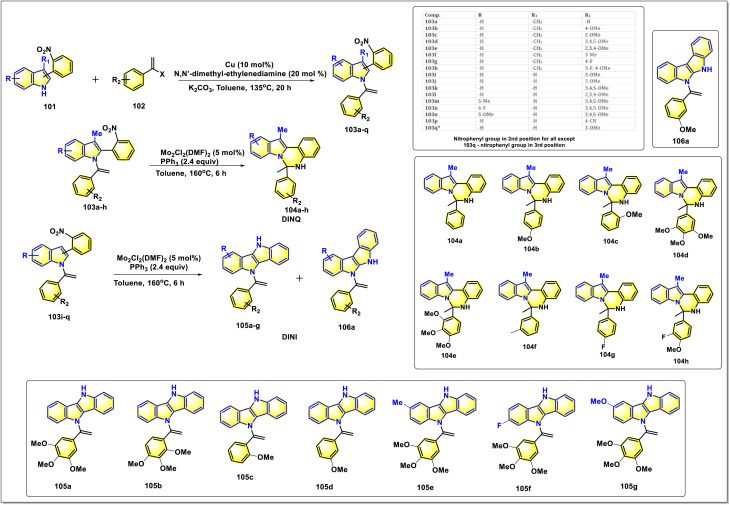
Divergent synthesis of DINQ and DINI scaffolds,^52^ reproduced from ref. 52 under the terms of the Creative Commons Attribution 3.0 license (CC BY 3.0), Copyright 2024, S. Pecnard, X. Liu, O. Provot, P. Retailleau, C. Tran and A. Hamze.

Following the encouraging anticancer potential demonstrated by nitrogen-rich heterocyclic hybrids, researchers have also focused on creating more intricate polycyclic indole-fused structures using novel catalytic techniques. In this context, Pecnard S. *et al.* (2024) utilized a Mo-catalyzed cyclization approach to develop two unique categories of indole-based hybrid compounds. These compounds exhibited significant cytotoxic effects, which were heavily influenced by particular structural elements like methoxy and trimethoxyphenyl groups. The employed strategy delivered unexpected dihydroindolo[1,2-*c*]-quinazolines (DINQ) and dihydroindolo[3,2-*b*]indole (DINI) compounds using *N*-vinylazoles as precursors. The synthesis proceeds *via* a Mo(CO)_6_/PPh_3_-catalysed cadogan-type reductive cyclization of *N*-vinylazoles, generating a nitrene intermediate that undergoes intramolecular cyclization to furnish the heterocyclic core, 105a–g (Fig. 24). This process enables divergent reactivity, leading to either dihydroindolo[1,2-*c*]quinazolines (104) or dihydroindolo[3,2-*b*]indoles (105) depending on the substitution pattern of the indole substrate. The mechanism involves the formation of an aniline intermediate followed by intramolecular cyclization in the presence of a Mo catalyst. This cyclization proceeds *via* nucleophilic aniline attack on the terminal double bond of the *N*-vinylazole substrate, yielding compound 104. However, when *N*-vinylazoles possessing a hydrogen atom at the C-3 position of the indole were utilized, the indole's double bond reacted preferentially over the terminal double bond of the *N*-vinylindole. This preference resulted in the entrapment of the nitrene intermediate and the synthesis of dihydroindolo[3,2-*b*]indole derivatives (105).^52^ All the compounds were tested against the HCT-116 human colon cancer cell line. Two compounds, 103o and 105g showed significant cytotoxicity (Fig. 23). 105g showing significantly higher activity (93% cytotoxicity) than 103o (66%). With an IC_50_ of 62 nM, compound 105g demonstrated potent antiproliferative activity, whereas compound 103o had an IC_50_ of 298 nM. This cytotoxic action requires the presence of a methoxy group and the 3,4,5-trimethoxyphenyl moiety on the indole or dihydroindolo[3,2-*b*]indole scaffold.^52^

### 3.3 Synthesis and biological evaluation of novel pyrazoline derivatives containing indole skeleton as anti-cancer agents targeting topoisomerase II

**Fig. 25:**
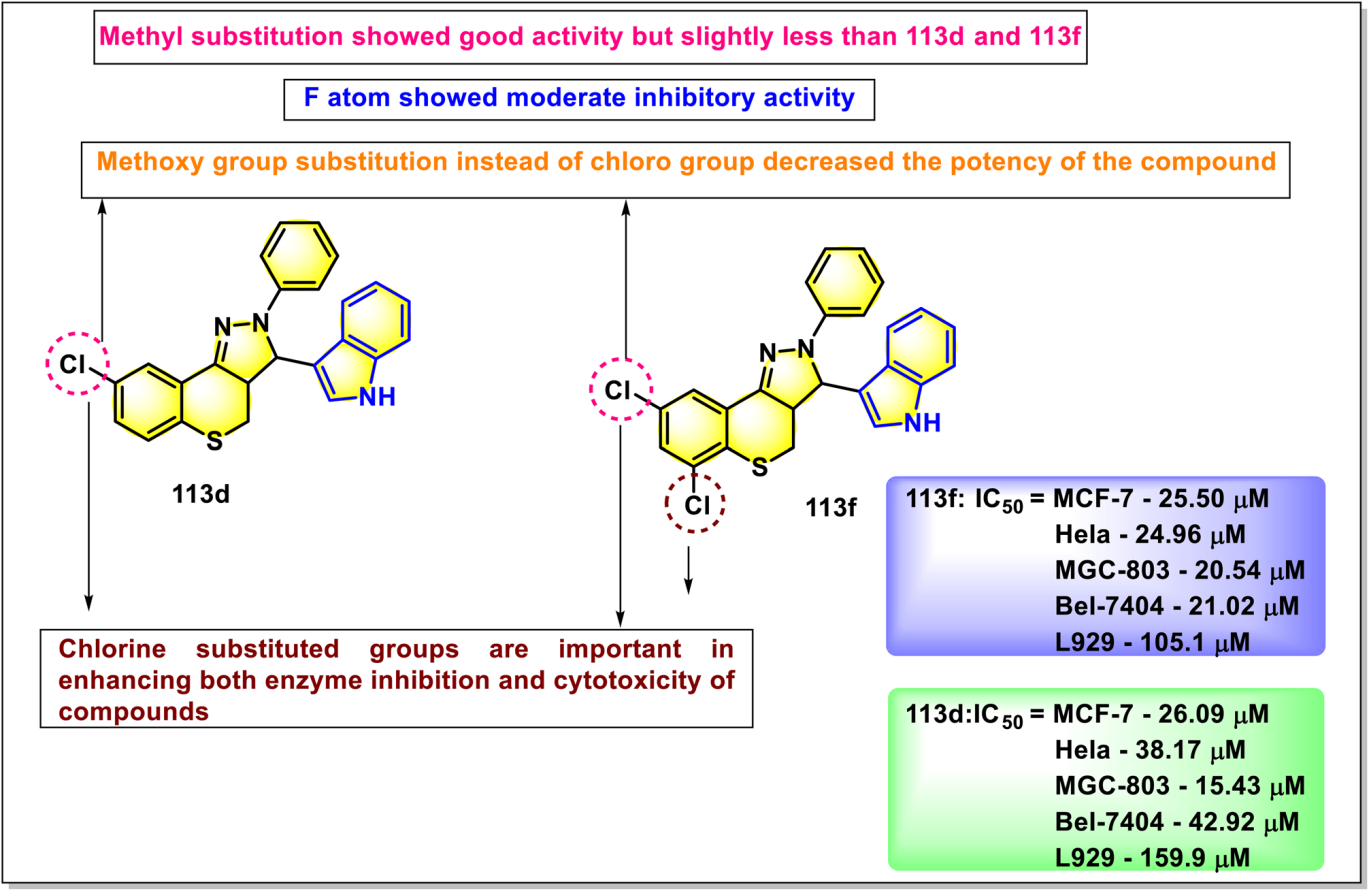
Structure and antiproliferative activity of the most potent compounds synthesized by Song *et al.*^53^

In a complementary approach targeting a specific cellular mechanism, Song *et al.* (2020) worked on the design and synthesis of novel indole–pyrazoline hybrid derivatives as selective topoisomerase II inhibitors for anticancer drug development. Utilizing a multistep synthetic sequence involving aldol condensation and hydrazone cyclization, a series of structurally diverse compounds were constructed and evaluated against four human cancer cell lines (MGC-803, HeLa, MCF-7, and Bel-7404) and the normal cell line L929. Most of the synthesized compounds demonstrated potential anticancer activity *in vitro* and exhibited decreased cytotoxicity against normal cells compared to positive controls. The study concentrated on the most promising compounds, 113d and 113f, which demonstrated potent antiproliferative activity. These compounds were found to induce cell cycle arrest at the G2/M phase and apoptosis in MGC-803 cells. The researchers employed cleavage reaction assays, DNA unwinding assays, and assays for the inhibition of DNA topoisomerase I and II to determine the mechanism of action. The results showed that most of the compounds exhibited selective inhibition of Topoisomerase II, whereas compound 113f was a nonintercalative Topo II catalytic inhibitor. The researchers found that the compounds’ cytotoxicity and enzyme inhibition were enhanced by the addition of chlorine-substituted groups. In addition, the study demonstrated that compounds 113d and 113f could easily enter the nucleus of the cancer cells, which is crucial to their mode of action (Fig. 25). A better understanding of the antiproliferative properties was gained through molecular docking studies. This research opens up new possibilities for developing targeted cancer treatments that may have fewer adverse effects on healthy cells.^53^

**Fig. 26:**
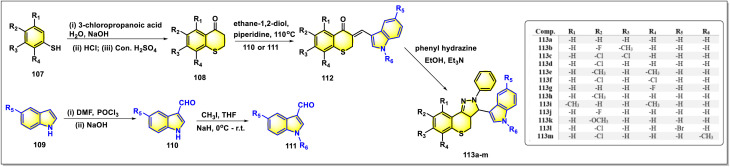
Convergent synthesis of indole–pyrazoline derivatives 113a–m,^53^ reproduced from ref. 53 with permission from Elsevier, *Eur. J. Med. Chem.*, 2020, **200**, 112459, Copyright 2020.

The synthetic pathway for the target compounds 113a–m is illustrated in Fig. 26. Thiochroman-4-one (108) was synthesised from benzenethiol (107) *via* acid-catalysed cyclisation using β-chloropropionic acid under dehydrating conditions. 1*H*-Indole-3-carbaldehyde (110) was synthesized from indole as the starting material (109) using the Vilsmeier–Haack reaction, followed by methylation to provide *N*-methyl-1*H*-indole-3-carbaldehyde (111). Compound 112 was synthesized from thiochroman-4-one (108) and either compound 110 or 111 through aldol condensation. Treatment of 112 with phenyl hydrazine yielded the target compounds 113a–m in the presence of triethylamine.^53^

### 3.4 Bimetallic iron–palladium catalyst system as a lewis-acid for the synthesis of novel pharmacophores based on indole scaffold as anticancer agents

**Fig. 27:**
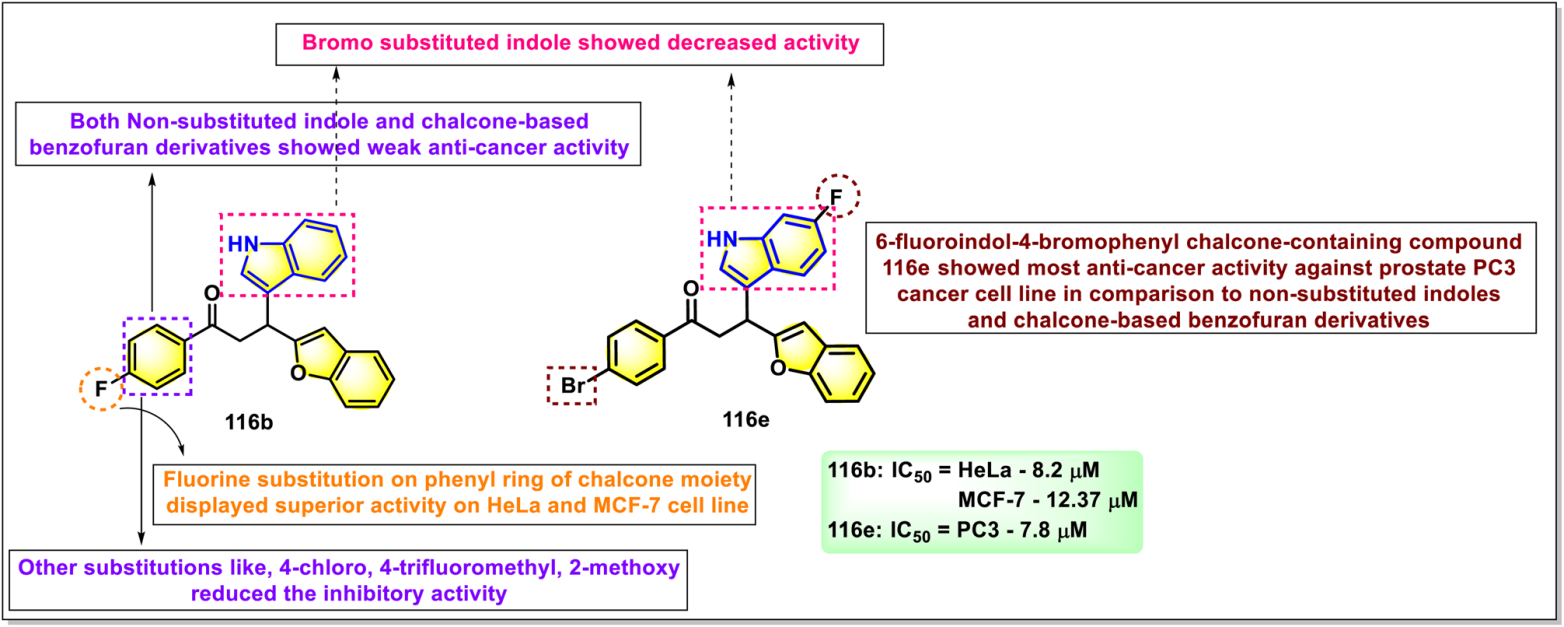
Structure–activity relationship (SAR) and cytotoxic efficacy of the most potent indole–chalcone hybrids synthesised by Islam M. S. *et al.*^58^

In an effort to further expand the structural diversity of indole-based anticancer hybrids, Islam M. S. *et al.* (2021) developed a bimetallic FeCl_3_/PdCl_2_-catalyzed Friedel–Crafts alkylation technique. This method was used to generate new bis-heteroaryl conjugates that integrate indole and chalcone units originating from benzofuran and benzothiophene structures. The synthesized compounds (116a–s) were evaluated for cytotoxicity against human fibroblast BJ cell lines and were determined to be either non-cytotoxic or minimally toxic. The anticancer effectiveness of these compounds was subsequently assessed against breast cancer (MCF-7), prostate cancer (PC3), and cervical cancer (HeLa) cell lines. Compared to the standard medication doxorubicin, the findings indicated moderate to weak anticancer effectiveness. Compound 116b, including a fluoro-substituted chalcone moiety, exhibited the highest efficacy against HeLa (IC_50_ = 8.2 µM) and MCF-7 (IC_50_ = 12.37 µM) cell lines. Compound 116e, including a 6-fluoroindol-4-bromophenyl chalcone structure, had the highest potency against the PC3 prostate cancer cell line (IC_50_ = 7.8 µM) (Fig. 27). The structures of the Friedel–Crafts adducts were confirmed using ^1^H, ^13^C NMR, Mass, and FT-IR spectroscopic data.^58^

**Fig. 28:**
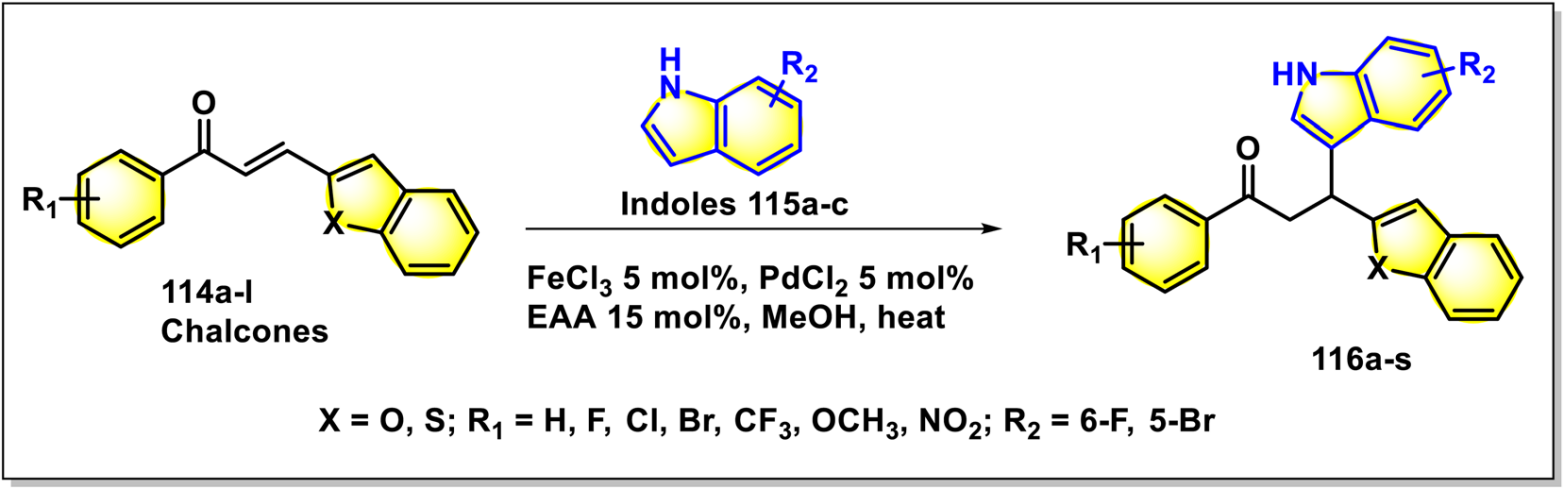
Iron/palladium-catalysed synthesis of indole–chalcone hybrids 116a–s,^58^ reproduced from ref. 58 under the terms of the Creative Commons Attribution 4.0 license (CC BY 4.0), Copyright 2021, M. S. Islam, M. Ali, A. M. Al-Majid, A. S. Alamary, S. Alshahrani, S. Yousuf, M. I. Choudhary, A. Barakat.

This reaction involved substituted indoles (115a–c) and chalcone derivatives (114a–k), proceeding through electrophilic activation of the enone moiety, followed by C-3 substitution of the indole. A variety of heteroaryl enone substituents exhibited a wide substrate scope. Both electron-deficient and electron-rich heterocycles produced the products, 116a–s in good yields (Fig. 28).^58^

### 3.5 Regioselective sulphenylation of indoles using sulphonyl hydrazides: synthesis and evaluation of anticancer activity

In addition to strategies for forming carbon–carbon bonds, incorporating sulphur-containing groups into the indole framework has been acknowledged as an effective method for improving the biological and pharmacological characteristics of indole-based anticancer drugs. In this regard, Yadav A. *et al.* (2025) devised a successful KSCN-mediated regioselective sulphenylation technique to generate new indole thioether compounds (Fig. 30). These compounds exhibited significant anticancer potential, supported by favourable molecular docking interactions and adherence to Lipinski's drug-likeness criteria. Diverse computational and experimental methodologies like DFT computations, Hirshfeld surface analysis, ADMET investigations, molecular docking, and *in vitro* cytotoxicity experiments were conducted to examine the characteristics and prospective applications of the produced substances. These compounds demonstrated strong interactions with key amino acid residues, including Lys482 (3.47 Å), Gln529 (3.28 Å), Thr528 (2.85 Å), Phe467 (2.85 Å), Gly95 (2.86 Å), Thr598 (3.30 Å), His538 (3.37 Å), Ser535 (3.22 Å), and Asn579 (3.43 Å), suggesting their potential as anticancer agents.^55^

**Fig. 29:**
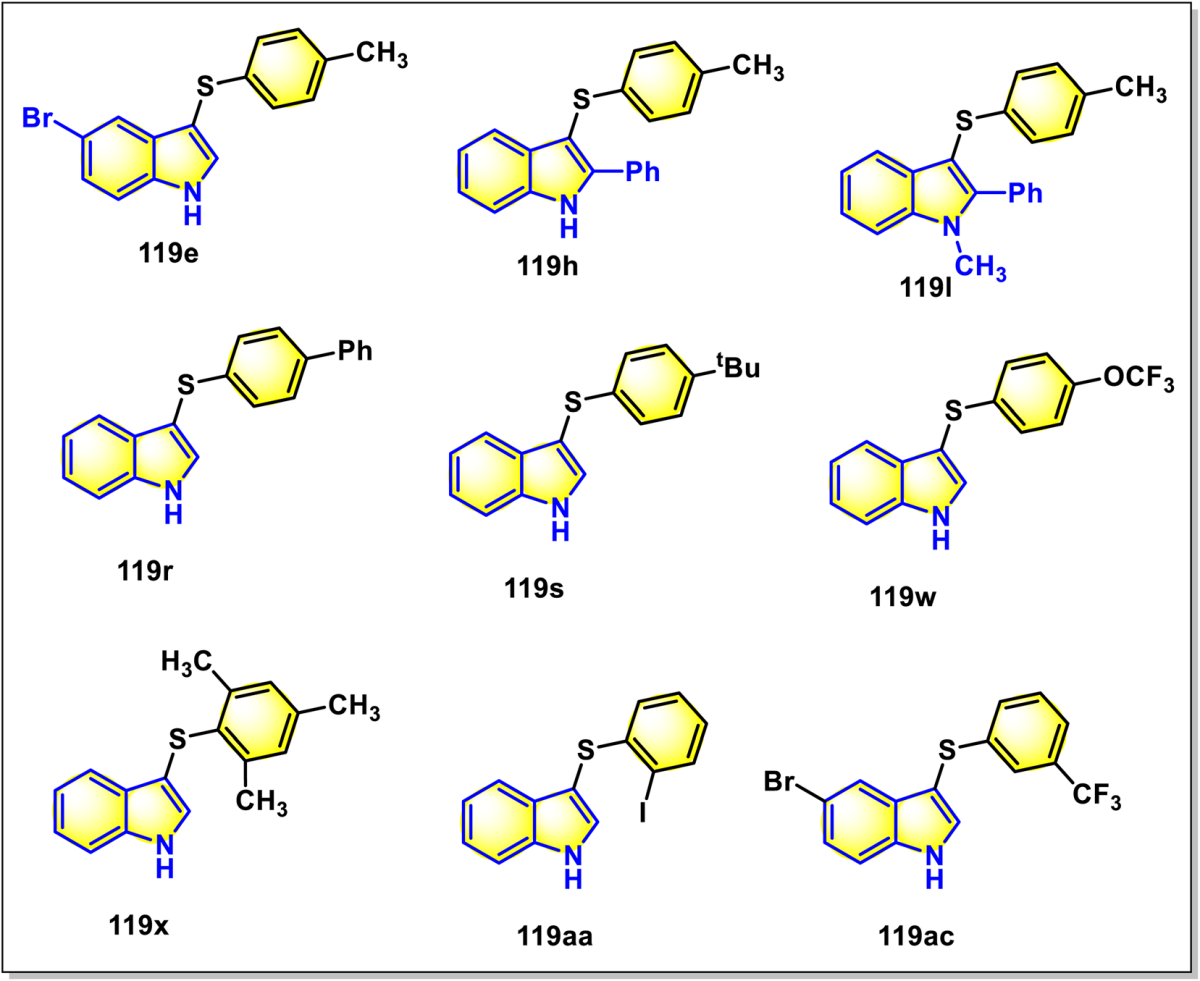
Indole thioether compounds synthesized by Yadav A. *et al.* that deviates from Lipinski's rule.^55^

**Fig. 30:**
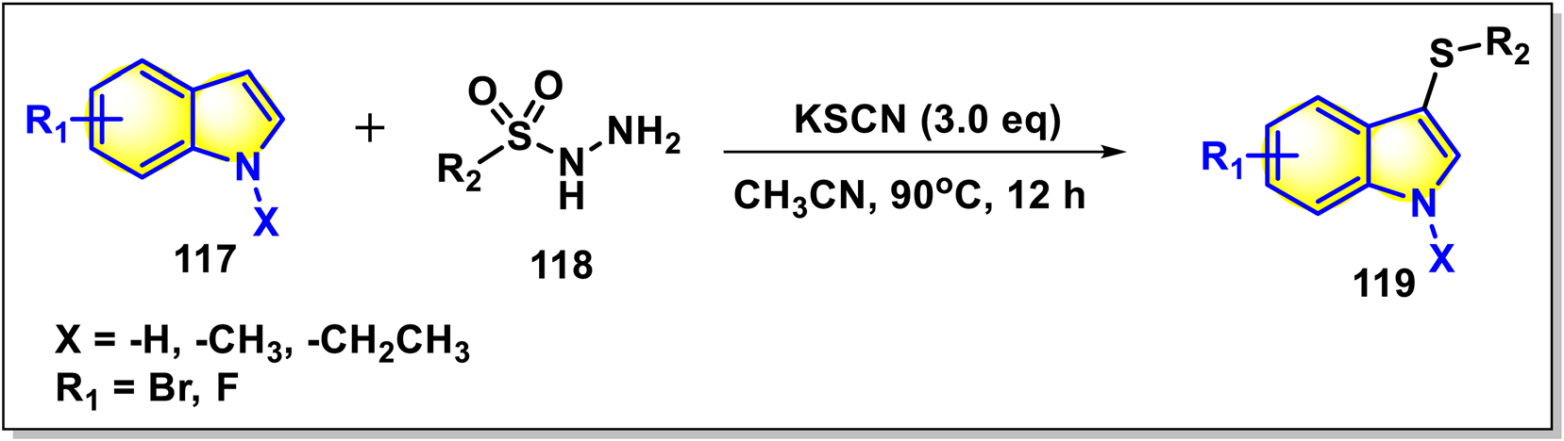
Regioselective sulphenylation of indoles using sulphonyl hydrazides,^55^ reproduced from ref. 55 with permission from Elsevier, *J. Mol. Struct.*, 2025, **1329**, 141346, Copyright 2025.

The indole thioether derivatives were synthesised *via* KSCN-mediated regioselective sulphenylation at the C-3 position of indoles (117) using sulphonyl hydrazides (118) under thermal conditions (CH_3_CN, 90 °C), leading to the formation of C–S bonded products (119) (Fig. 30). All the synthesized compounds except 119e, 119h, 119l, 119r, 119s, 119w, 119x, 119aa, and 119ac, comply with Lipinski's rule with at most one violation (Fig. 29). Consequently, these molecules may demonstrate pharmacological characteristics. A diminished lipophilicity value (LogP) indicates that the compounds can readily traverse the cell membrane, while a reduced total surface area (TPSA) facilitates efficient cell internalization, which is essential for drug-like characteristics. Drug-like qualities were subsequently assessed using bioactivity scores for ion channel modulators, G-protein-coupled receptor (GPCR) ligands, nuclear receptor ligands, and kinase inhibitors.^55^

### 3.6 New benzothiazole-indole-1,2,3-triazole-*N*-phenylacetamide derivatives as cytotoxic agents: design, synthesis, and *in vitro* cytotoxic evaluations

Recognizing the proven anticancer capabilities of benzothiazole, indole, 1,2,3-triazole, and *N*-phenylacetamide moieties when used as separate frameworks, it was proposed to combine them into a single hybrid structure. This approach aimed to create compounds with increased effectiveness and potentially new mechanisms of cytotoxic action. To achieve this design, Taherkhani A. M. *et al.* (2025) planned a multistep synthetic process utilizing well-known chemical transformations to facilitate the efficient and modular construction of the desired molecules. Thirteen new compounds (128a–m) were synthesized through a sequence of multistep transformations, starting with indole and incorporating several pharmacophores recognized for their anticancer efficacy.

**Fig. 31:**
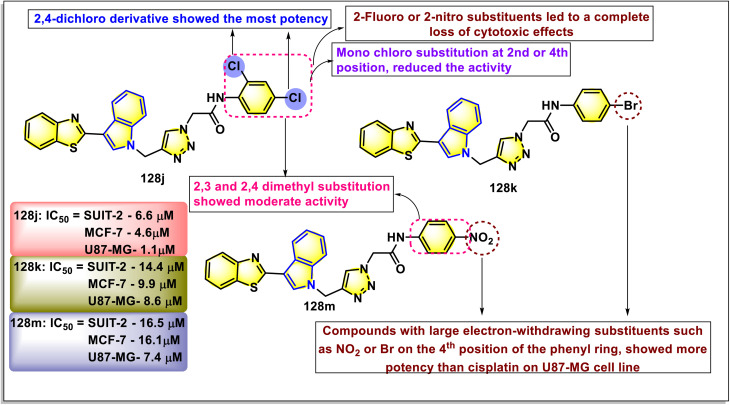
Inhibitory potency and SAR analysis of the most potent benzothiazole–indole hybrid derivatives synthesized by Taherkhani A. M. *et al.*^56^

The cytotoxic effects of these compounds were evaluated against three cancer cell lines such as SUIT-2 (pancreatic cancer), MCF-7 (breast cancer), and U87-MG (glioblastoma). The majority of the produced compounds exhibited cytotoxic effects on the evaluated cell lines, with compound 128j identified as the most effective. Significantly, 128j exhibited 1.8- and 15.5-times greater efficacy than cisplatin against MCF-7 and U87-MG cells, respectively. Along with it, 128m and 128k also showed greater efficiency than cisplatin in U87-MG cells (Fig. 31). A molecular docking analysis indicated that drug 128j may engage with topoisomerase II alpha, potentially elucidating its lethal mechanisms.^56^

**Fig. 32:**
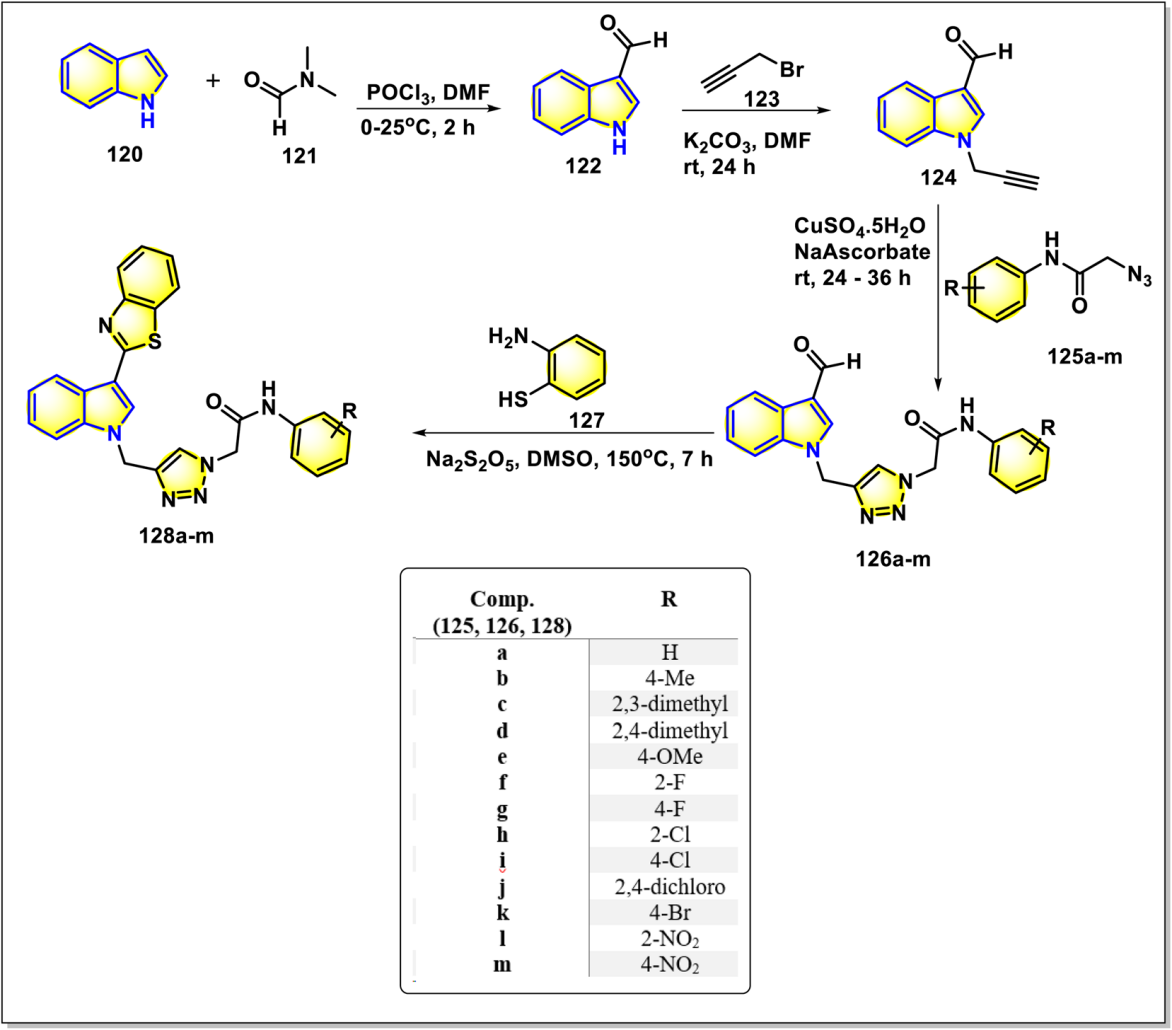
Synthesis of benzothiazole-indole-1,2,3-triazole-*N*-phenylacetamide derivatives,^56^ reproduced from ref. 56 with permission from Elsevier, *J. Mol. Struct.*, 2025, **1336**, 142089, Copyright 2025.

The target hybrids were synthesised *via* a sequential strategy involving Vilsmeier–Haack formylation of indole, followed by propargylation to generate alkyne intermediate 124, which subsequently underwent Cu(i)-catalysed azide–alkyne cycloaddition (CuAAC) to afford 1,2,3-triazole intermediates (125a–m). Final coupling with substituted thiophenol derivatives (127) under oxidative conditions furnished the desired benzothiazole-linked products (128a–m), enabling modular incorporation of multiple pharmacophores (Fig. 32).^56^

### 3.7 Synthesis and anticancer activity of indole-functionalized derivatives of betulin

**Fig. 33:**
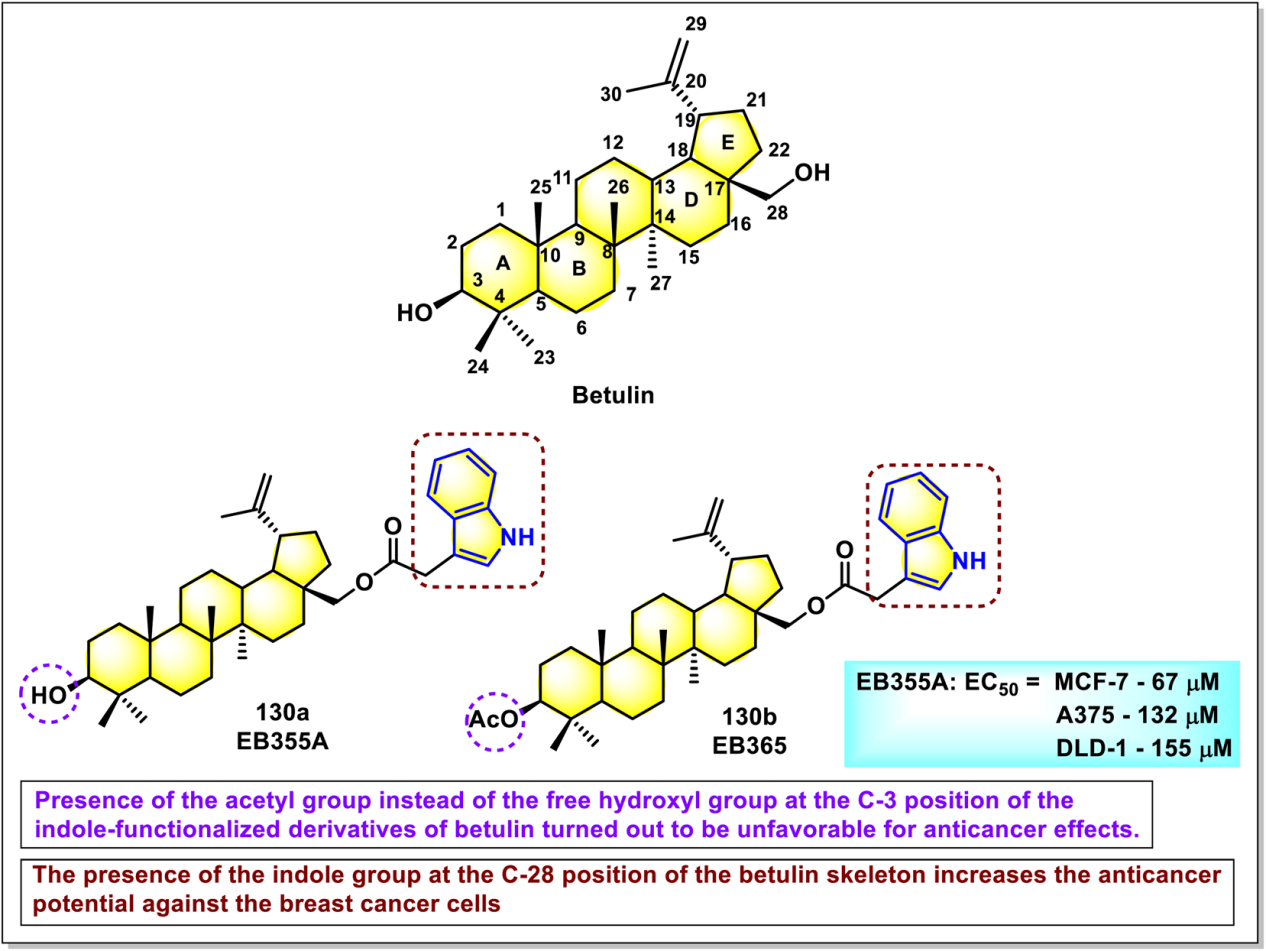
Structure and anticancer activity of the indole-functionalized betulin derivatives.^59^

The strategic modification of naturally sourced pentacyclic triterpenes by incorporating synthetic pharmacophores offers a promising path for creating hybrid anticancer agents. These agents merge the natural scaffold's inherent biological activity with the increased potency provided by the attached moiety. Utilizing this approach, Rzepka *et al.* (2022) has developed a synthetic route for the preparation of two novel indole-functionalized derivatives of betulin, designated EB355A (130a) and EB365 (130b). Betulin, a naturally occurring pentacyclic triterpene present in birch bark, exhibits promising anticancer effects but is hindered by inadequate solubility and bioavailability. Inclusion of an indole moiety at the C-28 position enhances betulin's anticancer efficacy. The synthesized compounds were assessed for their anticancer efficacy against seven human cancer cell lines, including lung carcinoma, breast cancer, colorectal adenocarcinoma, and melanoma. The research indicated that MCF-7 breast cancer cells had the highest sensitivity to these compounds, especially 130a. Subsequent analysis revealed that 130a triggered cell cycle arrest in the G1 phase and prompted DNA fragmentation in MCF-7 cells, indicating apoptosis as a possible mode of action. The compounds did not substantially affect the viability of normal human cells, suggesting a potential for selective anticancer efficacy. *In silico* ADMET profiles for both compounds indicated that 130a and 130b are bioactive compounds exhibiting favourable intestinal absorption and comparatively low toxicity (Fig. 33). The compounds demonstrated the ability to traverse the blood–brain barrier, which may be advantageous for addressing brain metastases but could also present a risk of neurotoxicity. The targeted efficacy against MCF-7 cell lines indicates a promising direction for additional investigation in breast cancer therapy.^59^

**Fig. 34:**
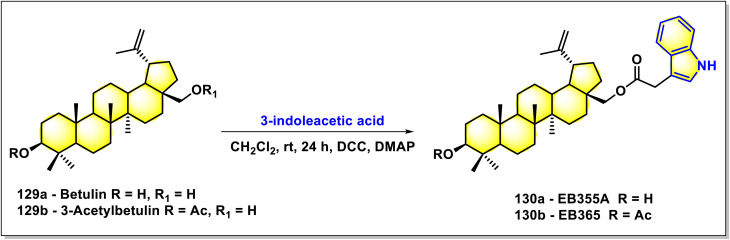
Synthetic route for the preparation of compounds 130a and 130b,^59^ reproduced from ref. 59 under the terms of the Creative Commons Attribution 4.0 license (CC BY 4.0), Copyright 2022, Z. Rzepka, E. Bębenek, E. Chrobak and D. Wrześniok.

The compounds 130a and 130b were synthesised *via* Steglich esterification of betulin (129a) and 3-acetylbetulin (129b) with 3-indoleacetic acid using DCC and DMAP in CH_2_Cl_2_ at room temperature (Fig. 34). This mild coupling strategy enables selective esterification at the C-28 hydroxyl group, facilitating incorporation of the indole moiety without affecting other functional groups. Notably, the reaction proceeds under neutral conditions and affords the desired ester-linked hybrids in moderate yields (52–58%), highlighting the suitability of Steglich conditions for sterically hindered substrates.^59^

### 3.8 Synthesis, antiproliferative effect, and topoisomerase II inhibitory activity of 3-methyl-2-phenyl-1*H*-indoles

**Fig. 35:**
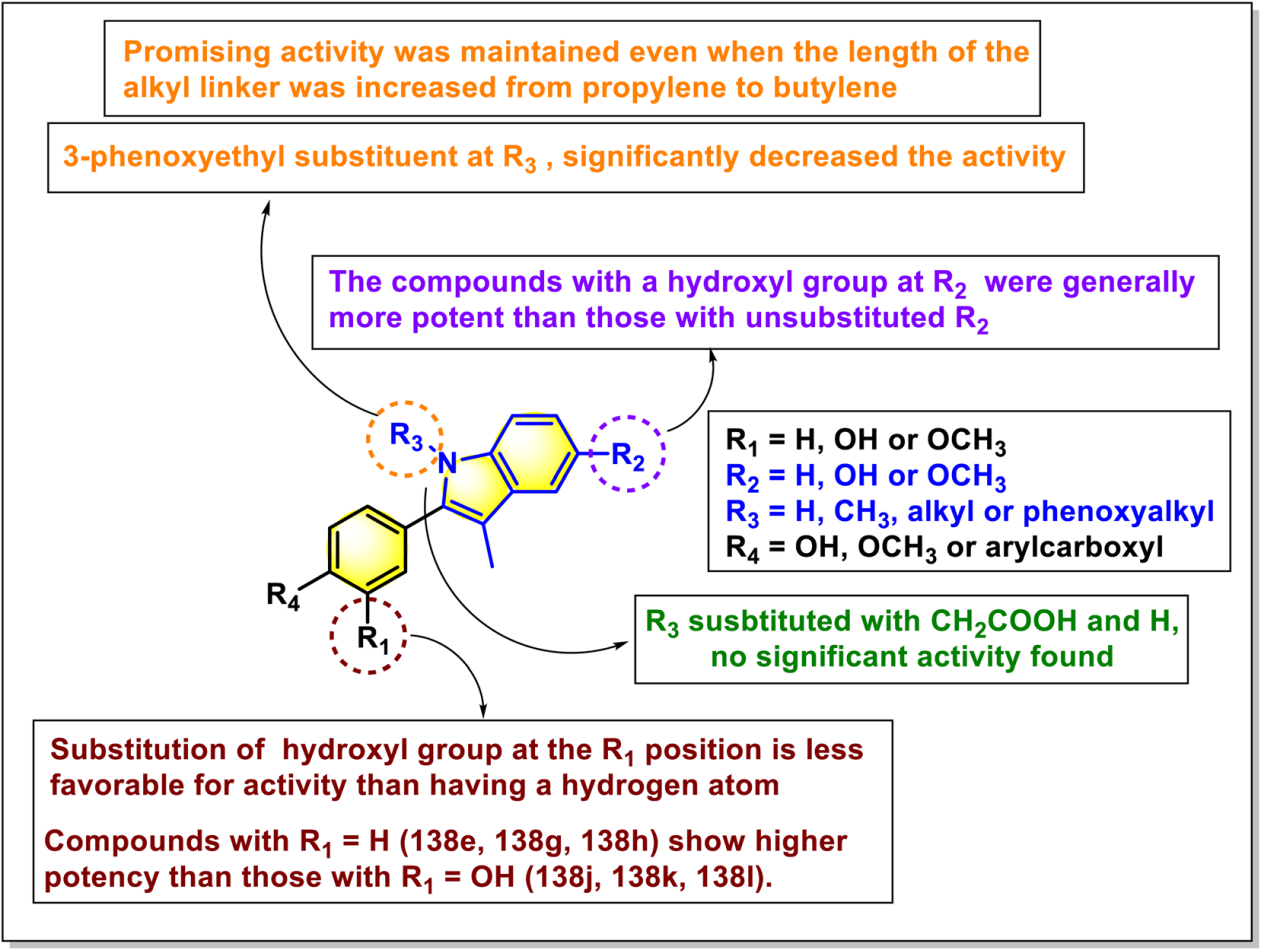
General structure of the synthesized 3-methyl-2-phenyl-1*H*-indole derivatives.^60^

This study holds particular importance as it not only highlights the antiproliferative capabilities of an indole scaffold that can be synthesized but also clarifies the mechanism underlying its biological effects, thereby connecting synthetic chemistry with translational oncology research. Notably, Zidar N. *et al.* (2020) synthesized and evaluated a variety of 3-methyl-2-phenyl-1*H*-indole compounds for their antiproliferative properties on human tumour cell lines. The compounds were evaluated against three human tumour cell lines: HeLa (cervical adenocarcinoma), A2780 (ovarian cancer), and MSTO-211H (biphasic mesothelioma). The research examined the structure–activity connections of these compounds by altering substituents at various places on the indole framework. The most potent compounds, 138g and 138h, had GI_50_ values below 5 µM in all evaluated cell lines. The impact of the most physiologically pertinent compounds on human DNA topoisomerase II (topo II) was investigated, discovering a strong link between antiproliferative effects and topo II inhibition. Subsequent examination of the most effective derivative, compound 138g, demonstrated its capacity to cause apoptosis in neoplastic cells. This study underscores the potential of 3-methyl-2-phenyl-1*H*-indole as a viable framework for the development of drugs exhibiting significant antiproliferative and anti-topoisomerase II actions (Fig. 35).^60^

**Fig. 36:**
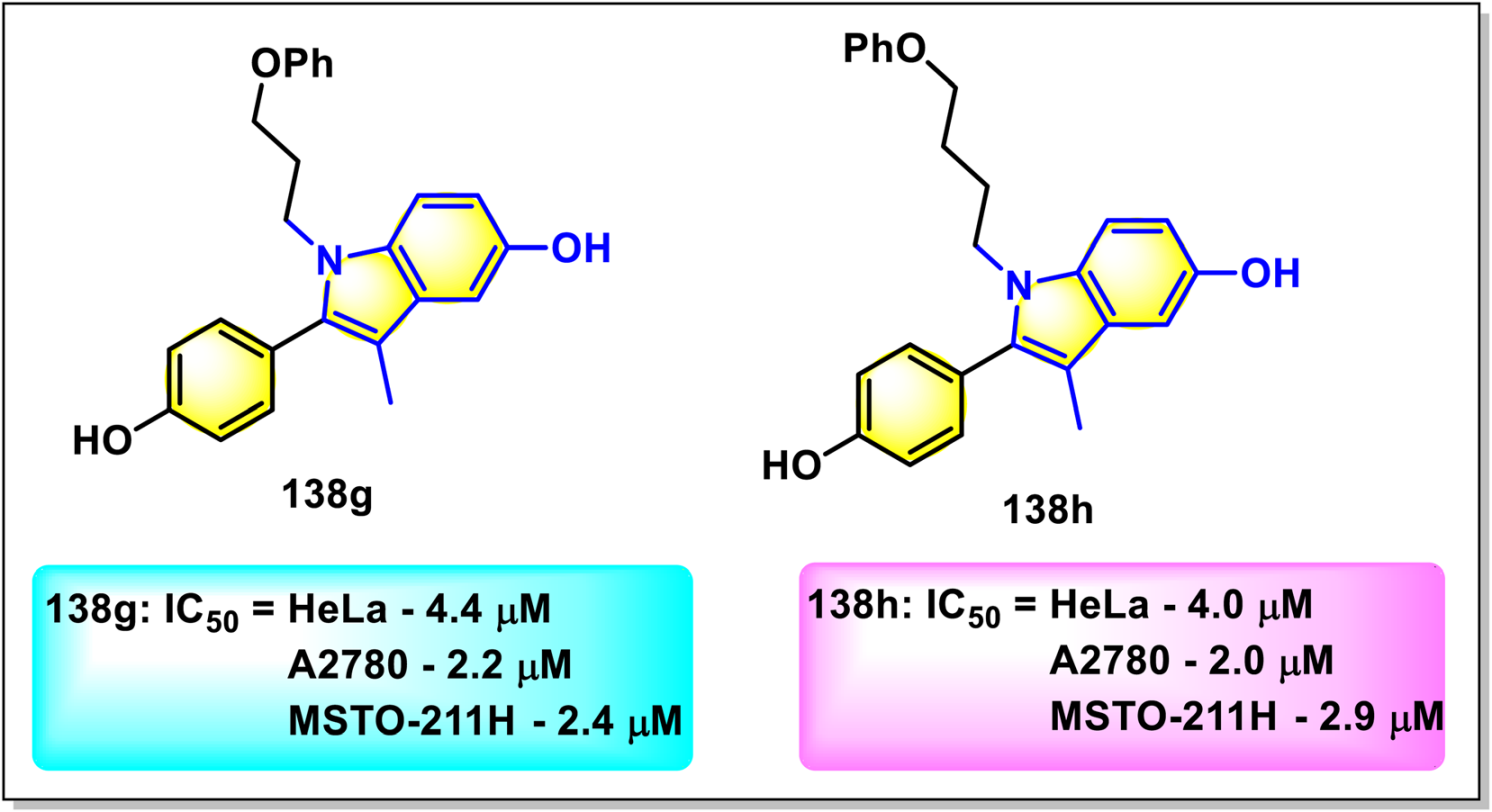
Structure of the most potent compounds 138g and 138h synthesized by Zidar N. *et al.*^60^

Significant insights into the structure–activity relationships of these compounds were provided, highlighting the critical role of hydroxyl groups at the R_2_ and R_4_ positions, as well as a preference for alkyl or phenoxyalkyl substituents at the R_3_ position. The study illustrates the relationship between topo II inhibition and antiproliferative actions, indicating a possible mechanism of action for these drugs. The induction of apoptosis by compound 138g suggests its potential as a lead molecule for further advancement in cancer therapies (Fig. 36). This research advances the quest for new chemotherapeutics with enhanced pharmaco-toxicological characteristics, responding to the pressing demand for innovative anticancer medicines.^60^

**Fig. 37:**
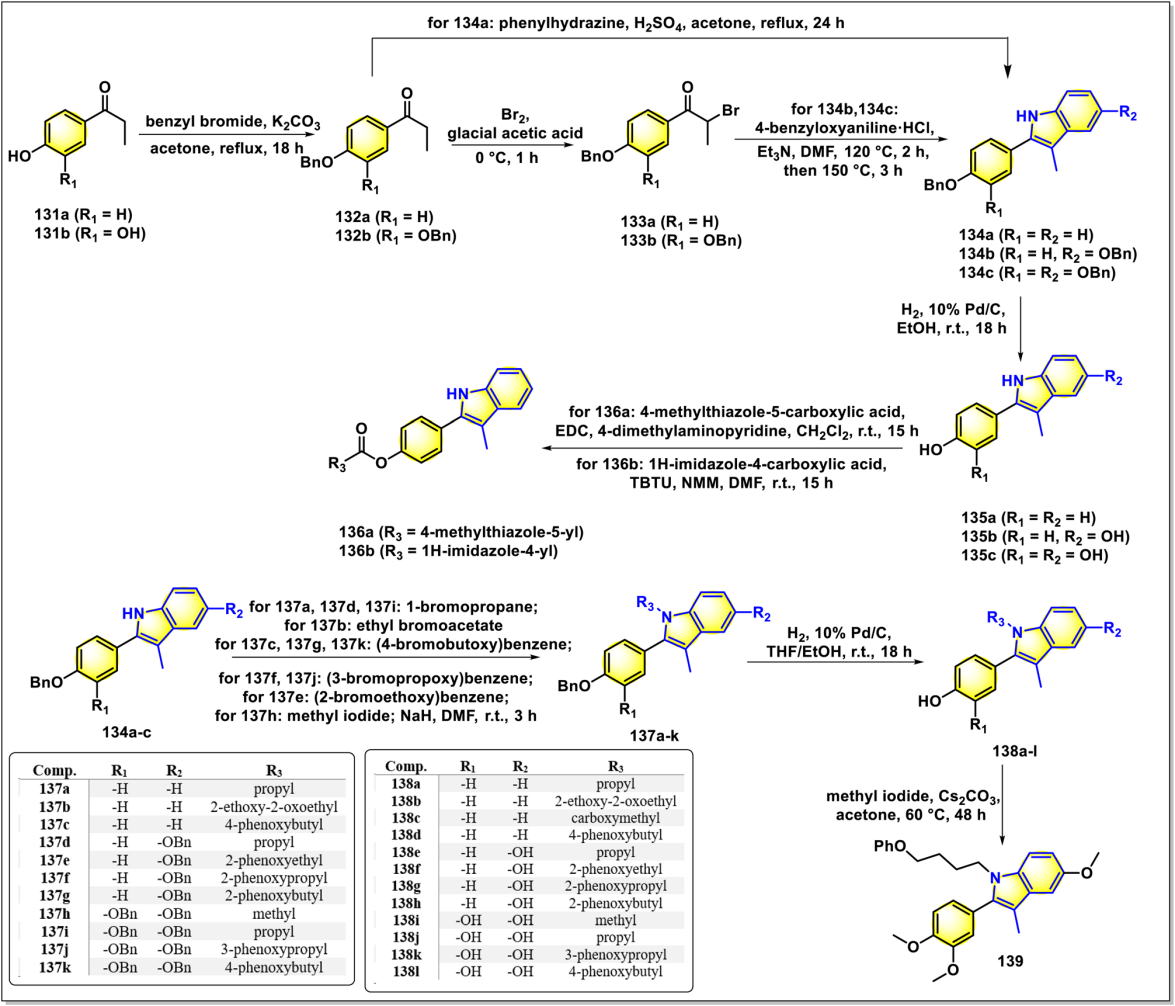
Synthetic pathway for 2-phenylindole derivatives 138a–l and 139, (ref. 60) reproduced from ref. 60 under the terms of the Creative Commons Attribution 4.0 license (CC BY 4.0), Copyright 2020, N. Zidar, D. Secci, T. Tomašič, L. P. Mašič, D. Kikelj, D. Passarella, A. N. Garcia Argaez, M. Hyeraci, L. D. *Via*.

The desired 3-methyl-2-phenyl-1*H*-indole derivatives were synthesised through a multistep sequence involving initial benzylation and bromination of acetophenone derivatives, followed by cyclisation with phenylhydrazine to construct the indole core (Fig. 37). Subsequent functional group transformations, including cross-coupling and substitution reactions, then enabled diversification at multiple positions to afford the final derivatives (138a–l and 139).

### 3.9 Synthesis of novel indole–thiazolidinone hybrid structures as a promising scaffold with anticancer potential

**Fig. 38:**
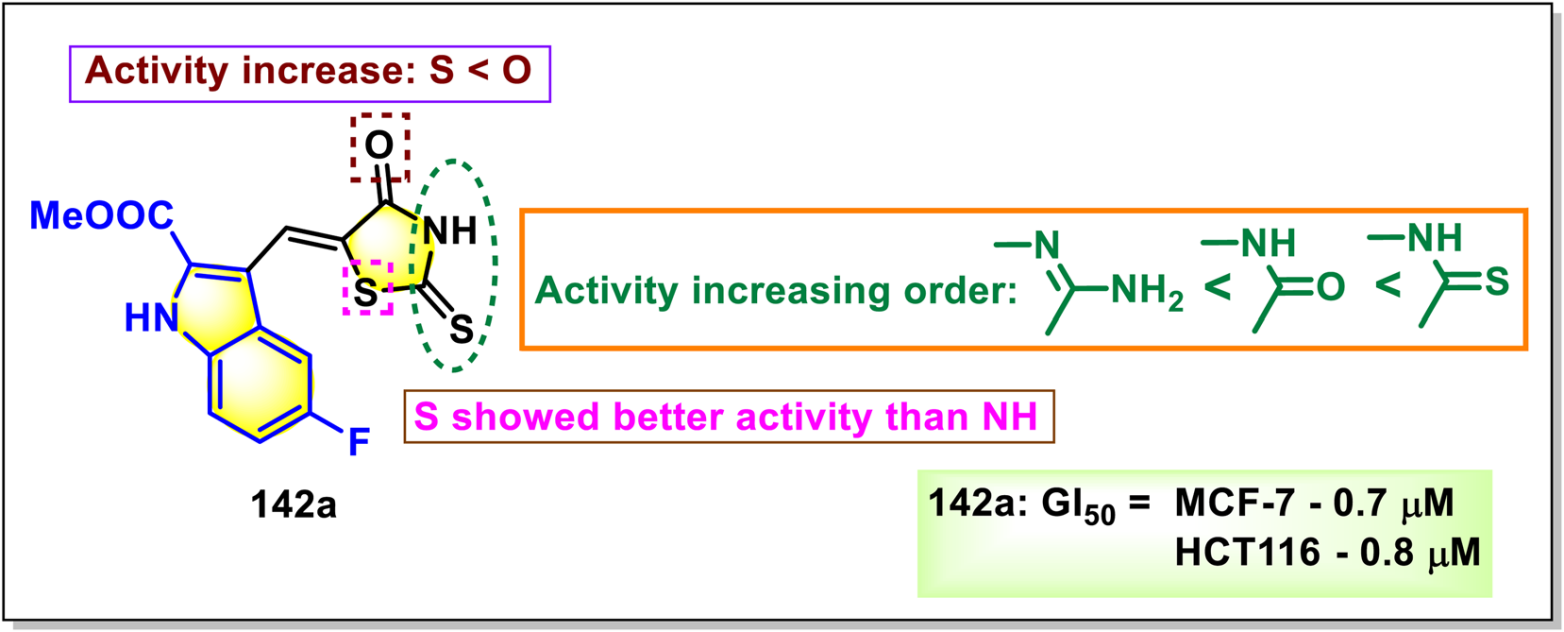
Structures and activity of the most potent indole–thiazolidinone hybrid compound 142a synthesized by Kryshchyshyn-Dylevych *et al.*^54^

The synthesis of indole–thiazolidinone hybrid compounds marks a notable breakthrough in the search for cancer treatments. By strategically combining two pharmacophoric elements into one framework, these compounds not only boost antiproliferative effectiveness but also exhibit selective toxicity against cancerous cells, thus tackling a major challenge in modern oncology. Towards this target, Kryshchyshyn-Dylevych A *et al.* (2021) demonstrated the synthesis and assessment of innovative indole–thiazolidinone hybrid compounds as prospective anticancer drugs. A series of hybrid compounds was developed and synthesized. Compound 142a (5-fluoro-3-(4-oxo-2-thioxothiazolidin-5-ylidenemethyl)-1*H*-indole-2-carboxylic acid methyl ester) was identified as the most potent compound. The investigation evaluated the cytotoxic effects of these compounds on multiple human cancer cell lines, including breast (MCF-7), colon (HCT116), liver (HepG2), cervical (HeLa), lung (A549), melanoma (WM793), and leukaemia (THP-1) cells. Compound 142a exhibited notable cytotoxicity, especially towards MCF-7 and HCT116 cells, with GI_50_ values of 0.7 µM and 0.8 µM, respectively (Fig. 38). The compound exhibited reduced toxicity towards non-malignant cells, suggesting a possible treatment window. Subsequent examinations of the mechanism of action demonstrated that compound 142a triggers apoptosis in cancer cells *via* caspase 3-, PARP1-, and Bax-dependent pathways. It also induced DNA damage in cancer cells, as demonstrated by the comet assay data.^54^

**Fig. 39:**
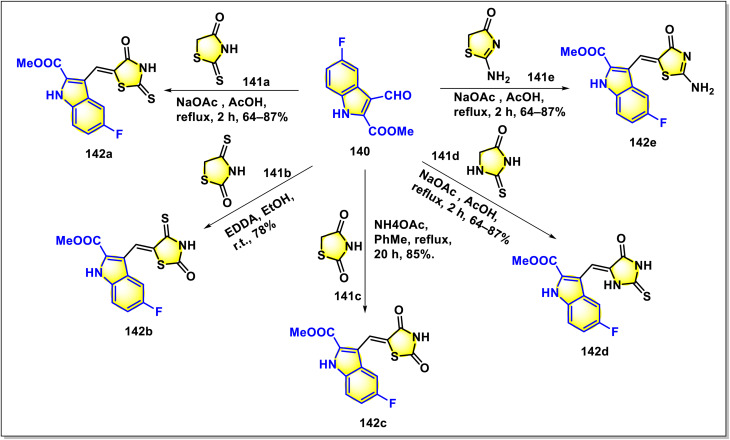
Synthesis of 5-fluoro-3-(azolidin-5-ylidenemethyl)-1*H*-indole-2-carboxylic acid methyl esters 142a–e,^54^ reproduced from ref. 54 with permission from Elsevier, *Bioorg. Med. Chem.*, 2021, **50**, 116453, Copyright 2021.

The indole–thiazolidinone hybrids were synthesised *via* a Knoevenagel condensation between 5-fluoro-3-formyl-1*H*-indole-2-carboxylic acid methyl ester (140) and various azolidinone derivatives (141a–d) under basic conditions (NaOAc/AcOH), leading to the formation of the corresponding arylidene-linked products (142a–d) (Fig. 39). This reaction facilitates the formation of a conjugated C=C linkage that effectively coupled the indole and azolidinone pharmacophores.^54^

### 3.10 Synthesis of indolyl pyrazole scaffolds as potential anti-cancer agents and their molecular modelling studies

**Fig. 40:**
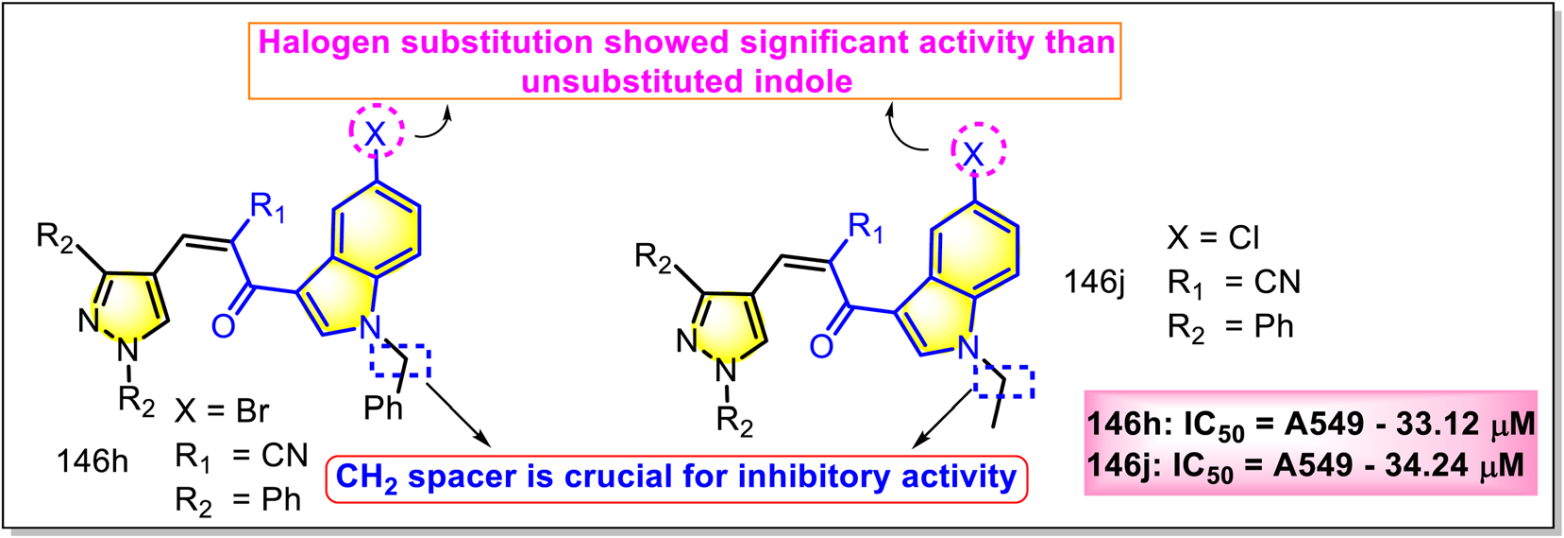
Chemical structures and inhibitory profiles of the most potent indole–pyrazole derivatives (146h and 146j) synthesised by Gaddam G. R. *et al.*^61^

The rational combination of indole and pyrazole pharmacophores into a single hybrid framework, directed by molecular docking studies aimed at the overexpressed BCL-2 protein, serves as a contemporary example of structure-based drug discovery. This approach greatly enhances the efficiency of identifying biologically significant anticancer candidates. In this context, Gaddam G. R. *et al.* (2020) developed a synthetic route for the novel indolyl pyrazole derivatives as prospective anti-cancer drugs. Novel compound entities were synthesised by integrating indole and pyrazole moieties, both recognized for their therapeutic capabilities, to explore potential anti-cancer action. The produced compounds were initially analysed by molecular docking experiments targeting the BCL-2 protein, which is recognized for its overexpression in several malignancies. Five compounds (146d, 146f, 146h, 146j, and 146l) were chosen for further assessment based on the docking scores and were evaluated for their cytotoxic efficacy against the A549 lung cancer cell line. Among these compounds, compounds 146h and 146j exhibited the most favourable outcomes, with IC_50_ values of 33.12 µM and 34.24 µM, respectively, in comparison to the reference doxorubicin (IC_50_ = 21.48 µM) (Fig. 40).^61^ The method of integrating two pharmacologically significant moieties (indole and pyrazole) yielded molecules with considerable anti-cancer efficacy. The application of green chemistry concepts in synthesis with molecular docking studies for compound selection exemplifies a contemporary and efficient methodology in drug discovery.

**Fig. 41:**
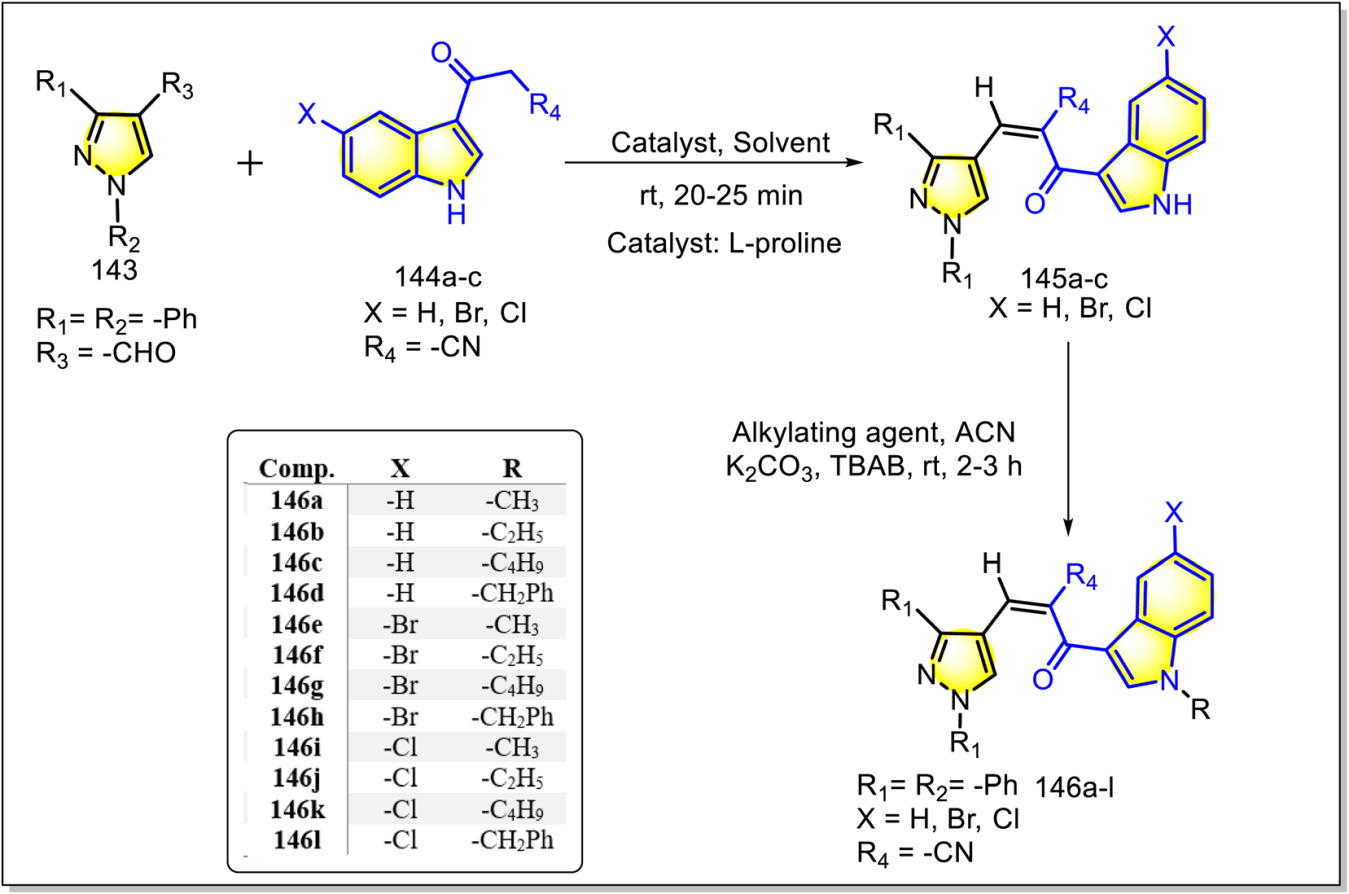
Multistep synthesis of preparation of indole–pyrazole hybrids 146a–l.^61^

The targeted molecules were synthesized *via*l-proline-catalysed Knoevenagel condensation between indole aldehyde (143) and active methylene compounds (144a–c) in ethanol, affording intermediates 145a–c. Subsequent *N*-alkylation using appropriate alkylating agents in the presence of K_2_CO_3_ and TBAB in acetonitrile furnished the final derivatives 146a–l (Fig. 41).^61^

## 4 Pyrimidines

Pyrimidine-based derivatives have emerged as indispensable pharmacophores in anticancer drug discovery, owing to their unique capacity to engage a wide array of signalling proteins and regulatory kinases.^35,62^ Numerous pyrimidine derivatives have demonstrated significant potential as antitumor agents through various mechanisms. For example, hybrid molecules incorporating pyrimidine scaffolds with triazole or imidazole units have demonstrated dual inhibition of topoisomerase II and tubulin, offering a promising approach for overcoming drug resistance and enhancing therapeutic efficacy.^63^ Similarly, pyrimidine derivatives fused with thiophene have been reported as potent inhibitors of the EGFR, even against resistant mutations such as T790M and L858R/T790M.^64^ Researchers are focusing on rationally designed hybrid pyrimidine-based molecules that combine multiple pharmacophores into a single entity, thereby enabling the simultaneous modulation of multiple oncogenic pathways. In addition, substituted pyrimidine derivatives bearing aryl, diamino pyrimidine derivatives or trifluoromethyl groups have displayed potent inhibition of kinases such as Mer, Tyro3, and focal adhesion kinase (FAK), demonstrating both antiproliferative and anti-angiogenic properties.^65–67^ Collectively, these studies highlight the versatility of the pyrimidine scaffold as a privileged structure in anticancer drug discovery, offering new avenues for the design of next-generation therapeutic agents with improved efficacy and reduced resistance.

### 4.1 Synthesis of new pyrimidine–triazole derivatives and investigation of their anticancer activities

**Fig. 42:**
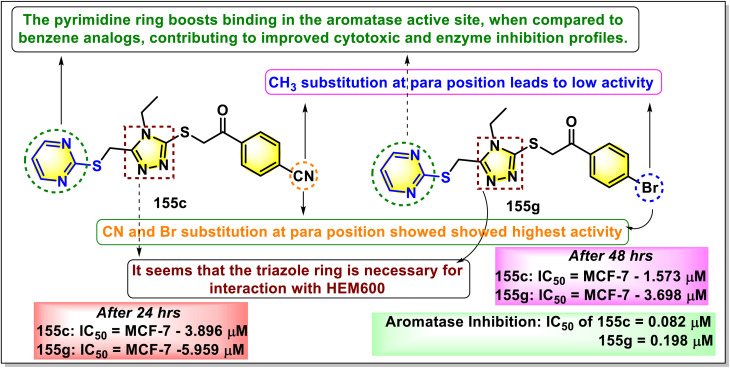
SAR analysis and biological evaluation of the most potent pyrimidine–triazole hybrids synthesized by Osmaniye *et al.*^68^

The effective alignment of molecular docking results with *in vitro* biological activity not only confirms the validity of the computational screening method used but also offers medicinal chemists dependable structural insights for further refining this promising category of aromatase-targeting anticancer agents. To validate this approach, Osmaniye *et al.* (2022) conducted a study on the synthesis and examination of novel pyrimidine–triazole derivatives for their potential as anticancer medicines, particularly as aromatase inhibitors in breast cancer therapy. Ten new compounds were synthesised and assessed for their anticancer properties against multiple cell lines, particularly the MCF-7 breast cancer cell line. Cytotoxicity assays were performed utilizing the MTT technique on various cell lines, including A549, MCF-7, and NIH3T3. Aromatase inhibition experiments were conducted to assess the efficacy of drugs as aromatase inhibitors. The findings indicated that two compounds, 155c and 155g, show substantial anticancer efficacy against the MCF-7 cell line after 48 h of incubation with IC_50_ values of 1.573 µM and 3.698 µM respectively and exhibited strong aromatase inhibition with IC_50_ values of 0.082 µM and 0.198 µM (Fig. 42). Molecular docking analyses were performed to elucidate the binding mechanisms of these drugs with the aromatase enzyme. The physicochemical properties and drug-likeness of the compounds were assessed by computational approaches. The integration of *in vitro* experiments and computer analyses facilitated a thorough assessment of the compounds' viability as therapeutic candidates. These findings enhance the continuing initiatives to advance breast cancer treatments and address challenges such as drug resistance and adverse effects linked to current medicines.^68^

**Fig. 43:**
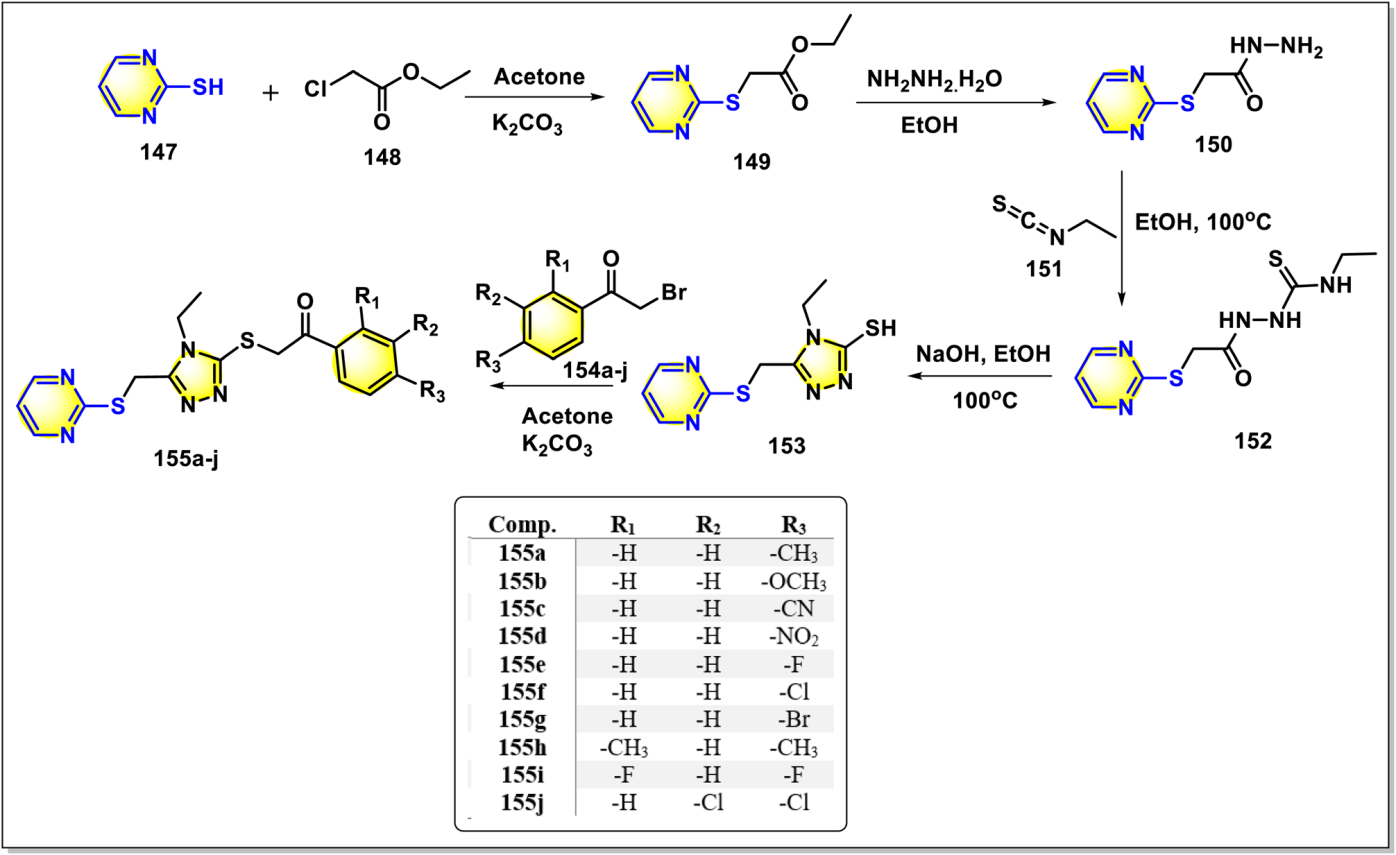
Multi-step synthetic strategy for the preparation of pyrimidine-triazole hybrids (155a–j),^68^ reproduced from ref. 68 with permission from John Wiley and Sons, *Chem. Biodiversity*, 2022, **19**, e202200216, Copyright 2022.

As illustrated in Fig. 43, the target pyrimidine–triazole hybrids (155a–j) were prepared *via* a multi-step synthetic strategy. The sequence initiated with the *S*-alkylation of pyrimidine-2-thiol (147) using ethyl 2-chloroacetate (148), followed by the hydrazinolysis of the resulting ester (149) to yield acetohydrazide 150. Treatment of 150 with ethyl isothiocyanate (151) furnished the thiourea intermediate 152, which underwent a base-promoted intramolecular cyclisation to construct the triazole core (153). Ultimately, structural diversification was achieved by alkylating 153 with various substituted phenacyl bromides (154a–j) to provide the final hybrid derivatives (Fig. 43).^68^

### 4.2 Discovery of a pyrimidine compound endowed with antitumor activity

**Fig. 44:**
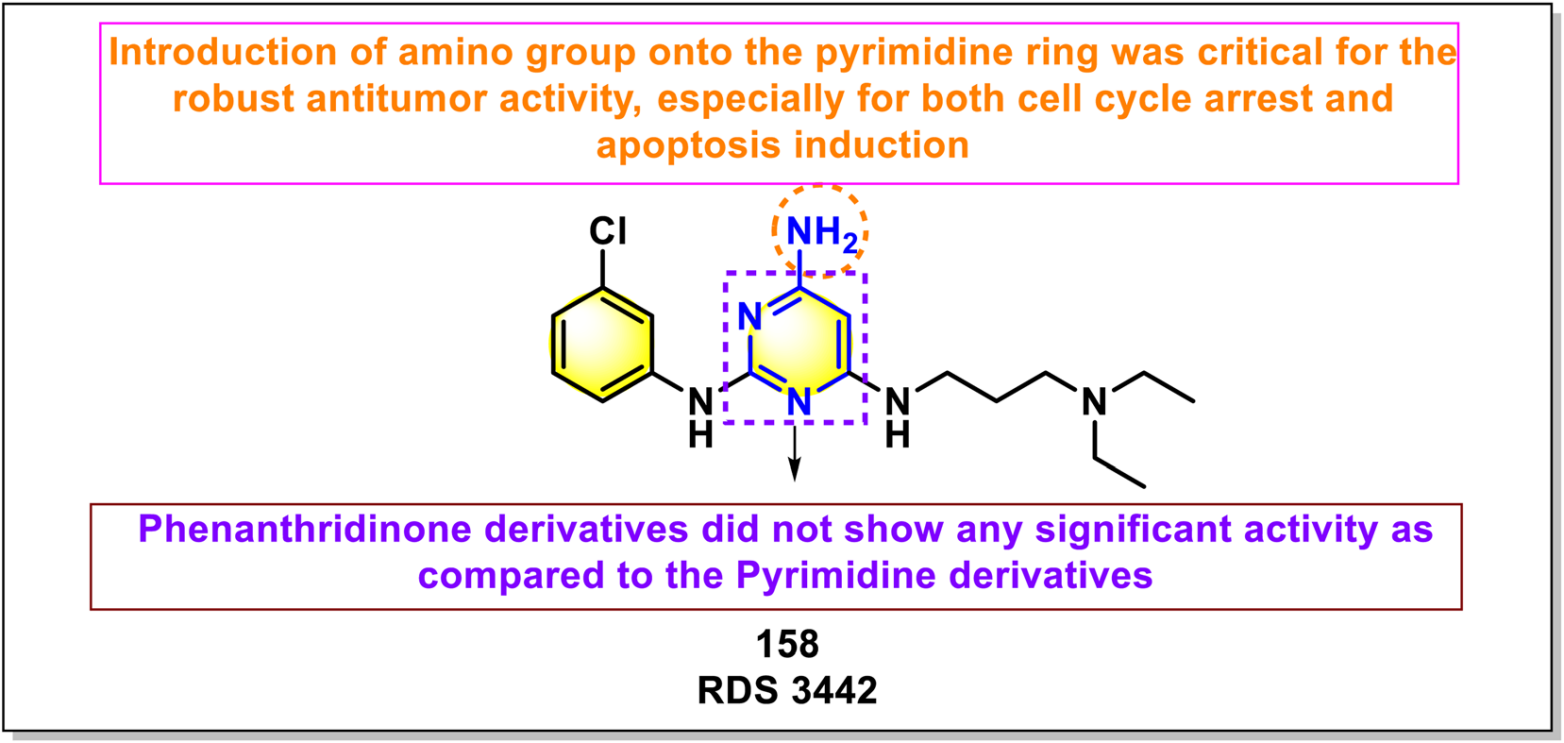
Chemical structure and functional motifs of RDS 3442 (158).^69^

Taglieri L *et al.* (2020) introduced a simple two-step method involving nucleophilic aromatic substitution to achieve a new pyrimidine compound, RDS 3442 (158), which greatly enhances its potential for practical use. This is attributed to the structural simplicity and modular design of the dichloropyrimidine framework, facilitating the future development of analog derivatives and systematic exploration of structure–activity relationships. The research examines the impact of this compound on three human cancer cell lines: triple-negative breast cancer (MDA-MB231), colon carcinoma (HT-29), and glioblastoma multiforme (U-87). The researchers discovered that 158 effectively suppresses cell growth, halts the cell cycle at the G0/G1 phase, and triggers apoptosis across all three cancer types. The compound functions by upregulating cyclin-dependent kinase (CDK) inhibitors p21 and p27, which are crucial for cell cycle regulation. At reduced concentrations (20 µM), 158 precipitates cell cycle arrest without inducing apoptosis (Fig. 44). At elevated concentrations (30–50 µM), it induces apoptosis *via* caspase activation and an increased Bax/Bcl-2 ratio. The work highlights the bifunctional role of p21, which functions as a tumour suppressor in the nucleus while inhibiting apoptosis in the cytoplasm. The compound's capacity to elicit cell cycle arrest at lower concentrations and apoptosis at elevated concentrations indicates a dose–dependent mechanism that may be utilized in cancer treatment. The work emphasizes the significance of p21 and p27 in cancer growth and treatment, illustrating how their expression levels and cellular localization can profoundly influence tumour cell behaviour and therapeutic response.^69^ These discoveries create opportunities for tailored cancer therapies and establish a basis for further exploration of pyrimidine-based drugs in oncology.

**Fig. 45:**
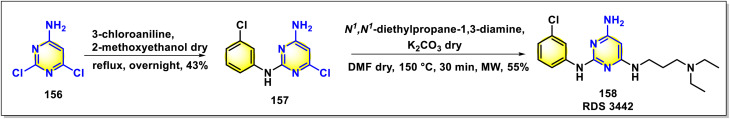
Synthesis of RDS 3442 (158),^69^ reproduced from ref. 69 with permission from Springer Nature, *Invest. New Drugs*, 2020, **38**, 39–49, Copyright 2019.

As depicted in Fig. 45, the synthesis of RDS 3442 (158) was achieved through a two-step nucleophilic aromatic substitution sequence on a dichloropyrimidine scaffold (156). The sequence commenced with the substitution of 156 using 3-chloroaniline under reflux conditions to generate intermediate 157. Subsequent amination of 157 with *N*,*N*-diethylethane-1,2-diamine, using K_2_CO_3_ as a base in DMF, afforded the target compound 158. This efficient stepwise functionalisation underscores the versatility of the pyrimidine core for the incorporation of diverse, biologically relevant amine moieties.

### 4.3 Design, synthesis, and antitumor activity evaluation of pyrimidine derivatives containing 4-hydroxypiperidine group

**Fig. 46:**
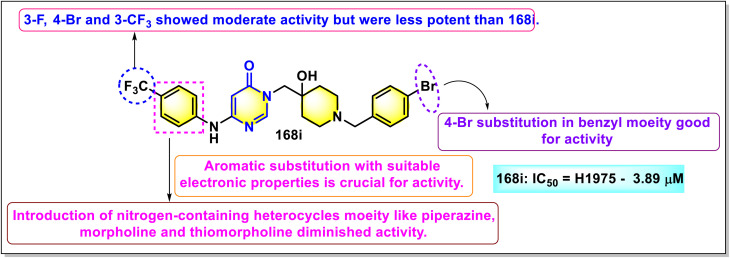
Structural features of the most potent compound (168i) in a series of novel compounds synthesised by Chi L. *et al.*^70^

The strategic integration of 4-hydroxypiperidine units into the pyrimidine structure, along with a multi-step synthesis method using 4,6-dichloropyrimidine as a flexible starting point, not only enhances the structural variety of pyrimidine-based anticancer compounds but also establishes a strong and adaptable synthetic framework for systematically investigating structure–activity relationships. This approach aims to develop more effective and selective next-generation anticancer drugs. Chi L. *et al.* (2023) carried out a study on the design, synthesis, and assessment of new pyrimidine derivatives with 4-hydroxypiperidine groups as prospective anticancer agents. A series of compounds (167a–g and 168a–n) was synthesized and evaluated for their antiproliferative activity against four human cancer cell lines: MGC-803, PC-3, A549, and H1975. The antiproliferative efficacy of the produced compounds was assessed *via* the MTT assay, with 5-FU serving as a positive control. 168i demonstrated the most promising results among the evaluated compounds, displaying excellent antiproliferative activity against H1975 cells with an IC_50_ value of 3.89 µM. Subsequent investigations on 168i showed its capacity to disrupt colony formation and cellular migration in H1975 cells (Fig. 46). It also induced apoptosis in a dose–dependent fashion and resulted in cell cycle arrest during the S phase.^70^ These results indicate that 168i may serve as a potential lead for further research.

**Fig. 47:**
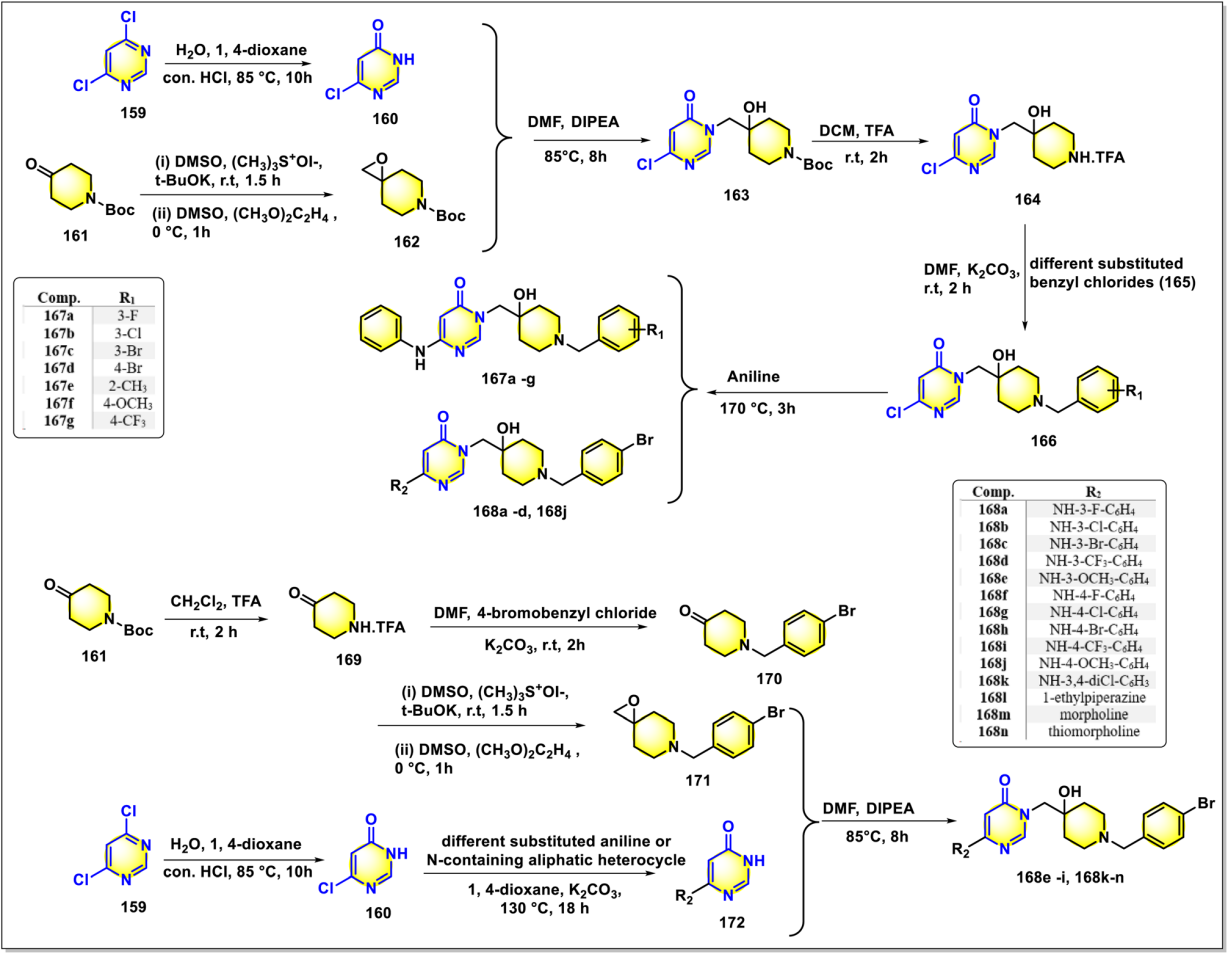
Synthetic route for the construction of functionalized pyrimidine derivatives with 4-hydroxypiperidine groups (168a–n and 172e–n),^70^ reproduced from ref. 70 with permission from Springer Nature, *Med. Chem. Res.*, 2023, **32**, 2125–2137, Copyright 2023.

The synthesis of pyrimidine derivatives (168a–n and 172e–n) is outlined in Fig. 47. This synthesis comprises a multi-step sequence utilizing 4,6-dichloropyrimidine (159) as the starting scaffold. The reaction proceeded *via* sequential nucleophilic substitutions for the incorporation of hydroxyl groups and linker moieties, followed by *N*-alkylation and amination to generate the key intermediates. Finally, extensive structural diversification was achieved by coupling these intermediates with various substituted anilines and *N*-containing heterocycles.

A series of compounds (167a–g and 168a–n) was synthesized utilizing this modular synthetic methodology.^70^

### 4.4 Design and development of new substituted pyrimidine hybrids with imidazole and triazole: exploring utility as an anticancer agent *via* human topoisomerase-II and tubulin inhibition

**Fig. 48:**
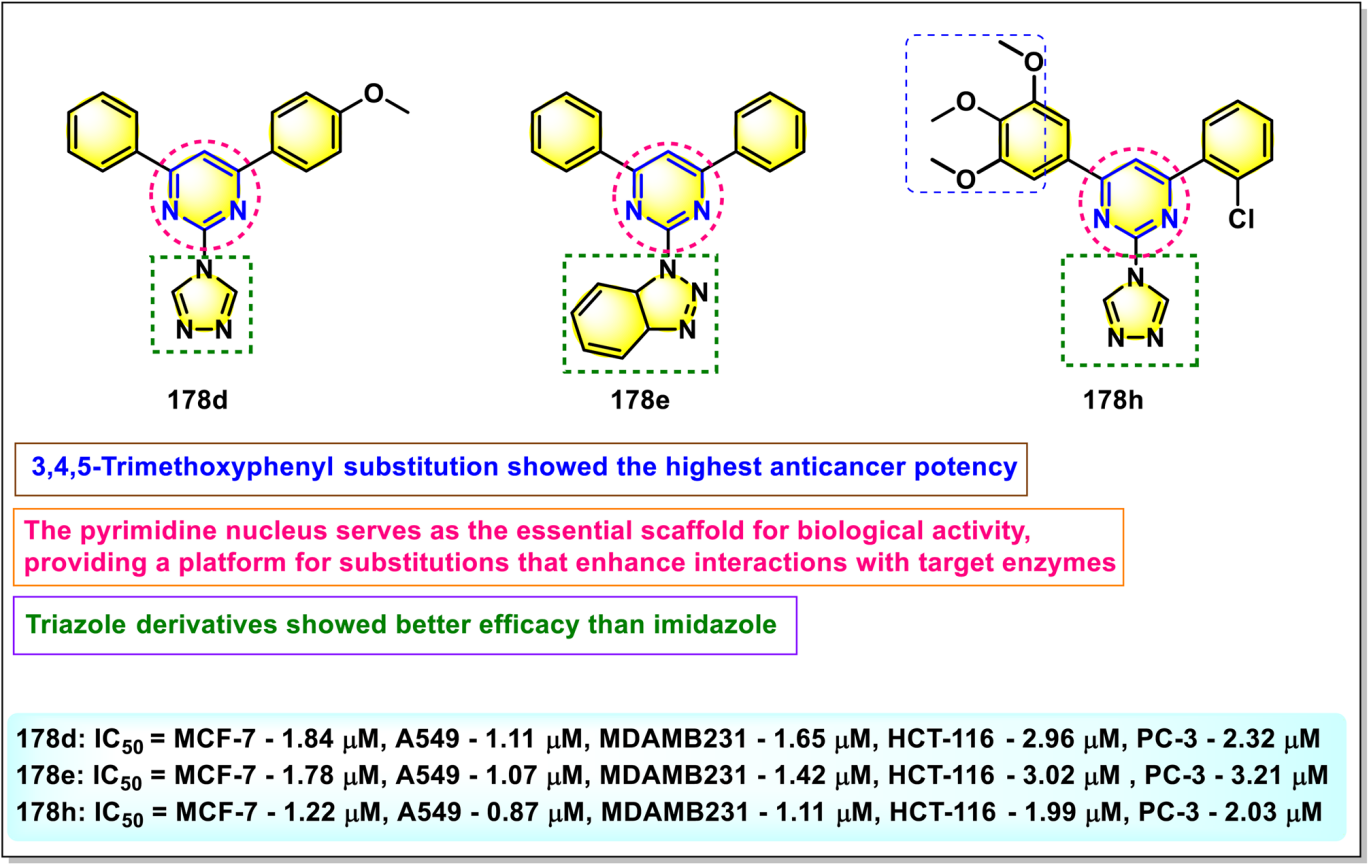
Potent pyrimidine-based derivatives synthesized by Yadav U. P. *et al.* and their structure–activity relationship (SAR) profile.^63^

The use of a highly effective multicomponent reaction pathway for creating pyrimidine-imidazole and pyrimidine–triazole hybrids, supported by extensive *in vitro* mechanistic studies, molecular docking analyses, and apoptosis induction data, highlights the translational significance of this modular synthetic approach in speeding up the discovery of structurally varied, pharmacologically powerful anticancer candidates that can overcome the limitations of single-target chemotherapy agents. Given the significance, Yadav U. P. *et al.* (2025) developed a synthetic route for the preparation of pyrimidine-based hybridized compounds with imidazole and triazole using a multicomponent reaction (MCR) pathway. The compounds were investigated for the use of synthetics as cancer-fighting agents *via* tubulin inhibition and human topoisomerase-II. In comparison to colchicine and etoposide, which were employed as positive controls, the synthesized 178d, 178e, and 178h were found to be potent anticancer agents when tested on five distinct cancer cell lines (Fig. 48). Additionally, it was found that these synthetics did not cause significant damage to healthy cells, indicating that they primarily target and kill cancer cells. An *in vitro* experiment conducted demonstrated that these compounds blocked both hTopoII and tubulin, and the mechanistic findings were validated by molecular docking studies. Furthermore, it was demonstrated that the compounds induced apoptosis, a secondary method of killing cancer cells. They stopped the cell cycle at the G2/M phase, reduced oxidative stress, and killed cancer cells.^63^

**Fig. 49:**
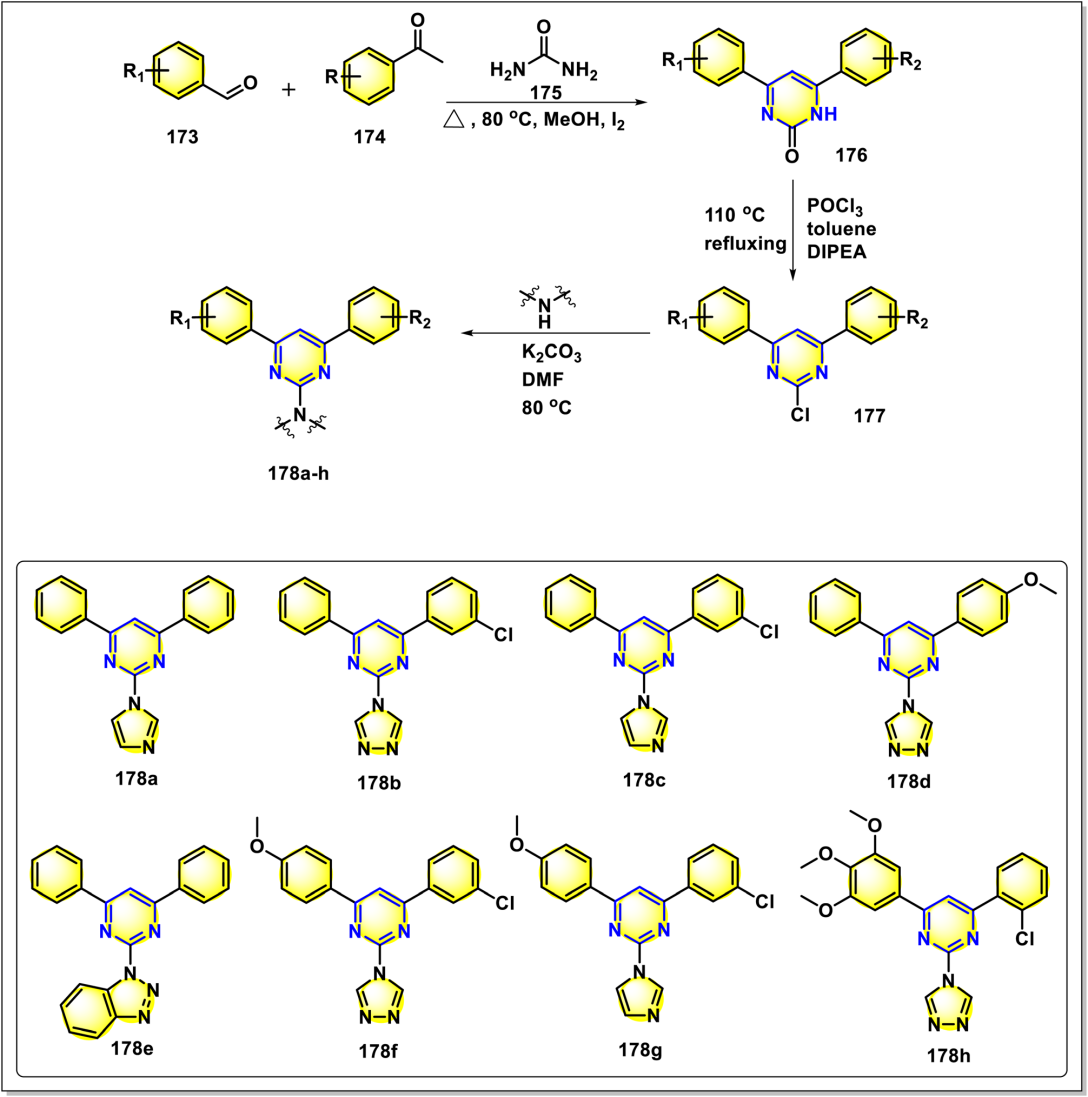
Synthetic route for the preparation of pyrimidine-based target compounds 178a–h,^63^ reproduced from ref. 63 with permission from Elsevier, *Bioorg. Chem.*, 2025, **158**, 108334, Copyright 2025.

As illustrated in Fig. 49, the pyrimidines with different aromatic rings (178a–h) were synthesized *via* an efficient multicomponent reaction pathway. The sequence began with an iodine-catalysed condensation of aryl aldehydes (173), ketones (174), and urea (175) to form the pyrimidine core (176). This intermediate was activated *via* chlorination with POCl_3_ to yield 177, which then underwent a K_2_CO_3_-mediated nucleophilic aromatic substitution with various imidazole or triazole derivatives to afford the final compounds. This modular synthetic strategy allows for the highly efficient integration of multiple pharmacophores.

### 4.5 Design and synthesis of 5-aryl-substituted phenylpyrimidine-2,4-diamine derivatives as novel Mer and Tyro3 kinase inhibitors

**Fig. 50:**
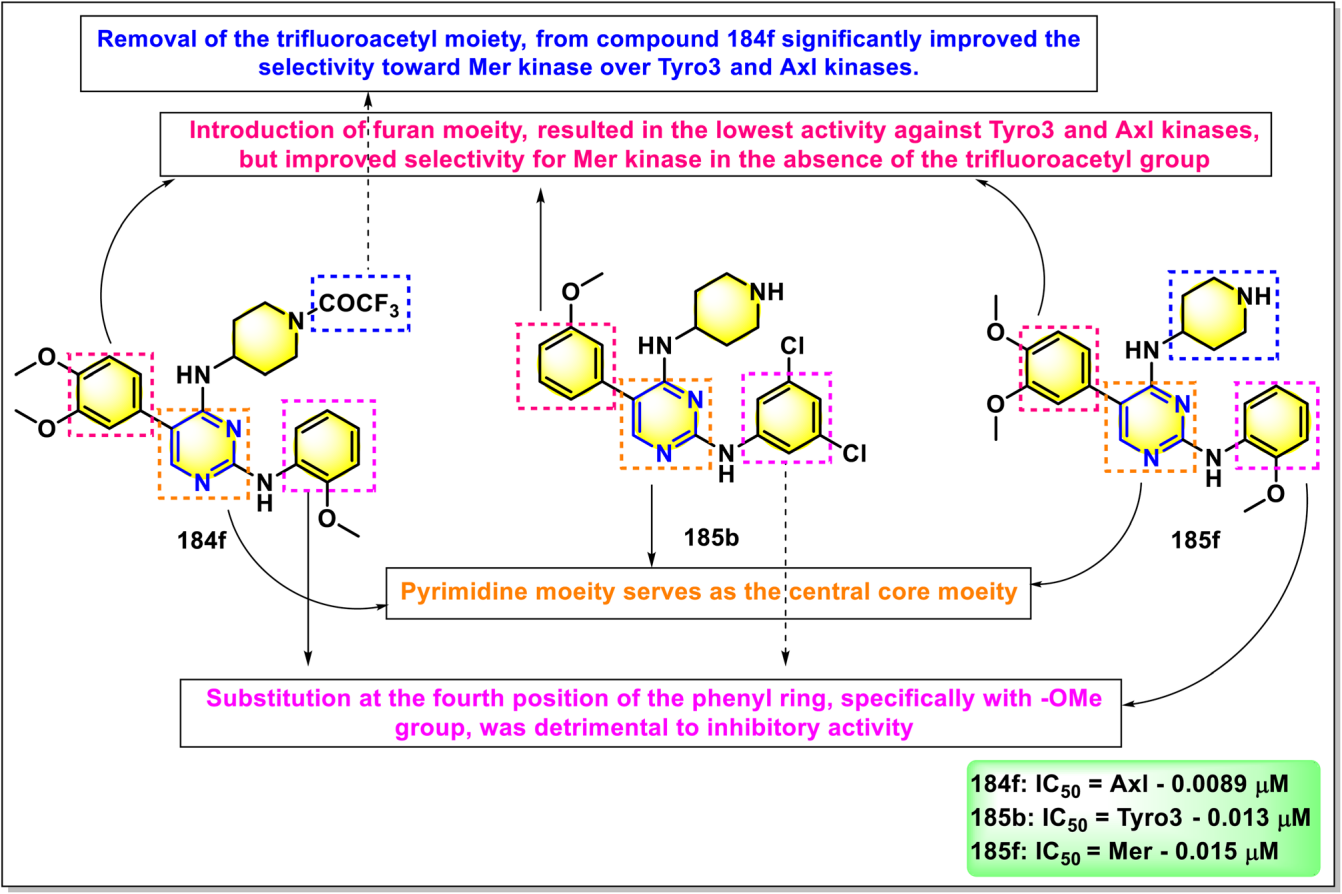
Structure–activity relationship (SAR) of the most potent 5-aryl-substituted phenylpyrimidine-2,4-diamine derivatives synthesized by Kim Y. *et al.*^65^

The convergent multi-step synthetic approach utilizing Pd-catalyzed cross-coupling reactions not only underscores the synthetic accessibility of this series of compounds but also offers a strong and adaptable platform for the swift creation of structural analogs to further enhance selectivity and potency profiles against specific TAM kinases. Taking it further, Kim Y. *et al.* (2020) demonstrated a synthetic route for the preparation of 5-aryl-substituted phenylpyrimidine-2,4-diamine derivatives as innovative inhibitors of TAM (Tyro3, Axl, Mer) kinases. The study aims to develop effective and selective inhibitors for these kinases, which are crucial to numerous biological processes and are linked to cancer progression. A range of compounds were synthesised and assessed for their inhibitory effects against TAM kinases. Comprehensive structure–activity relationship (SAR) analyses were performed, specifically concentrating on alterations at the fifth position of the pyrimidine ring. Minor modifications in substituents significantly influence the selectivity and efficacy of the drugs against various TAM kinases. Compounds 184f, 185b, and 185f demonstrated significant inhibitory efficacy and remarkable selectivity for Axl, Tyro3, and Mer kinases, respectively (Fig. 50). Molecular docking experiments were utilized to elucidate the binding mechanisms of the most effective drugs with their corresponding target kinases. These studies elucidated the relationships between the drugs and the kinase binding sites, clarifying the reported selectivity and efficacy.^65^ These results indicate that these compounds can be further investigated to develop innovative anticancer medicines with enhanced efficacy and reduced adverse effects.

**Fig. 51:**
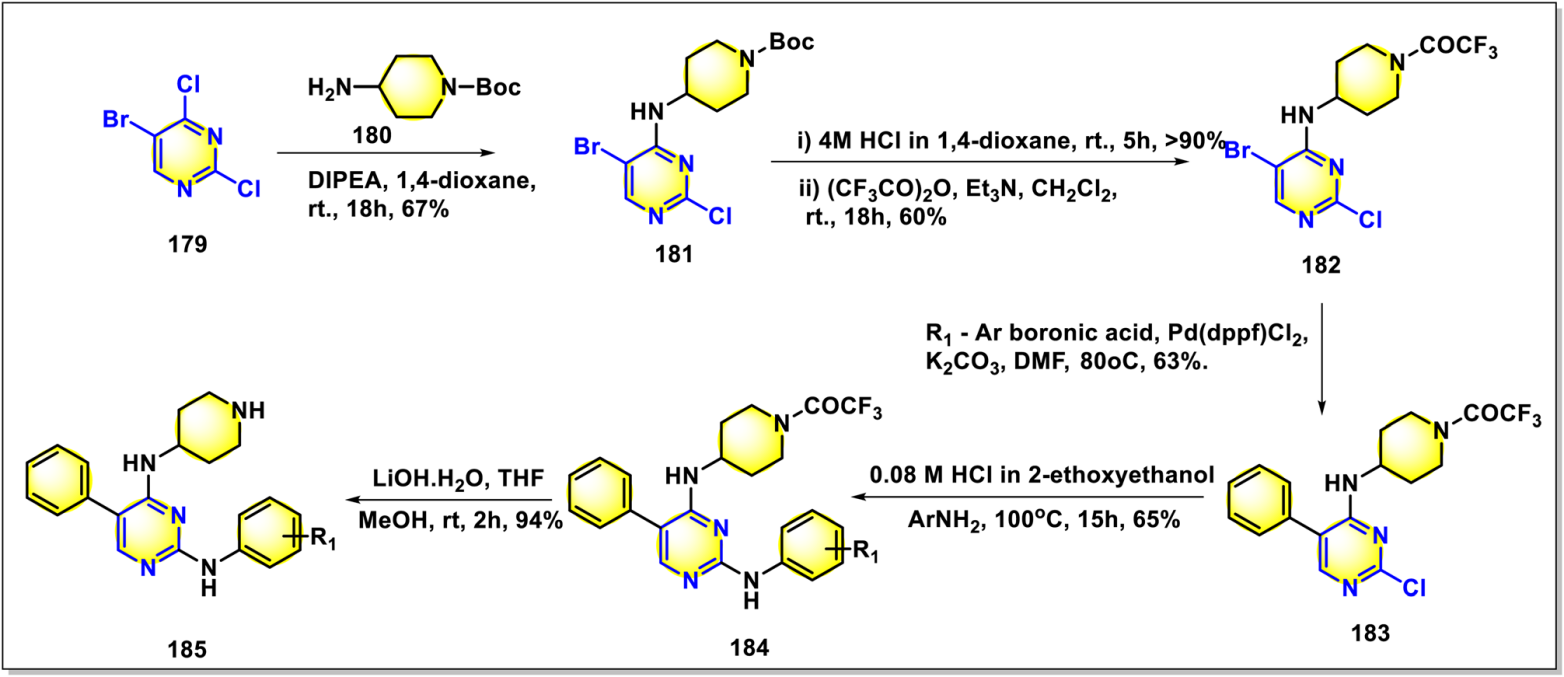
Synthetic route for the preparation of *N*^4^-piperidinyl-5-phenyl-*N*^2^-arylpyrimidine-2,4-diamine derivatives,^65^ reproduced from ref. 65 with permission from John Wiley and Sons, *Bull. Korean Chem. Soc.*, 2021, **42**, 206–211, Copyright 2020.

The synthetic strategy for the desired *N*^4^-piperidinyl-5-phenyl-*N*^2^-arylpyrimidine-2,4-diamine derivatives (184–185) is depicted in Fig. 51. The synthesis commenced with a nucleophilic substitution reaction between the dichloropyrimidine core and a Boc-protected piperidine amine (180), which selectively afforded the mono-substituted intermediate 181. Subsequently, deprotection and functional group elaboration of 181 yielded intermediate 182. Structural diversification was then achieved *via* a Pd-catalysed cross-coupling reaction with diverse aryl boronic acids, providing intermediate 183. A final sequence of substitution and deprotection steps furnished the target compounds (184–185).

### 4.6 New fused pyrimidine derivatives with anticancer activity: synthesis, topoisomerase II inhibition, apoptotic inducing activity, and molecular modelling study

**Fig. 52:**
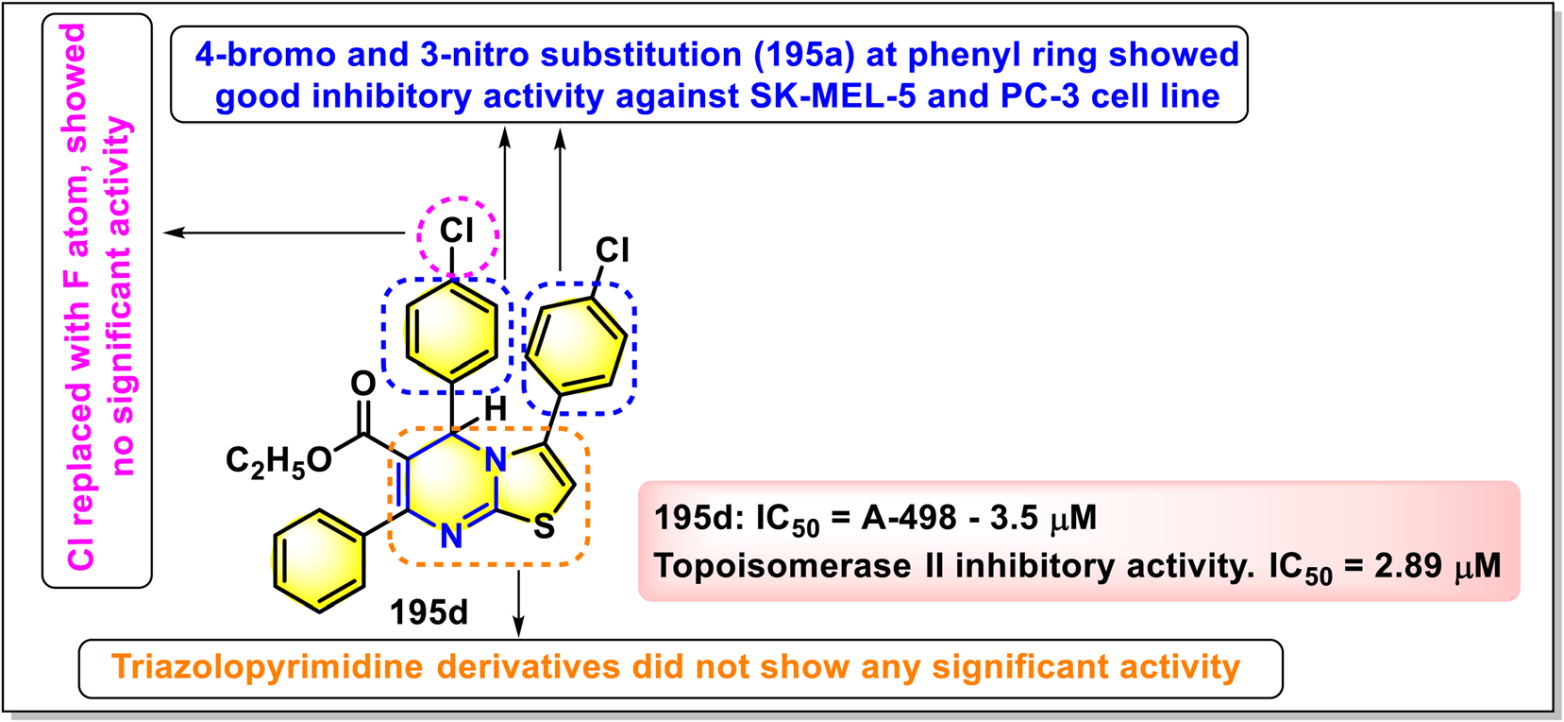
Structural highlights and inhibitory profile of potent thiazolopyrimidine derivatives synthesised by Nemr M. T. *et al.*^71^

Nemr M. T. *et al.* (2020) demonstrated the synthesis and assessment of novel fused pyrimidine derivatives as potential anticancer agents, primarily targeting topoisomerase II. The research focuses on triazolopyrimidines and thiazolopyrimidine hydrobromides, which were developed and produced as potential topoisomerase II inhibitors. The National Cancer Institute (NCI) of the United States evaluated the compounds against 60 human cancer cell lines. Among the produced compounds, compound 195d showed the most promising activity, exhibiting substantial inhibition of the renal cell line A498 with 92.46% inhibition (IC_50_ = 3.5 µM). Compound 195d also displayed significant inhibitory effect against both leukaemia cell line (K-562) and prostate cancer cell line (PC-3) with 68.54 and 58.73 growth inhibition percentage, respectively. Subsequent analysis of the compound revealed its ability to induce cell cycle arrest at the G2/M phase, leading to reduced cell proliferation and enhanced apoptotic activity. Moreover, compound 195d exhibited significant topoisomerase II inhibitory activity (IC_50_ 2.89 µM), similar to the reference compound doxorubicin (IC_50_ 2.67 µM). Multiple biological assessments, encompassing *in vitro* anticancer screening, cell cycle analysis, annexin-V apoptosis testing, and topoisomerase II inhibitory activity evaluations were performed, and molecular modelling investigations were conducted to elucidate the binding interactions of 195d with the topoisomerase II enzyme (Fig. 52). Compound 195d exhibits selective activity against the renal cell line A498, indicating its promise as a targeted therapy for renal cancer. Moreover, the analogous topoisomerase II inhibitory efficacy of compound 195d to doxorubicin, a recognized anticancer agent, underscores its potential as a lead molecule for subsequent development. Molecular modelling studies offer significant insights into structure–activity connections, potentially guiding future optimization efforts within this class of molecules.^71^

**Fig. 53:**
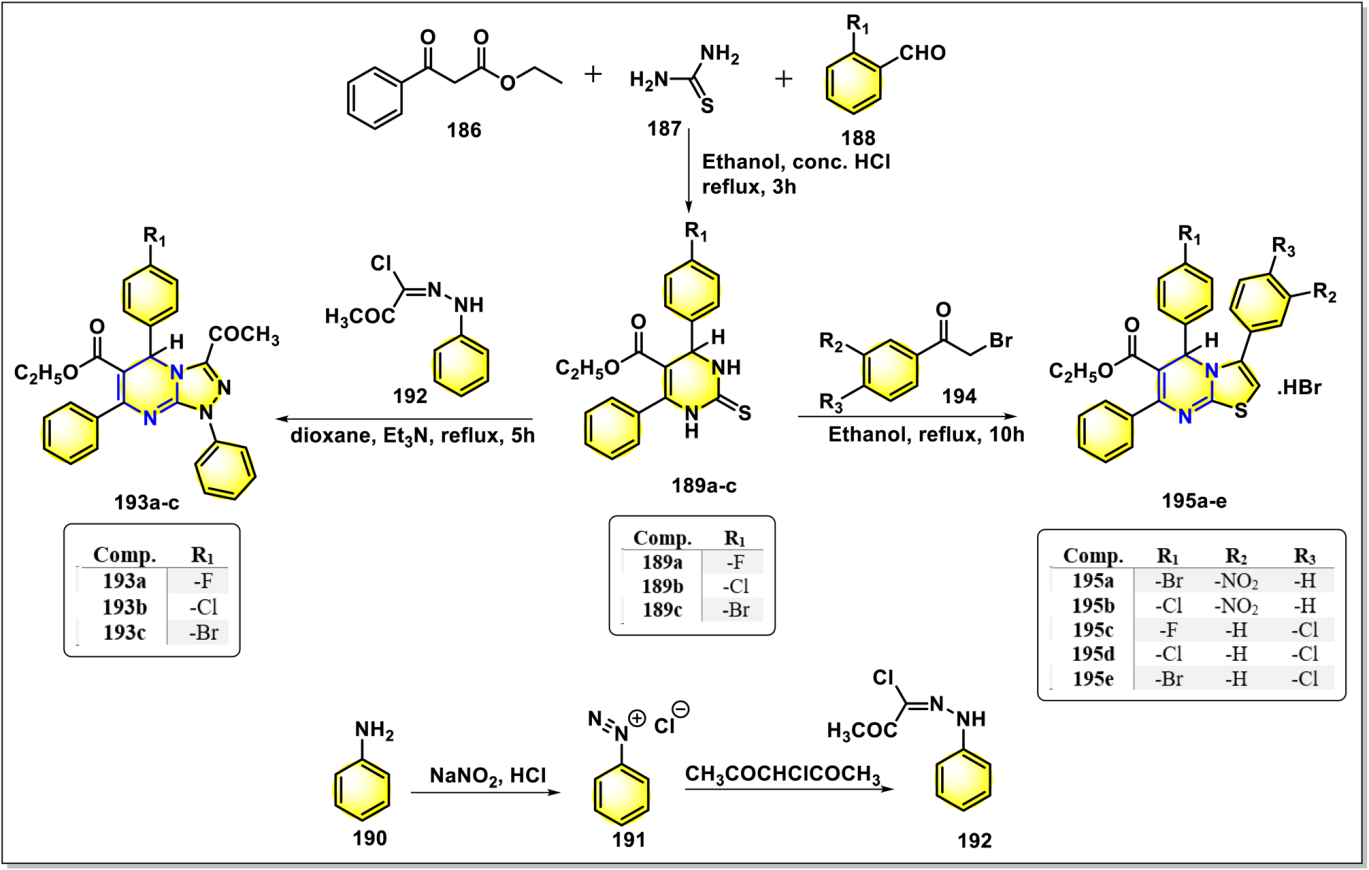
Synthetic strategy for the construction of functionalized triazolopyrimidine (193a–c) and thiazolopyrimidine (195a–e) derivatives,^71^ reproduced from ref. 71 with permission from Elsevier, *Bioorg. Chem.*, 2020, **103**, 104134, Copyright 2020.

The fused pyrimidine derivatives were synthesised through a multicomponent condensation of ethyl benzoyl acetate (186), thiourea (187), and substituted aromatic aldehydes (188) under acidic conditions to generate intermediates (189a–c). These intermediates were further transformed *via* cyclisation and reaction with substituted phenacyl bromides or diazotised species to afford the corresponding triazolopyrimidine (193a–c) and thiazolopyrimidine (195a–e) derivatives (Fig. 53). This study provides unique insights into the efficacy of fused pyrimidine derivatives as anticancer agents.

The amalgamation of thiazole and pyrimidine moieties provide compounds with significant anticancer efficacy, especially against renal cancer cells.

### 4.7 Design, synthesis and antitumor activity of novel thiophene–pyrimidine derivatives as EGFR inhibitors overcoming T790M and L858R/T790M mutations

**Fig. 54:**
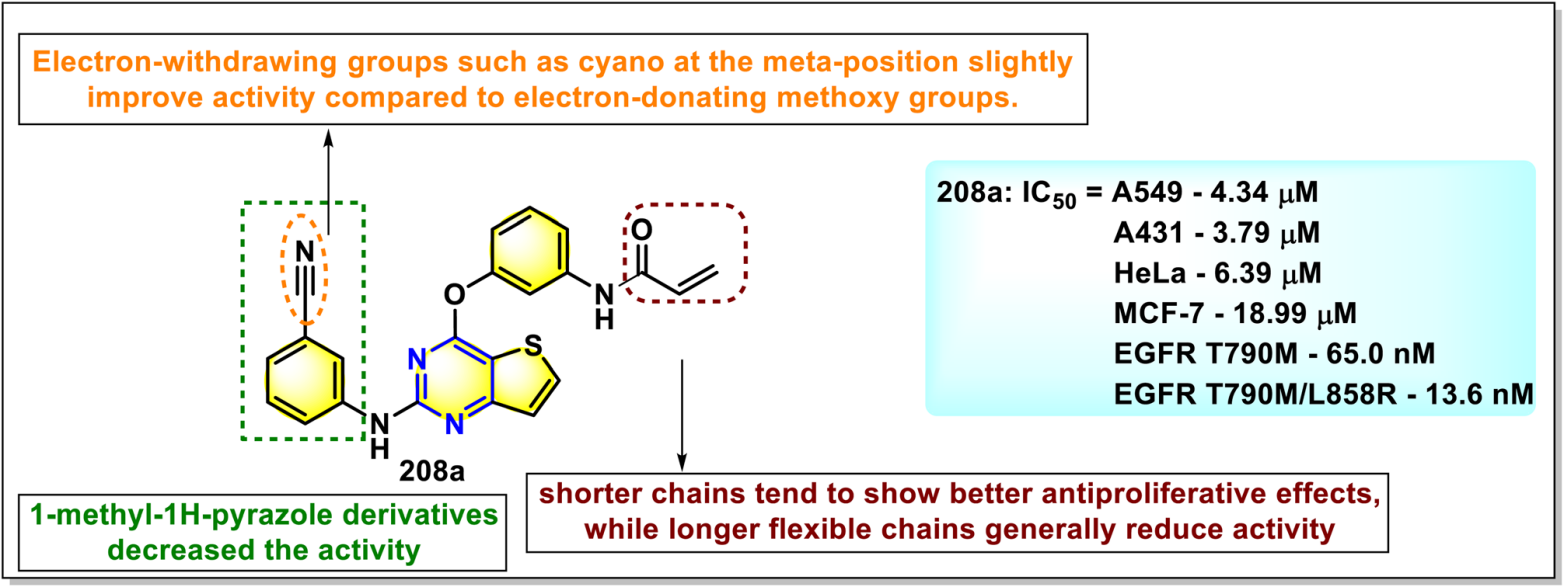
Structure and activity of the most potent thienopyrimidine-based compound synthesised by Xiao Z. *et al.*^64^

The emergence of acquired resistance mutations, particularly T790M, which accounts for approximately 50–60% of clinical resistance to first- and second-generation EGFR inhibitors, necessitated the development of novel scaffolds capable of selectively targeting these resistant mutant forms. Motivated by the structural framework of olmutinib and the well-documented pharmacological versatility of thiophene-pyrimidine hybrids, Xiao *et al.* undertook the systematic design and synthesis of innovative thiophene–pyrimidine derivatives as EGFR inhibitors aimed at T790M and L858R/T790M mutations in non-small cell lung cancer (NSCLC). Five series of compounds (204a–h, 205a–f, 206a–f, 207a–f, 208a–f) derived from the structure of olmutinib, a third-generation EGFR inhibitor was synthesised. The research sought to address the shortcomings of current EGFR inhibitors, including acquired resistance and toxicity. Comprehensive *in vitro* antiproliferative experiments were performed on multiple cancer cell lines, including A549, A431, HeLa, and MCF-7. Enzymatic activity testing, molecular docking investigations and apoptosis assessments were conducted. Compound 208a exhibited exceptional activity against A549, A431, HeLa, and MCF-7 cell lines, with IC_50_ values of 4.34 µM, 3.79 µM, 6.39 µM and 18.11 µM respectively (Fig. 54).

This molecule also exhibited nanomolar-level efficacy against the H1975 cell line (IC_50_ = 699.2 nM). Compound 208a exhibited significant activity against EGFR^T790M^ and EGFR^T790M/L858R^ with IC_50_ values of 65 nM and 13.6 nM respectively. The study indicates that the placement of the methoxy group in anisidine structures markedly influences the compounds' activity, with substitutions at the meta position typically resulting in enhanced potency. The incorporation of a cyano group at the *meta*-position of the benzene ring marginally enhanced overall activity compared to the methoxy group. These findings provide significant guidance for the future development of EGFR inhibitors targeting resistant mutations in NSCLC.^64^

**Fig. 55:**
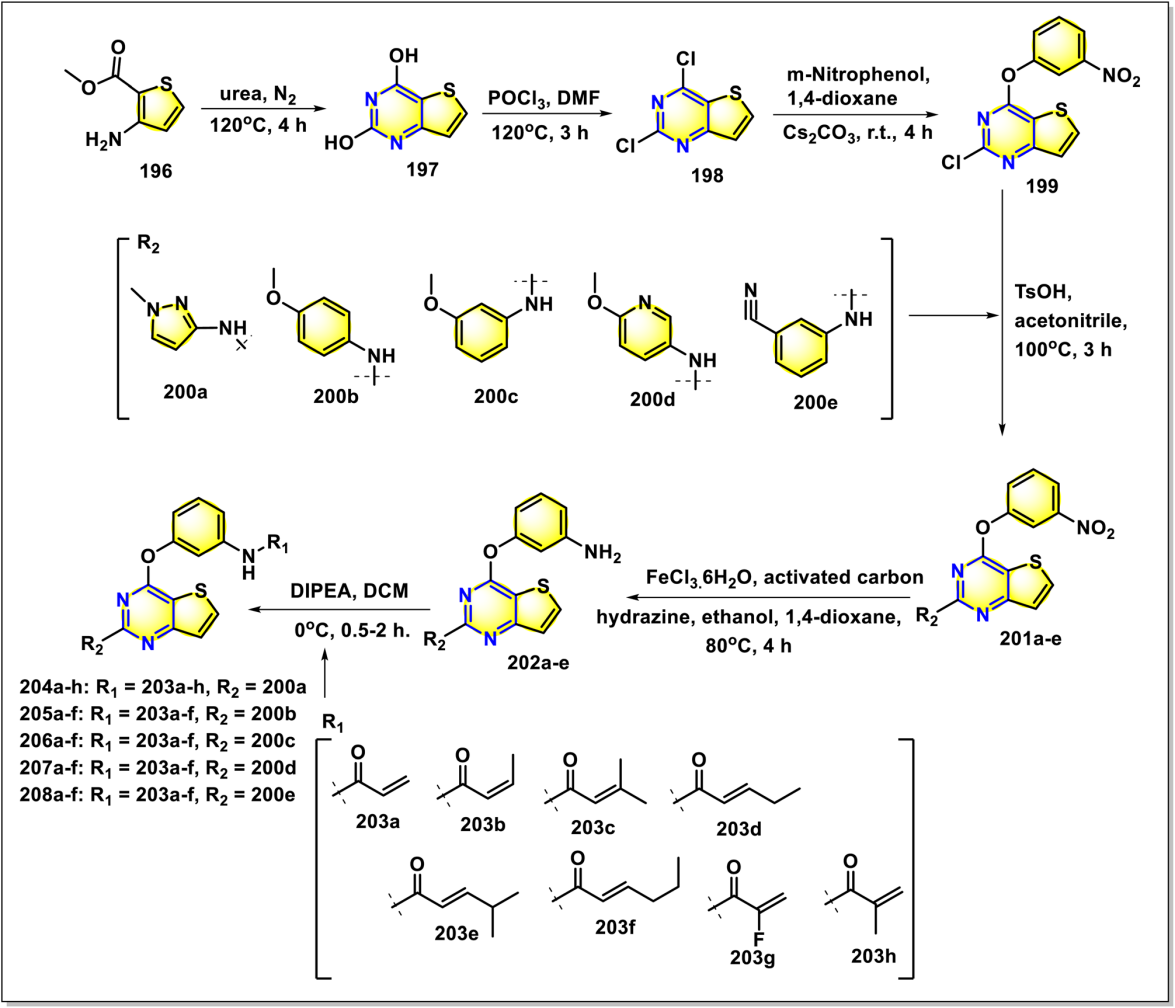
Synthetic route of the novel thiophene–pyrimidine derivatives,^64^ reproduced from ref. 64 with permission from Elsevier, *Eur. J. Med. Chem.*, 2020, **203**, 112511 Copyright 2020.

A multistep synthetic route was employed to access the desired thiophene–pyrimidine derivatives. The synthesis begins with the cyclocondensation of methyl 3-aminothiophene-2-carboxylate (196) with urea to afford intermediate 197. The intermediate was subsequently chlorinated to obtain 198, which then underwent nucleophilic substitution with *m*-nitrophenol, providing compound 199. Further substitution with various amines (200a–e), followed by reduction and final acylation with appropriate acid chlorides, and furnished the desired derivatives (204a–h, 205a–f, 206a–f, 207a–f, and 208a–f) (Fig. 55).

### 4.8 Design, synthesis, and biological evaluation of 2,4-diamino pyrimidine derivatives as potent FAK inhibitors with anti-cancer and anti-angiogenesis activities

**Fig. 56:**
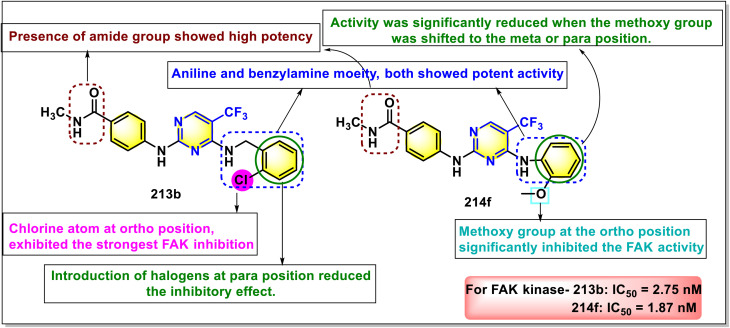
Structural motifs and structure–activity relationship (SAR) of the most potent FAK inhibitors synthesised by Wang S. *et al.*^66^

Focal adhesion kinase (FAK) is a non-receptor tyrosine kinase that plays a crucial role in the proliferation, survival, migration, invasion, and angiogenesis of tumour cells. It has become a significant therapeutic target, especially in pancreatic cancer, where its overexpression is linked to poor prognosis and aggressive disease progression. Due to the limited clinical effectiveness and selectivity issues of current FAK inhibitors, Wang *et al.* aimed to utilize the advantageous 2,4-diaminopyrimidine scaffold to produce new, potent, and selective FAK inhibitors. These inhibitors are designed to simultaneously inhibit tumour growth and angiogenesis, providing a dual-mechanism therapeutic approach for challenging-to-treat cancers. A range of DAPY compounds was produced and assessed for their inhibitory effects on FAK enzyme activity. Compounds 213b and 214f exhibited the most promising results, with IC_50_ values of 2.75 and 1.87 nM, respectively (Fig. 56). These compounds exhibited significant antiproliferative effects against seven human cancer cell lines, including two pancreatic cancer cell lines (PANC-1 and BxPC-3) that overexpress FAK. Subsequent investigations demonstrated that 213b and 214f markedly inhibited colony formation, migration, and invasion of PANC-1 cells in a dose–dependent fashion. Flow cytometry experiments demonstrated that these compounds triggered apoptosis in PANC-1 cells and halted the cell cycle at the G2/M phase. Western blot research demonstrated that 213b and 214f significantly inhibited the FAK/PI3K/Akt signalling pathway and reduced the levels of cyclin D1 and Bcl-2. Furthermore, both drugs exhibited significant anti-angiogenic effects by obstructing the proliferation, migration, and tube formation of human umbilical vein endothelial cells (HUVECs). *In vivo* investigations utilizing zebrafish embryos further validated their anti-angiogenic characteristics.

The study reveals the intriguing dual functionality of compounds 213b and 214f as both anti-cancer and anti-angiogenic drugs. This dual activity may enhance the efficacy of these drugs in cancer treatment by concurrently addressing tumour proliferation and angiogenesis. It is also shown that these compounds possess a favourable selectivity profile, with 213b exhibiting enhanced selectivity for FAK relative to other analogous kinases when compared to 214f. This selectivity may result in minimal side effects in therapeutic applications. The 2,4-diamino pyrimidine framework in the design of potent FAK inhibitors offers crucial structure–activity relationship data for forthcoming pharmaceutical development initiatives in this domain.^66^

**Fig. 57:**
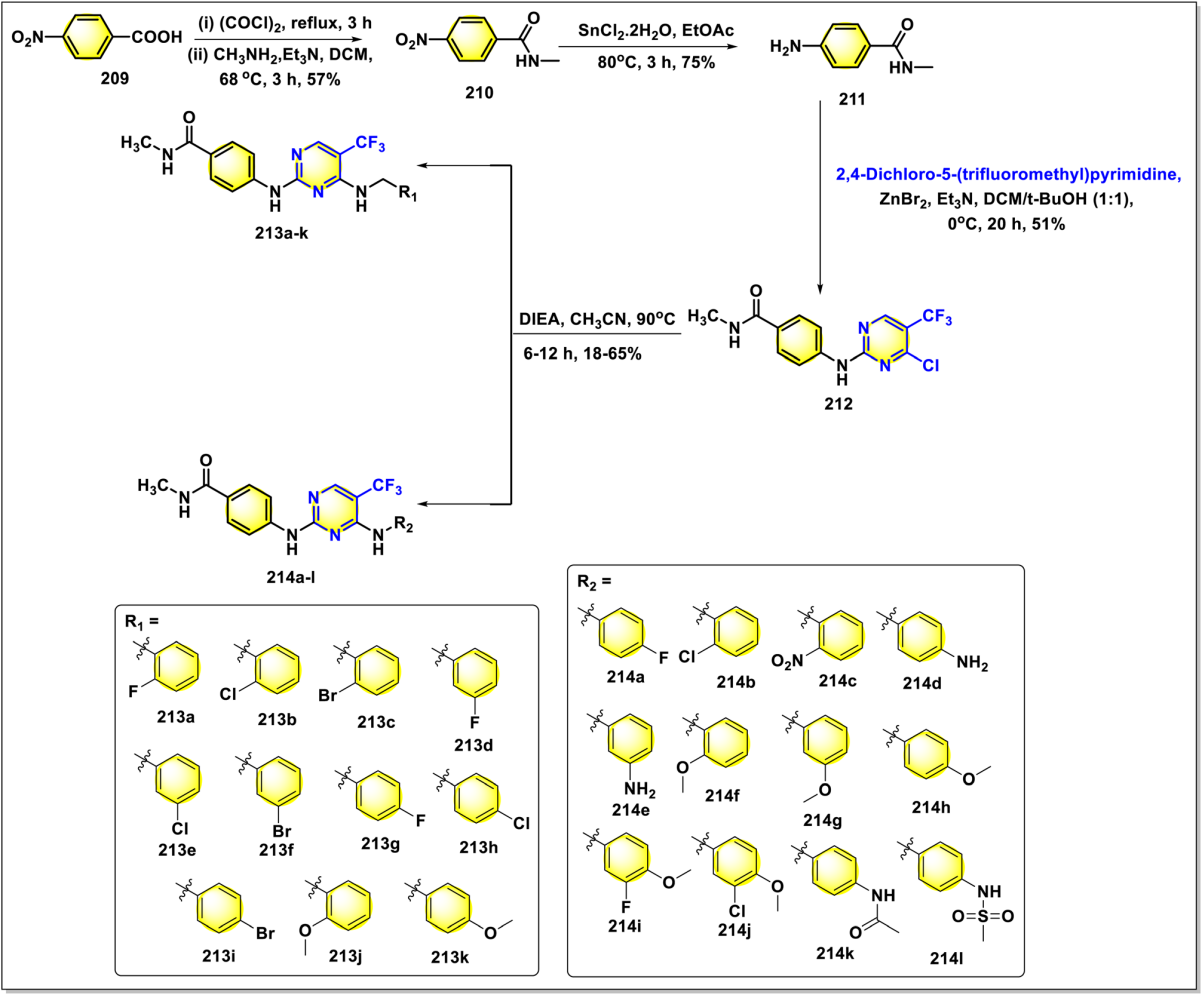
Synthetic strategy for the preparation of aminopyrimidine derivatives 213a–k and 214a–l,^66^ reproduced from ref. 66 with permission from Elsevier, *Eur. J. Med. Chem.*, 2021, **222**, 113573, Copyright 2021.

The DAPY derivatives were synthesised *via* a multistep sequence beginning with the conversion of 4-nitrobenzoic acid (209) to the corresponding acid chloride using oxalyl chloride, followed by amide formation to afford intermediate 210. Subsequent reduction of the nitro group yielded aniline intermediate 211, which underwent nucleophilic aromatic substitution with 2,4-dichloro-5-(trifluoromethyl)pyrimidine to generate key intermediate 212. Final diversification was achieved through coupling of 212 with substituted benzylamines or anilines under basic conditions, affording the desired compounds (213a–k and 214a–l) (Fig. 57).^66^

### 4.9 Design, synthesis and antitumor activity evaluation of trifluoromethyl-substituted pyrimidine derivatives

**Fig. 58:**
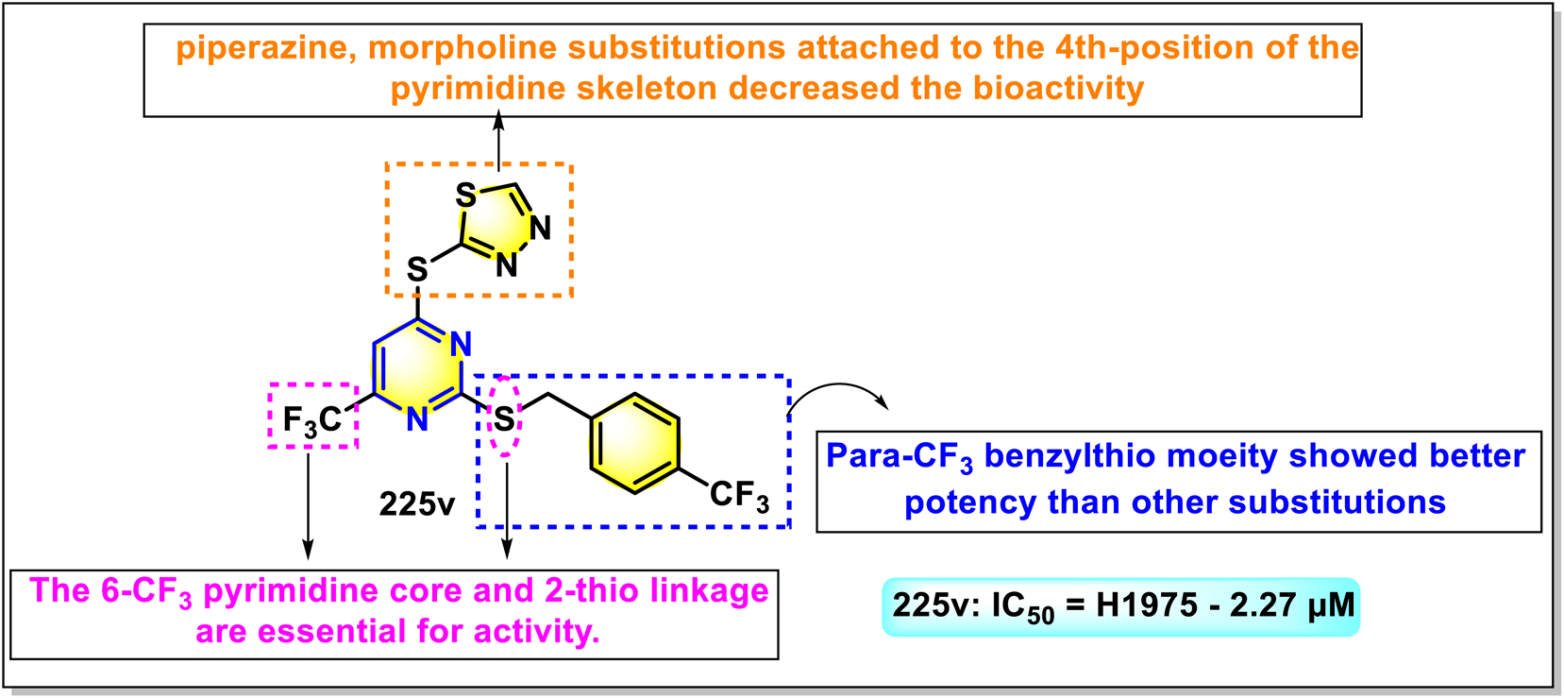
Structure and biological activity of the most potent compound synthesised by Liu *et al.*^67^

Despite the remarkable clinical success of first- and second-generation EGFR inhibitors in NSCLC treatment, the inevitable emergence of resistance mutations and the inherent limitations of existing pyrimidine-based anticancer agents in terms of potency and selectivity underscored the pressing need for structurally innovative scaffolds with improved pharmacological profiles. Capitalizing on the well-established role of the trifluoromethyl group as a critical bioisostere capable of enhancing metabolic stability, membrane permeability, and target binding affinity, Liu *et al.* (2021) strategically incorporated this electron-withdrawing moiety into a novel pyrimidine framework to develop potent anticancer candidates with improved efficacy against four human tumour cell lines: PC-3, MGC-803, MCF-7, and H1975. Compound 225v exhibited significant anti-proliferative activity against H1975 cells (IC_50_ = 2.27 µM), exceeding the positive control 5-FU (IC_50_ = 9.37 µM). Multiple studies were performed to assess the mechanisms of action of 225v (Fig. 58). The methods employed comprised of colony formation assays, cell cycle analysis, scratch assays, DAPI staining, flow cytometry for apoptosis assessment, and western blot analysis. The findings demonstrated that compound 225v greatly inhibited the migration and colony formation of H1975 cells, induced cell cycle arrest at the G2/M phase and facilitated apoptosis *via* the activation of the intrinsic apoptotic pathway.

Molecular docking analysis indicated that compound 225v may effectively attach to the EGFR pocket. The introduction of a trifluoromethyl group into the pyrimidine framework appears to be a promising approach for enhancing anticancer activity. The comprehensive evaluation of mechanism of action of compound 225v offers a thorough grasp of its potential as an anticancer agent, establishing a solid basis for future drug development initiatives in this field.^67^

**Fig. 59:**
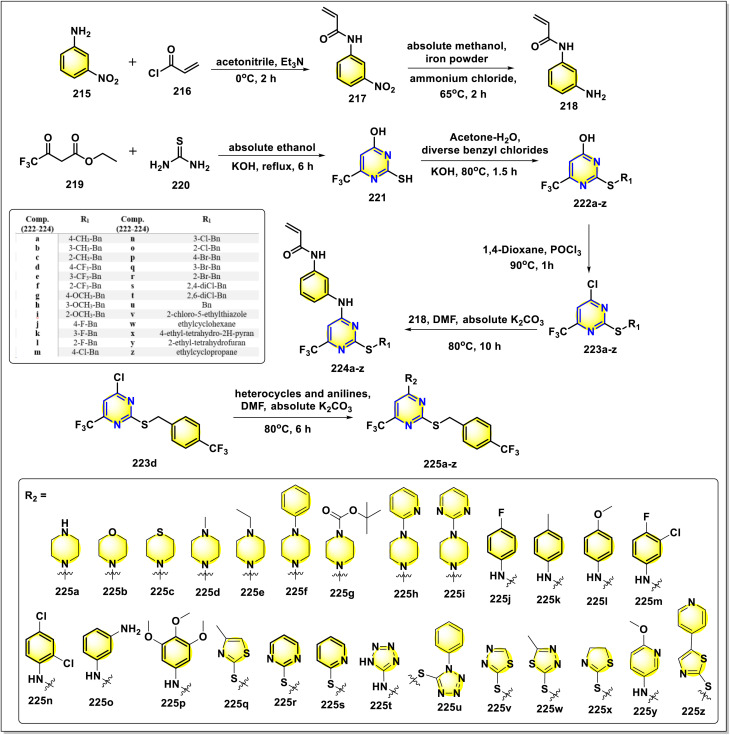
Synthesis of the target trifluoromethyl-substituted pyrimidine derivatives,^67^ reproduced from ref. 67 with permission from Elsevier, *Bioorg. Med. Chem. Lett.*, 2021, **51**, 128268, Copyright 2021.

As illustrated in Fig. 59, a convergent synthetic approach was employed for the construction of the target pyrimidine derivatives (224a–z and 225a–z), integrating two key building blocks. Initially, amide formation between 3-nitroaniline (215) and acryloyl chloride (216) afforded intermediate 217, which was subsequently reduced to yield the corresponding amine intermediate 218. In parallel, cyclocondensation of ethyl 4,4,4-trifluoro-3-oxobutanoate (219) with thiourea (220) generated the pyrimidine core (221), followed by *S*-alkylation with substituted benzyl chlorides to furnish intermediates (222a–z). Further activation *via* POCl_3_-mediated chlorination produced intermediates (223a–z), which upon coupling with amine 218 or various heterocycles delivered the final derivatives (224a–z and 225a–z), enabling efficient structural diversification.

### 4.10 Design, synthesis and anti-tumour evaluation of novel pyrimidine and quinazoline analogues

**Fig. 60:**
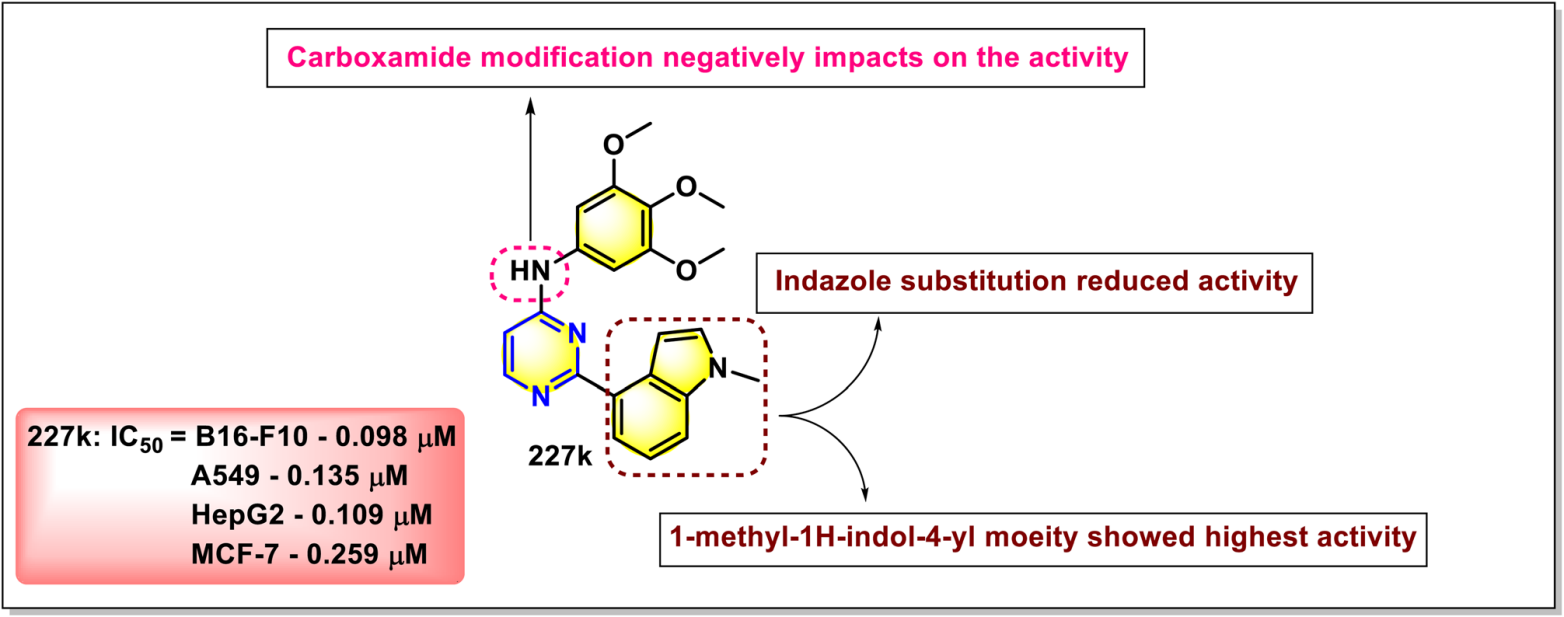
Structure and inhibitory profile of the most potent compound synthesised by Lin R. J. *et al.*^72^

Microtubule-targeting agents, while proven effective in clinical settings, still encounter major therapeutic hurdles such as acquired drug resistance due to P-glycoprotein efflux pumps, dose-limiting toxicity, and lack of selectivity. These issues underscore the pressing need for new agents that can interact with alternative tubulin binding sites more effectively and with better tolerability. Motivated by the distinct pharmacological benefits of the colchicine binding site, which serves as a unique and resistance-evading target, Lin R. J. *et al.* (2025) pursued the rational design and synthesis of hybrid pyrimidine and quinazoline frameworks. Their goal was to synthesis potent inhibitors of microtubule polymerization that could address the limitations of current clinical agents while maintaining strong antitumor activity *in vivo* with minimal systemic toxicity. A variety of compounds were synthesised derived from the structure of colchicine, a recognized microtubule inhibitor. They produced many derivatives and evaluated their antiproliferative efficacy against various cancer cell lines. Compound 227k had substantial anti-tumour efficacy in both *in vitro* and *in vivo* settings. It demonstrated significant reduction of cancer cell proliferation, against B16-F10, A549, HepG2, and MCF-7 cell lines with an IC_50_ value of 0.098 µM, 0.135 µM, 0.109 µM, and 0.259 µM, respectively. Mechanistic investigations demonstrated that 227k functions by obstructing microtubule protein polymerization, resulting in cell cycle arrest at G2/M phase and triggering apoptosis (Fig. 60).

The compound demonstrated the capacity to inhibit tumour cell migration and had considerable anti-tumour activity in a murine melanoma model without inducing notable toxicity. This work reveals the potential of compound 227k to address certain shortcomings of current microtubule-targeting pharmaceuticals. In contrast to paclitaxel and vinca alkaloids, which may be influenced by P-glycoprotein-mediated resistance, drugs that engage with the colchicine binding site may demonstrate reduced resistance. The combination of these properties, along with its significant anti-tumour efficacy and minimal toxicity, establishes 227k as a potential molecule for the creation of novel anti-cancer agents targeting microtubules.^72^

**Fig. 61:**
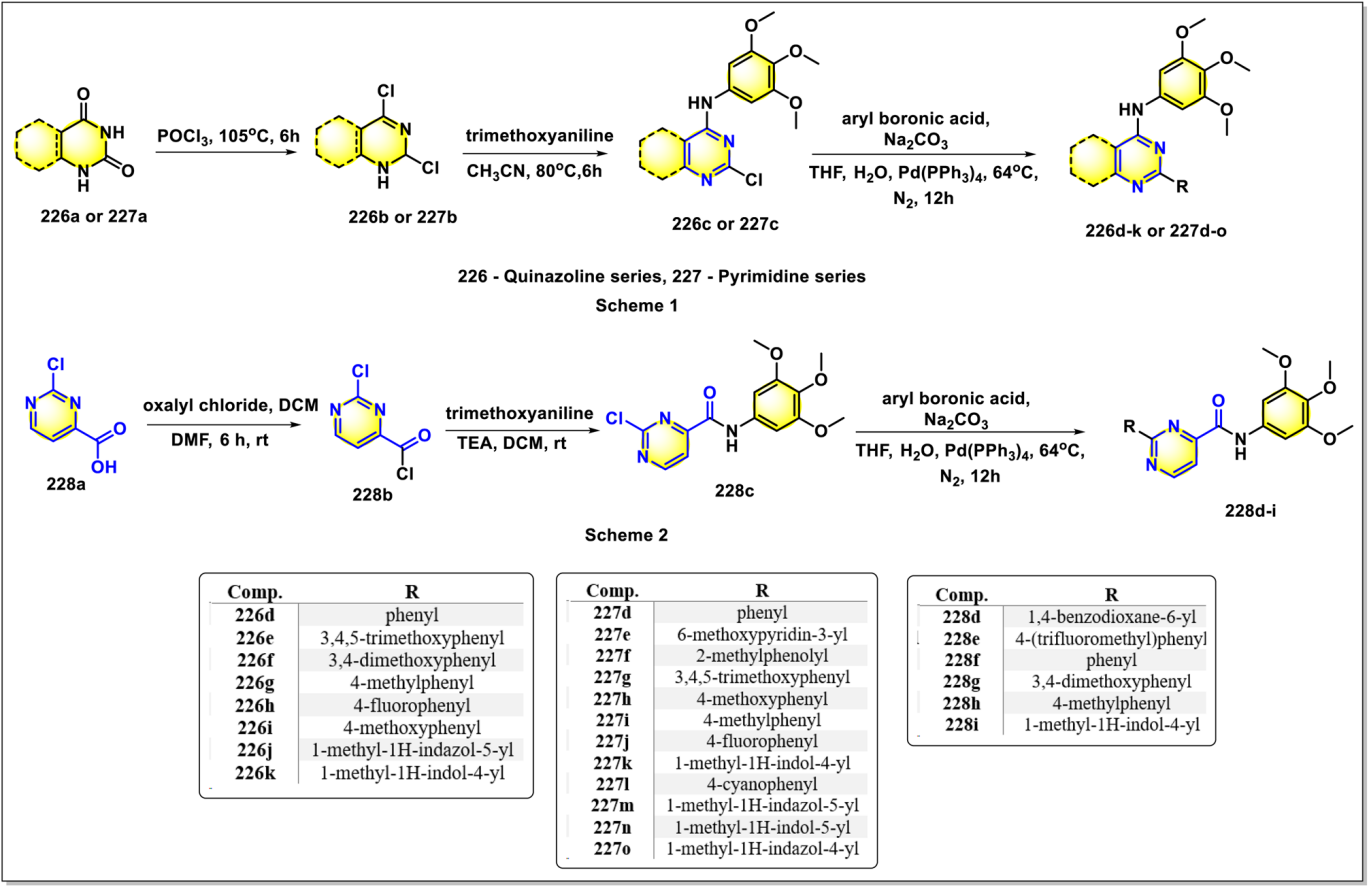
Synthetic strategy for preparation of novel pyrimidine and quinazoline analogues 226d–k, 227d–o and 228d–i,^72^ reproduced from ref. 72 with permission from Elsevier, *Eur. J. Med. Chem.*, 2025, **282**, 117057, Copyright 2024.

The target quinazoline and pyrimidine derivatives were synthesised *via* complementary synthetic routes involving chlorination, regioselective substitution, and Pd-catalysed cross-coupling reactions. As can be seen in Fig. 61, quinazoline intermediates were generated by POCl_3_-mediated chlorination of quinazolinedione precursors (226a), followed by selective nucleophilic substitution with trimethoxyaniline and subsequent Suzuki–Miyaura coupling with aryl boronic acids to furnish compounds (226d–k).

A similar strategy was applied for pyrimidine derivatives (227d–o), starting from uracil-derived intermediates, where chlorination followed by nucleophilic substitution and Suzuki coupling enabled efficient structural diversification. In scheme 2, acid chloride formation from 2-chloropyrimidine-4-carboxylic acid (228a) was followed by amide coupling with trimethoxyaniline and final Suzuki–Miyaura cross-coupling to afford compounds (228d–i).^72^

## 5 Drug development challenges and AI/ML in drug discovery

In the clinical drug development, an ideal drug candidate should exhibit both high potency and target specificity. The drug candidate should be effectively modulating the molecular target with minimal off-target effects. Additionally, it must demonstrate high drug exposure in disease-targeted tissues to achieve adequate efficacy at an optimal dose and limit drug exposure in healthy tissues to avoid toxicity, even at high doses.^73^ The journey from laboratory bench to clinical approval represents one of the most challenging parts in drug development. Over the past decades, it has been shown that almost 90% of drug candidates have failed during clinical development or regulatory approval primarily due to lack of clinical efficacy, unacceptable toxicity, poor drug-like properties.^74^

To achieve successful drug development, it is requires moving beyond single-parameter optimization (potency) toward a comprehensive multi-parameter optimization strategy that integrates potency with ADMET properties, off-target effects, and clinical prediction. In this context, Computer-Aided Drug Design (CADD) is a revolutionary part in modern drug discovery, operating at the interface of complex biological systems and advanced computational methodologies. Quantitative structure–activity relationship (QSAR) modeling, *in silico* ADMET property prediction, mechanistic pharmacokinetic simulations, and multi-criteria decision analysis represent promising advances in drug discovery. The integration of artificial intelligence (AI) and machine learning (ML) with CADD is reshaping the drug discovery rates. This integrated platform improves the efficiency and success rate of therapeutic development.^75^

AI-driven methodologies are transforming drug discovery and development by enabling simultaneous optimization of multiple candidate parameters and improving therapeutic efficacy through targeted approaches. These strategies accelerate high-throughput screening, support structure-based molecular design to enhance target binding and physicochemical properties, and enable the repurposing of existing drugs by exploring new parameters. AI has gained great significance in comprehensive assessment of safety, efficacy, toxicity, and pharmacological responses within complex biological systems and it also supports early prediction of clinical implementation in humanized models.^76^ The emergence of advanced data-driven AI models in ADMET and toxicity prediction has significantly enhanced the efficiency of preclinical drug development.^77^

Artificial intelligence is reshaping clinical trial design and development in the areas of patient stratification and dose optimization. In clinical development, AI has also demonstrated significant impact on patient selection, thereby minimizing bias and optimizing trial design. So, these advancements can be beneficial for clinical studies. Digital twins (DTs) have the transformative potential throughout the drug discovery and development cycle. Recent developments in DT technology are reshaping the design of clinical trials using complex modeling frameworks that overcome major challenges in patient recruitment, retention and outcome prediction. In early-phase clinical trials, especially oncology and rare diseases DTs may be utilized to simulate and predict drug safety, pharmacokinetic (PK) profiles, and early pharmacodynamic responses within small groups of healthy volunteers or patients.^78,79^ AI platform like Trial pathfinder, an AI based platform that analyze electronic health records (EHR) to optimize the trial inclusion criteria. This system can evaluate the overall survival risk ration and allow the stimulation of virtual trials across demographic subgroups. It also able to identify the more efficient enrolment strategies, that potentially reducing the required sample size by 25–40 percentage while maintain the statistical power.^77,80^ In addition, Hassanzadeh *et al.* proposed a machine learning framework that capable to match the patients automatically to clinical trial based on predefine trial eligibility criteria, thereby enhance the efficiency of the recruitment process.^80,81^ Machine learning-driven patient-trial matching approaches in oncology trails have accelerated enrollment (35%) and improved survival outcomes.^77^ Graph neural networks (GNNs) are used to model pharmacokinetic parameters for dose optimization. These models have the ability of predicting key important parameters, including drug half-life, facilitating more optimized dosing regimens, though additional clinical validation is necessary to fully realize this potential.^77,82^

To increase the success rate of clinical drug development, effective drug optimization requires a balanced integration structure–activity relationships (SAR) and structure–tissue relationships (STR) in selecting drug candidates for clinical trial.^73,83^ To improve the drug optimization process, Duxin Sun *et.al.*, introduced the structure–tissue exposure/selectivity–activity relationship (STAR) framework and which classify drug candidates into four distinct categories based on three important factors: drug potency/specificity (high or low), drug tissue exposure/selectivity (high or low) and required dose for balancing clinical efficacy/toxicity (high or low).^84^ In 2024, Duxin Sun *et al.*, have proposed a “STAR-guided ML system” (structure−tissue/cell selectivity–activity relationship) to enhance success rate and efficacy by addressing three overlooked interdependent factors^1^ potency and specificity to the on-off-targets determining efficacy in tumors at clinical doses^2^ on/off-target-driven tissue or cell selectivity which influences adverse effects in the normal organs at clinical doses,^3^ optimal clinical doses balancing efficacy and safety as determined by potency-specificity (PS) and tissue/cell selectivity (TS). In order to design and select the best drug, these models can directly predict the clinical dose, efficacy, and safety. STAR-guided ML approaches have the potential to enhance cancer drug development efficiency and may boost overall success rate seven-fold from approximately 5% to 35% by achieving 70% success across the phases I,II,III in clinical trials. Collectively these advancements offer a transformative framework to enhance the efficiency cancer drug development by reducing time and cost.^85^

## 6 Conclusion and future perspective

This review surveys the synthesis, structural variety, and anticancer effectiveness of imidazole, indole, and pyrimidine scaffolds based on literature from 2020 to 2025. It confirms their importance in the development of modern cancer drugs. These nitrogen-containing compounds are often used as core structures or in hybrid forms. They have demonstrated strong cytotoxicity across various cancer cell lines, often outperforming drugs such as doxorubicin, cisplatin, and 5-fluorouracil, with IC_50_ values in the sub-micromolar to nanomolar range. Important structural changes, such as ether linkages and long-chain extensions on imidazoles, fused or substituted indole structures, and pyrimidine hybrids with kinase-mimicking parts, have been crucial in improving their potency and selectivity. Many derivatives kill tumour cells effectively while sparing normal fibroblasts or epithelial cells.

Mechanistically, the scaffolds indole, pyrimidine and imidazole affect key oncogenic pathways triggering apoptosis, inhibiting multitarget kinases, suppressing angiogenesis and exhibiting cytotoxic effects. They often perform better than standard drugs in growth assays and selectivity tests. For example, specific imidazole-indole hybrids have reached IC_50_ values in the low nanomolar range against breast and lung cancer models. Pyrimidine-based kinase inhibitors have shown significant effects against angiogenesis in endothelial co-culture systems. Their versatile binding ability allows them to interact with multiple targets, which reduces the likelihood of developing resistance compared to single-target drugs.

A recurring challenge is the lack of complete mechanistic profiling, limited *in vivo* pharmacokinetics data, and inadequate ADMET optimization. These issues hinder clinical progress, despite their strong promise in lab tests. While literature mainly focuses on cytotoxicity, further studies on target engagement, resistance mechanisms, and interactions with the tumour microenvironment are necessary for validation.

Future efforts should focus on structure–activity relationships to refine the compounds. Patterns show that electron-rich additions on indole nitrogens, optimal *N*-functionalization on imidazoles, and C-4/C-6 appendages on pyrimidines consistently increase activity. In contrast, sterically bulky or highly polar variants lower effectiveness. Computational tools like docking, QSAR, and pharmacophore modelling will facilitate the transition from initial findings to lead compounds by predicting target affinity and drug-like qualities. Combining these compounds with complementary agents, such as redox modulators or PROTACs, and using combination treatments with immunotherapies or established drugs may help combat resistant cancer types. Meanwhile, eco-friendly synthetic methods, such as multicomponent reactions and biocatalysis, along with thorough *in vivo* toxicity assessments, will help bridge the gap to clinical application.

In conclusion, imidazole, indole, and pyrimidine scaffolds form a strong base for developing new anticancer agents. This review highlights their accessible synthesis, versatility in targeting, and therapeutic potential. Continued emphasis on mechanistic understanding, multi-parameter optimization, and translational studies is essential for turning these leads into effective clinical drugs. This work will ultimately help address the global cancer crisis.

## Author contributions

Maithreyi Govindarajan: writing – original draft, conceptualization, investigation, data curation. Lalmohan Maji: writing – original draft, investigation, visualization, formal analysis. Mannathan Subramaniyan: writing – review & editing; Kathiravan Muthu Kumaradoss: writing – review & editing, validation, resources, supervision, conceptualization. Arpan Chowdhury: writing – review & editing, Seetharama D. Jois: conceptualization, writing – review & editing, Baburaj Baskar: writing – review & editing, supervision, resources, project administration, conceptualization.

## Conflicts of interest

The authors declare that they have no known competing financial interests or personal relationships that could have appeared to influence the work reported in this paper.

## Abbreviations

DNA: Deoxyribonucleic acid
HIV: Human immunodeficiency virus
ATP: Adenosine triphosphate
ADMET: Absorption, distribution, metabolism, excretion, toxicity
QSAR: Quantitative structure–activity relationship
DMF: *N*,*N*-Dimethylformamide
EGFR: Epidermal growth factor receptor
STING: Stimulator of interferon genes
TMS: Tetramethylsilane
HPLC: High-performance liquid chromatography
NMR: Nuclear magnetic resonance
HRMS: High-resolution mass spectrometry
FT-IR: Fourier transform infrared spectroscopy
MTT assay: 3-(4,5-Dimethylthiazol-2-yl)-2,5-diphenyltetrazolium bromide assay
DFT: Density functional theory
DIEA: *N*,*N*-Diisopropylethylamine
nM: Nanomolar
µM: Micromolar

## Data Availability

No primary research results, software or code have been included and no new data were generated or analysed as part of this review.

## References

[cit1] Sarkar S., Horn G., Moulton K., Oza A., Byler S., Kokolus S., Longacre M. (2013). Int. J. Mol. Sci..

[cit2] Alfarouk K. O., Stock C.-M., Taylor S., Walsh M., Muddathir A. K., Verduzco D., Bashir A. H., Mohammed O. Y., Elhassan G. O., Harguindey S., Reshkin S. J., Ibrahim M. E., Rauch C. (2015). Cancer Cell Int..

[cit3] Thun M. J., DeLancey J. O., Center M. M., Jemal A., Ward E. M. (2010). Carcinogenesis.

[cit4] Mehboob R., Pak J. (2025). Health Sci..

[cit5] Jampilek J. (2019). Molecules.

[cit6] Kumar A., Singh A. K., Singh H., Vijayan V., Kumar D., Naik J., Thareja S., Yadav J. P., Pathak P., Grishina M., Verma A., Khalilullah H., Jaremko M., Emwas A.-H., Kumar P. (2023). Pharmaceuticals.

[cit7] Damanpreet K. L., Rajwinder K., Rashmi A., Balraj S., Sandeep A. (2020). Anti-Cancer Agents Med. Chem..

[cit8] Murtazaeva Z., Nasrullaev A., Buronov A., Gaybullaev S., Nie L., Khushnazarov S. N. Z., Turgunov D., Kuryazov R., Zhao J., Bozorov K. (2025). Molecules.

[cit9] Yadav G., Jain R. (2025). Eur. J. Med. Chem..

[cit10] Zhang L., Peng X.-M., Damu G. L. V., Geng R.-X., Zhou C.-H. (2014). Med. Res. Rev..

[cit11] Burlacu A. P., Drăgan M., Oniga O., Matei M. N., Oniga I., Lisă E.-L., Stefan C. S., Dragostin O. M. (2026). Molecules.

[cit12] Kumar R. (2024). Asian J. Res. Chem..

[cit13] Verma A., Joshi S., Singh D. (2013). J. Chem..

[cit14] Shalini K., Sharma P. K., Kumar N. (2010). Der Chem. Sin..

[cit15] Gujjarappa R. , Kabi A. K., Sravani S., Garg A., Vodnala N., Tyagi U., Kaldhi D., Velayutham R., Singh V., Gupta S., Nanostructured Biomaterials: Basic Structures and Applications, ed. B. P. Swain, Springer Singapore, Singapore, 2022, pp. 135–227

[cit16] Rasajna G., Pharma J. (2025). Insights Res..

[cit17] Raphael S. M. M., Beatriz M. B. A., Gabriel T. A. P., Searitha C. R., Tereza M. M. M., Lucas A. S. D., Camille C. C., Aline de A. P., Flaviana R. F. D., Patricia D. F., Claudia C. A. (2025). Chem. Rec..

[cit18] Dhuguru J., Skouta R. (2020). Molecules.

[cit19] Mo X., Rao D. P., Kaur K., Hassan R., Abdel-Samea A. S., Farhan S. M., Bräse S., Hashem H. (2024). Molecules.

[cit20] Vinod A., Mouli H. M. C., Jana A., Peraman R. (2024). Med. Chem. Res..

[cit21] Prasanta S. T., Mukherjee S. O. (2018). Mini-Rev. Med. Chem..

[cit22] Kumar S. (2020). Ritika. Future J. Pharm. Sci..

[cit23] Sharma V., Kumar P., Pathak D. (2010). J. Heterocycl. Chem..

[cit24] Neha D., Kamalpreet K., Avadh B., Vikas J. (2021). Anti-Cancer Agents Med. Chem..

[cit25] Kamalpreet K., Vikas J. (2019). Anti-Cancer Agents Med. Chem..

[cit26] Teli G., Pal R., Maji L., Matada G. S. P., Sengupta S. (2024). J. Biomol. Struct. Dyn..

[cit27] Kumar S., Narasimhan B. (2018). Chem. Cent. J..

[cit28] Nadar S., Khan T. (2022). Chem. Biol. Drug Des..

[cit29] Maria Assunta C., Daniela I., Roberto R., Salvatore V. G., Laura L. (2019). Curr. Med. Chem..

[cit30] Rani J., Kumar S., Saini M., Mundlia J., Verma P. K. (2016). Res. Chem. Intermed..

[cit31] Ramalakshmi N., Helina Navis Anthoni S., Amuthalakshmi S., Arunkumar S. (2023). Med. Chem..

[cit32] Ravindar L., Hasbullah S. A., Rakesh K. P., Raheem S., Ismail N., Ling L. Y., Hassan N. I. (2024). Bioorg. Med. Chem. Lett..

[cit33] Vyas A., Sahu B., Pathania S., Nandi N. K., Chauhan G., Asati V., Kumar B. (2023). J. Heterocycl. Chem..

[cit34] Abbas N., Swamy P. M. G., Dhiwar P., Patel S., Giles D. (2021). Pharm. Chem. J..

[cit35] Sharma S., Babu M. A., Kumar R., Singh T. G., Dwivedi A. R., Ahmad G., Goel K. K., Kumar B B. (2025). Mol. Diversity.

[cit36] Pravin N. J., Kavalapure R. S., Alegaon S. G., Gharge S., Ranade S. D. (2025). Bioorg. Chem..

[cit37] Faris M., Bostancı H. E., Özcan İ., Öztürk M., Koçyiğit Ü. M., Erdoğan T., Tahtaci H. (2024). ACS Omega.

[cit38] Sharma P., LaRosa C., Antwi J., Govindarajan R., Werbovetz K. A. (2021). Molecules.

[cit39] Li N., Xu M., Zhang L., Lei Z., Chen C., Zhang T., Chen L., Sun J. (2022). J. Med. Chem..

[cit40] Emami L., Zare F., Khabnadideh S., Rezaei Z., Sabahi Z., Gheshlaghi S. Z., Behrouz M., Emami M., Ghobadi Z., Ardekani S. M., Barzegar F., Ebrahimi A., Sabet R. (2024). BMC Chem..

[cit41] Chitrakar R., Rawat D., Sistla R., Vadithe L. N., Subbarayappa A. (2021). Bioorg. Med. Chem. Lett..

[cit42] Vootukoori S. R., Nimmareddy R. R., A V. R., Dodda S., Leleti K. R. (2025). ChemistrySelect.

[cit43] Hou H., Zhou J., Sui Q., Zhang C., Su Z., Cui R., Shan B., Xu P., Chen Z., Jiang B., Li M., Zhang K., Wang Y., Ma N., Teng D., Zheng M., Zhang S. (2025). J. Med. Chem..

[cit44] Gryniukova A., Borysko P., Myziuk I., Alieksieieva D., Hodyna D., Semenyuta I., Kovalishyn V., Metelytsia L., Rogalsky S., Tcherniuk S. (2024). Mol. Diversity.

[cit45] Diaconu D., Antoci V., Mangalagiu V., Amariucai-Mantu D., Mangalagiu I. I. (2022). Sci. Rep..

[cit46] Datta P., Sardar D., Sinha C., Manna A., Chatterjee M. (2022). Asian J. Chem..

[cit47] Rasal N. K., Sonawane R. B., Jagtap S. V. (2020). Bioorg. Chem..

[cit48] Mehra A., Sharma V., Verma A., Venugopal S., Mittal A., Singh G., Kaur B. (2022). ChemistrySelect.

[cit49] Wan Y., Li Y., Yan C., Yan M., Tang Z. (2019). Eur. J. Med. Chem..

[cit50] Momen R. F. M., Mai E. S., Taha F. S. A., Gamal El-Din A.-R. (2024). Lett. Drug Des. Discovery.

[cit51] Debnath B., Nandi B., Paul S., Manna S., Maity A., Bandyopadhyay K., Panda S., Khan S. A., Nath R., Akhtar M. J. (2025). Med. Drug Discovery.

[cit52] Pecnard S., Liu X., Provot O., Retailleau P., Tran C., Hamze A. (2024). Org. Chem. Front..

[cit53] Song Y., Feng S., Feng J., Dong J., Yang K., Liu Z., Qiao X. (2020). Eur. J. Med. Chem..

[cit54] Kryshchyshyn-Dylevych A., Radko L., Finiuk N., Garazd M., Kashchak N., Posyniak A., Niemczuk K., Stoika R., Lesyk R. (2021). Bioorg. Med. Chem..

[cit55] Yadav A., Singh V. K., Kumar R., Yadav V., Kushwaha A. K., Kumar Rana V., Kumar A., Prasad V. (2025). J. Mol. Struct..

[cit56] Taherkhani A. M., Sayahi M. H., Hassani B., Dastyafteh N., M- Khanaposhtani M., Rafiei E., Meshkani M., Safapoor S., Tehrani M. M., Larijani B., Mahdavi M., Firuzi O. (2025). J. Mol. Struct..

[cit57] Shenghui W., Yingjuan G., Xiujuan L., Xinying Y., Guangxi Y., Yinru L., Yanbing Z., Jian S., Wen L., Saiyang Z. (2021). Chin. J. Org. Chem..

[cit58] Islam M. S., Ali M., Al-Majid A. M., Alamary A. S., Alshahrani S., Yousuf S., Choudhary M. I., Barakat A. (2021). Molecules.

[cit59] Rzepka Z., Bębenek E., Chrobak E., Wrześniok D. (2022). Pharmaceutics.

[cit60] Zidar N., Secci D., Tomašič T., Mašič L. P., Kikelj D., Passarella D., Garcia Argaez A. N., Hyeraci M., Via L. D. (2020). ACS Med. Chem. Lett..

[cit61] Ganga Reddy G., Pramod Kumar D., Ramana Reddy C. V. (2020). Lett. Drug Des. Discovery.

[cit62] Mai M. Z., Osama M. E., Salwa E.-M., Rasha A. H. (2025). Curr. Pharm. Des..

[cit63] Yadav U. P., Yaseen M., Singh S., Babu M. A., Bhat M. A., Kumar R., Tyagi Y., Ullah I., Huang Y. (2025). Bioorg. Chem..

[cit64] Xiao Z., Zhou Z., Chu C., Zhang Q., Zhou L., Yang Z., Li X., Yu L., Zheng P., Xu S., Zhu W. (2020). Eur. J. Med. Chem..

[cit65] Kim Y., Lee K. W., Yeom H., Kim M., Lee Y. K., Lee J.-Y., Hwang J. Y., Min Y., Ryu D. H., Lee C. H., Cho S. Y. (2021). Bull. Korean Chem. Soc..

[cit66] Wang S., Zhang R.-H., Zhang H., Wang Y.-C., Yang D., Zhao Y.-L., Yan G. Y., Xu G. B., Guan H. Y., Zhou Y. H., Cui D. B., Liu T., Li Y. J., Liao S. G., Zhou M. (2021). Eur. J. Med. Chem..

[cit67] Liu L., Wang Z., Gao C., Dai H., Si X., Zhang Y., Meng Y., Zheng J., Ke Y., Liu H., Zhang Q. (2021). Bioorg. Med. Chem. Lett..

[cit68] Osmaniye D., Hıdır A., Sağlık B. N., Levent S., Özkay Y., Kaplancıklı Z. A. (2022). Chem. Biodiversity.

[cit69] Taglieri L., Saccoliti F., Nicolai A., Peruzzi G., Madia V. N., Tudino V., Messore A., Di Santo R., Artico M., Taurone S., Salvati M., Costi R., Scarpa S. (2020). Invest. New Drugs.

[cit70] Chi L., Wang H., Yu F., Gao C., Dai H., Liu L., Wang Z., Dong Y., Liu H., Zhang Q. (2023). Med. Chem. Res..

[cit71] Nemr M. T. M., AboulMagd A. M. (2020). Bioorg. Chem..

[cit72] Lin R.-J., Xie L., Gao T.-Y., Yang Y.-Z., Huang L., Cheng K., Chen Z. (2025). Eur. J. Med. Chem..

[cit73] Gao W., Hu H., Dai L., He M., Yuan H., Zhang H., Liao J., Wen B., Li Y., Palmisano M., Traore M. D. M., Zhou S., Sun D. (2022). Acta Pharm. Sin. B.

[cit74] Mehta K., Maass C., Cucurull-Sanchez L., Pichardo-Almarza C., Subramanian K., Androulakis I. P., Gobburu J., Schaller S., Sherwin C. M. (2025). ACS Pharmacol. Transl. Sci..

[cit75] Niazi S. K., Mariam Z. (2024). Pharmaceuticals.

[cit76] Mehran M. J., Mohammadzadeh S., Bolideei M., Barzigar R., Haider K. H., Jadgal N., Bahrami Y. (2026). Drug Dev. Res..

[cit77] Ferreira F. J. N., Carneiro A. S. (2025). ACS Omega.

[cit78] Venkatapurapu S. P., Clegg L., Nowojewski A., Kimko H., Olabode D., Sawant-Basak A., Vishwanathan K. (2026). Drug Discovery Today.

[cit79] Khoshfekr Rudsari H., Tseng B., Zhu H., Song L., Gu C., Roy A., Irajizad E., Butner J., Long J., Do K.-A. (2025). Front. Digit. Health.

[cit80] Zhang B., Zhang L., Chen Q., Jin Z., Liu S., Zhang S. (2023). Commun. Med..

[cit81] Hassanzadeh H., Karimi S., Nguyen A. (2020). J. Biomed. Inf..

[cit82] Satheeskumar R. (2025). Intell. Pharm..

[cit83] Wang S., Yang J.-G., Rong K., Li H.-H., Wu C., Tang W. (2024). Int. J. Mol. Sci..

[cit84] Sun D., Gao W., Hu H., Zhou S. (2022). Acta Pharm. Sin. B.

[cit85] Sun D., Macedonia C., Chen Z., Chandrasekaran S., Najarian K., Zhou S., Cernak T., Ellingrod V. L., Jagadish H. V., Marini B., Pai M., Violi A., Rech J. C., Wang S., Li Y., Athey B., Omenn G. S. (2024). J. Med. Chem..

